# Fluorinated Derivatives of AG-881 for Positron Emission Tomography Detection of Mutated Isocitrate Dehydrogenase 1

**DOI:** 10.3390/ph19050660

**Published:** 2026-04-23

**Authors:** Thu Hang Lai, Sladjana Dukić-Stefanović, Winnie Deuther-Conrad, Aurélie Maisonial-Besset, Rodrigo Teodoro, Magali Toussaint, Barbara Wenzel

**Affiliations:** 1 Department of Research and Development, ROTOP Pharmaka GmbH, 01328 Dresden, Germany; publications@rotop-pharmaka.de; 2 Department of Experimental Neurooncological Radiopharmacy, Institute of Radiopharmaceutical Cancer Research, Research Site Leipzig, Helmholtz-Zentrum Dresden-Rossendorf (HZDR), 04318 Leipzig, Germany; s.dukic-stefanovic@hzdr.de (S.D.-S.); w.deuther-conrad@hzdr.de (W.D.-C.); rodrigo.teodoro@lantheus.com (R.T.); 3 UMR INSERM 1240, Molecular Imaging and Theranostic Strategies, University of Clermont Auvergne, 63000 Clermont-Ferrand, France; aurelie.maisonial@uca.fr

**Keywords:** vorasidenib, radiofluorination, PET imaging

## Abstract

**Background/Objectives**: Since 2016, the mutation of isocitrate dehydrogenase 1 (mIDH1) enzymes has become a major molecular marker for glioma classification and diagnosis. Moreover, the recent success of the INDIGO clinical trial on **AG-881** (vorasidenib^®^), an aminotriazine-based mutated IDH1/2 inhibitor (IC_50_ = 6 nM/12 nM), validated the need for noninvasive detection of mIDH1 in brain tumors. This work is based on developing a series of novel fluorinated analogues of **AG-881** and evaluating their potential in mIDH1 PET detection. **Methods**: The analogues were tested for their potency and then the best candidate was radiofluorinated and used for in vitro cell uptake studies. **Results**: Six analogues (**6**–**11**) were designed and synthesized, but only compound **6** showed nanomolar inhibitory potency towards mIDH1 (IC_50_ = 400 nM). Following successful radiofluorination, in vitro cell uptake studies showed no selective accumulation of [^18^F]**6**. **Conclusions**: This study highlights the critical impact of substituent positioning and halogen substitution within the pyridyl moiety on maintaining inhibitory potency. Further medicinal chemistry research is needed to develop an aminotriazine-based ^18^F-radiolabeled mIDH1 ligand.

## 1. Introduction

Mutation in the metabolic enzymes isocitrate dehydrogenase 1 and 2 (IDH1 and IDH2) has been employed since 2016 as the main diagnostic and prognostic biomarker for adult diffuse glioma [1,2]. These somatic gene mutations involve a heterozygous missense substitution of the purine base guanine at position 395, which typically transitions to adenine (G395A). The most common subtype (90% of the cases) is an arginine-to-histidine substitution (R132H) in the IDH1 isoform (IDH1^R132H^) [3,4,5]. Through the NADPH-dependent conversion of *alpha*-ketoglutarate (*α*-KG) to D-2-hydroxyglutarate (2-HG), these gain-of-function mutations induce neomorphic activity. Its presence in more than 70% of cases [6], its role in gliomagenesis [7,8], and its retention during disease progression have made it a subject of interest for the development of targeted therapies [9,10,11]. In this context, the development of inhibitors of mutated IDH, particularly its most common form (IDH1^R132H^), has rapidly increased [12]. Since 2016, more than eight inhibitors have been tested in phase 1/2 clinical trials for the treatment of gliomas (**AG-120**, **AG-881**, **FT-2102**, **DS-1001**, **LY-3410738**, **BAY1436032**, **IDH305** and **HMPL-306**) [12].

Among them, **AG-881** (vorasidenib, Voranigo^®^), an allosteric inhibitor of mIDH1/IDH2 containing an aminotriazine scaffold (IC_50_ = 6 nM for IDH1^R132H^ and 12 nM for IDH2^R140Q^) [13], was shown to significantly prolong progression-free survival in patients with grade 2 IDH-mutant glioma in the phase 3 INDIGO trial and led to regulatory approval by both the FDA and the EMA [14,15,16]. This success highlights the need for additional tools to support molecularly guided treatment strategies. The noninvasive assessment of IDH mutation status and therapeutic response remains limited. Positron emission tomography (PET) imaging using radiotracers with sufficient brain penetration and target engagement could be a valuable approach for longitudinal monitoring, molecular stratification and evaluation of therapeutic efficacy in patients with IDH-mutant gliomas.

**Figure 1:**
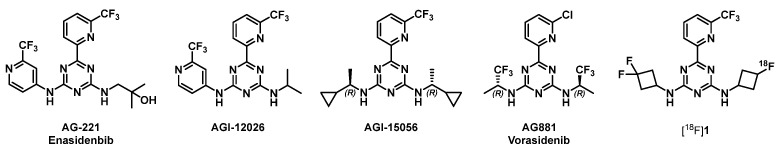
Chemical structures of selected clinically and preclinically investigated aminotriazine-based mutant IDH inhibitors and the first reported PET radiotracer.

Although small-molecule mIDH1 inhibitors are an attractive starting point, structural constraints often hinder direct radiolabeling, requiring the development of modified derivatives [17,18,19]. To date, only one PET radiotracer based on the aminotriazine scaffold has been reported, namely the ^18^F-labeled triazinediamine analogue [^18^F]**1** (Figure 1). Compound **1** exhibited high inhibitory potency and binding affinity toward IDH1^R132H^ (IC_50_ = 54 nM, K_d_ = 40 nM), as well as specific tumor uptake in IDH1-mutant glioma xenografts. However, elevated bone uptake indicative of in vivo defluorination was observed, highlighting limitations in metabolic stability and the need for further optimization of the chemical backbone [20].

Heteroaryl derivatives and modified aliphatic amino substituents of the aminotriazine **AG-881** scaffold were already selected to explore steric and electronic tolerance within the allosteric mutant IDH1 binding pocket while enabling PET tracer development [13]. According to crystallographic studies, the aminotriazine core is stabilized by hydrogen bonds with Q277 and by hydrophobic interactions with V121, W124, I251, V255, M259, and W267 [21]. Directional carbon–fluorine–oxygen and tetrel-type interactions between CF_3_-substituted pyridyl groups and Asp237/Asp312 are critical for high-affinity binding, as demonstrated for **AG-881** and the structurally related inhibitors **AGI-221** (enasidenib), **AGI-12026** and **AGI-15056**, as well as for the previously reported radiotracer [^18^F]**1** (Figure 1) [13,20,22,23,24]. Structure–activity relationship (SAR) analyses further indicate that the removal or polar substitution of the C-6 trifluoromethyl group markedly reduces potency, whereas selected halogen substitutions are partially tolerated [13,25]. These findings suggest that chemically modified fluorinated analogues may retain sufficient target interactions to support PET radiotracer development. Accordingly, a small series of novel **AG-881** analogues bearing different fluorinated pyridine substituents was synthesized and their inhibitory potency toward the R132H mutated form of IDH1 was evaluated. The most potent candidate was selected for radiofluorination and cell uptake studies in living IDH1^R132H^-U251 and IDH1^wt^-U251 cells were performed to evaluate the target binding potential of this compound class.

## 2. Results

### 2.1. Development of Novel AG-881 Derivatives

#### 2.1.1. Design and Synthesis

**Scheme 1:**
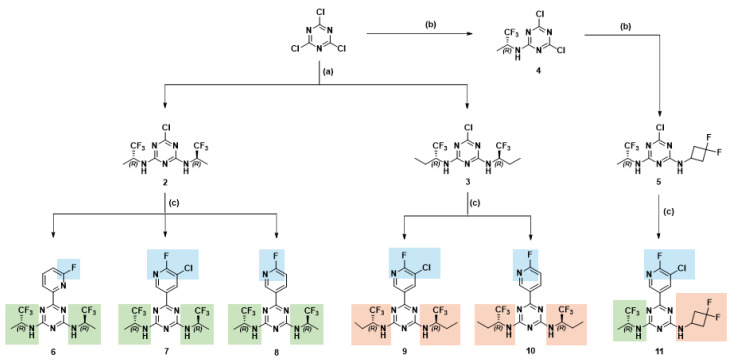
General synthetic route to fluorinated aminotriazine derivatives **6**–**11**. Reaction conditions: (a) trifluoroalkylamine (2 eq), DIPEA, dioxane, 5 h, 50 °C; (b) trifluoroalkylamine (1 eq) or difluorocyclobutylamine (1 eq), DIPEA, dioxane, 1 h, 50 °C; (c) fluoro-substituted pyridine boronate ester (1 eq), Cs_2_CO_3_, Pd(PPh_3_)_4_, dioxane/water (5/1, *v*/*v*), thermal: 2 h, 90 °C or MW: 10 min, 120 °C.

The synthesis of **AG-881** was previously reported in a four-step procedure involving the esterification of 2-chloro-6-trifluoromethylpyridine, cyclization to the corresponding 1,3,5-triazine-2,4-dione, chlorination to the 2,4-dichloro-triazine and final amination with a chiral alkylamine [13]. We developed an alternative and optimized procedure, using 2,4,6-trichloro-1,3,5-triazine as a triazine scaffold (Scheme 1). Sequential *N*-alkylation introduced one or two amino substituents at the triazine core, yielding the symmetric intermediates **2**–**3** and the asymmetric intermediate **5**. The reactivity of the triazine ring decreases with successive substitutions, which facilitates controlled stepwise derivatization. The residual chlorine substituent of these intermediates was then converted via palladium-catalyzed Suzuki–Miyaura cross-coupling with pyridinyl boronate esters, incorporating the heteroaryl substituent at the triazine scaffold. The reaction can be performed either thermally at 90 °C for 2 h or under microwave irradiation at 120 °C for 10 min. Using this strategy, a total of six novel derivatives **6**–**11** were successfully synthesized. Due to this optimized approach, the number of synthetic steps was reduced and efficient late-stage functionalization was achieved, providing rapid access to both symmetric and asymmetric pyridinyl-triazine derivatives.

#### 2.1.2. Biological Evaluation

The synthesized **AG-881** derivatives were evaluated for their inhibitory activity against IDH1^R132H^. In vitro inhibition assays were performed, and the IC_50_ values are reported in Table 1.

### 2.2. Development and Characterization of [^18^F]***6***

#### 2.2.1. Manual Procedures for the Investigation of Reaction Parameters

**Figure 2:**
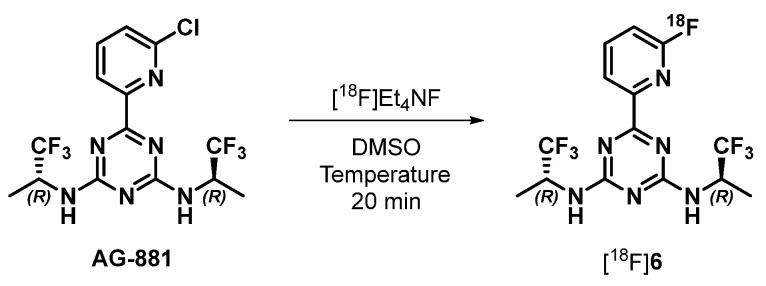
Radiofluorination of **AG-881** to produce [^18^F]**6**.

Based on its potency towards IDH1^R132H^, compound **6** was selected as the most promising candidate for PET radiotracer development. Radiofluorination was achieved via heteroaromatic nucleophilic substitution using [^18^F]fluoride and **AG-881** as a chlorinated precursor compound (Figure 2). Although the chlorine substituent in **AG-881** is a less favorable leaving group in comparison to, e.g., nitro groups, this precursor was selected due to its commercial availability, thereby avoiding expensive and time-consuming precursor synthesis. In initial experiments, the [^18^F]F^−^/K_222_/K_2_CO_3_ system was tested with a constant precursor amount of 2.0 mg (4.8 µmol). The anhydrous polar aprotic solvents DMSO, DMF, MeCN and DMI were evaluated at various temperatures (100–200 °C) and reaction times (5–20 min, see Table S1 in SI). The highest radiochemical conversion (RCC), determined by radio-TLC and confirmed by radio-HPLC, was 32% when DMSO was used at 190 °C for 20 min. Under the same conditions, the use of [^18^F]Et_4_NF as the fluorination agent resulted in a slight increase in RCC (43%).

**Figure 3:**
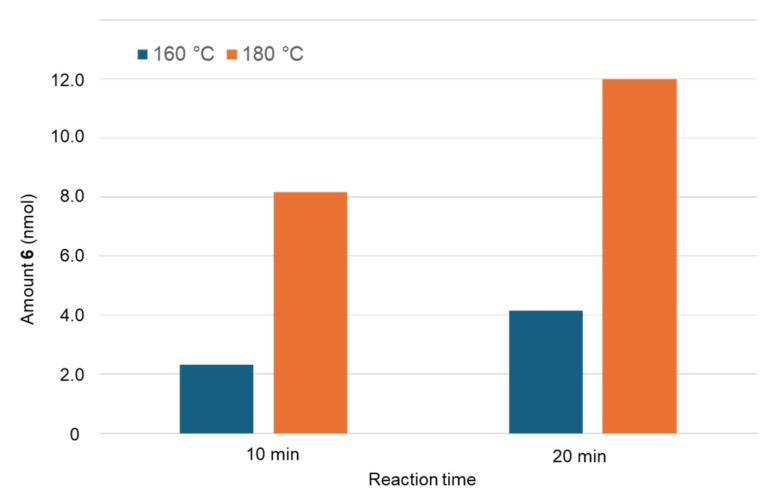
Temperature- and time-dependent formation of compound **6** from **AG-881** (1.0 mg, 2.4 µmol) under radiolabeling conditions.

RP-HPLC analysis of the reaction mixtures, obtained with a reaction temperature above 160 °C, revealed unusually high amounts of non-radioactive **6** detected on the UV chromatograms. To exclude contamination of the precursor with fluorinated species, **AG-881** was analyzed by ^19^F NMR, confirming the absence of additional fluorine-containing compounds. To investigate whether the precursor **AG-881** can release fluorine from its CF_3_ groups under radiolabeling conditions, non-radioactive reactions at 160 and 180 °C were performed under the same conditions as in the radiolabeling experiments. The formation of compound **6** as a function of the reaction time was analyzed by RP-HPLC. As shown in Figure 3, approximately three times more product was formed at 180 °C in comparison to 160 °C. Furthermore, the influence of the base Et_4_NHCO_3_ (used at the beginning of the radiolabeling process to elute [^18^F]fluoride from the anion exchange cartridge and generate [^18^F]Et_4_NF), was investigated by heating the precursor in DMSO alone at 180 °C. Under these conditions, no formation of compound **6** was observed within 20 min.

Finally, a radiolabeling temperature of 160 °C was used despite a lower RCC, in order to prevent the excessive formation of non-radioactive compound **6**, which would result in very low molar activity.

#### 2.2.2. Automated Radiosynthesis of [^18^F]**6**

**Figure 4:**
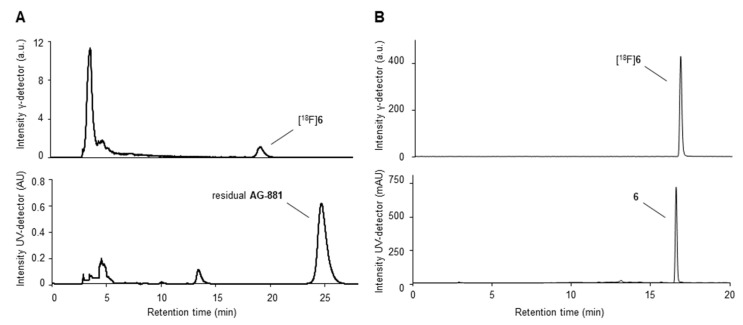
(**A**) Semi-preparative radio- and UV-HPLC chromatogram of [^18^F]**6** (conditions: Reprosil-Pur C18-AQ, 250 × 10 mm, 55% MeCN in aq. 20 mM NH_4_OAc (*v*/*v*), 4.0 mL/min). (**B**) Analytical radio- and UV-HPLC chromatogram of the final product of [^18^F]**6** spiked with the non-radioactive reference **6** (conditions: Reprosil-Pur C18-AQ, 150 × 3.0 mm, 3 µm, gradient with an eluent mixture of MeCN in 20 mM aq. NH_4_OAc, 0.4 mL/min).

Based on the manual experiments, the radiosynthesis of [^18^F]**6** was subsequently automated using the TRACERlab FX2 N synthesis module (GE Healthcare). The procedure is described in detail in the experimental part of this paper. Briefly, [^18^F]fluoride was first trapped and then eluted from the anion exchange cartridge followed by the labeling reaction of the azeotropically dried [^18^F]Et_4_NF with the precursor **AG-881** in DMSO for 20 min at 160 °C. For the isolation of [^18^F]**6**, the crude reaction mixture was diluted with a mixture of MeCN and water and directly applied to a semi-preparative RP-HPLC system using 55% MeCN in aq. 20 mM NH_4_OAc (*v*/*v*) as the eluent. The radiotracer fraction was collected at a retention time of approximately 20 min (chromatogram shown in Figure 4A) and subjected to solid-phase extraction (SPE) on a C18 cartridge.

The isolated radiotracer eluate was transferred out of the hot cell, concentrated under argon flow and formulated in sterile isotonic saline containing 10% ethanol. The entire process took approximately 85 min and [^18^F]**6** could be synthesized with a high radiochemical purity of ≥99%, a radiochemical yield of 6.9 ± 1.4% (*n* = 4, EOB) and molar activities in the range of 65–83 GBq/µmol (*n* = 4, EOS). With this procedure, activity yields in the range of 600–1100 MBq were obtained at starting activities of 25–27 GBq. To confirm the identity of [^18^F]**6**, the corresponding reference compound **6** was added to a sample of the final product and inspected by analytical radio- and UV-HPLC (Figure 4B).

The in vitro stability of [^18^F]**6** was investigated by incubation in isotonic saline (pH 6.5), phosphate-buffered saline (PBS, pH 7.4) and cell culture medium (RPMI 1640, pH 7.4) containing 10% FBS at 37 °C for up to 4 h. Samples were taken after 1, 2, 3 and 4 h and analyzed by radio-HPLC. During this time period, no degradation or defluorination of the radioligand was observed. The chromatograms are shown in Figure S12 in the Supplementary Information.

#### 2.2.3. In Vitro Studies of [^18^F]**6**

**Figure 5:**
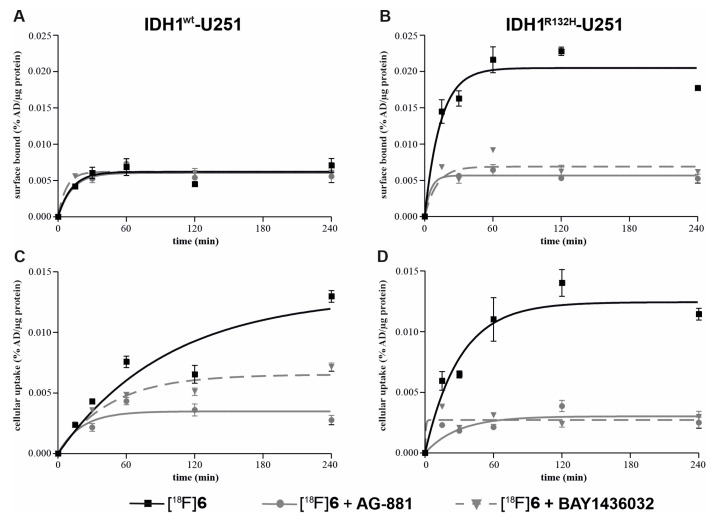
In vitro cellular uptake of [^18^F]**6** in IDH1^wt^-U251 and IDH1^R132H^-U251 cells. (**A**) Surface-bound fraction in IDH1^wt^-U251 cells, (**B**) surface-bound fraction in IDH1^R132H^-U251 cells, (**C**) internalized fraction in IDH1^wt^-U251 cells, and (**D**) internalized fraction in IDH1^R132H^-U251 cells. Cells were co-incubated with vehicle (0.01% DMSO; control), **AG-881** or **BAY1436032** (1 µM) and [^18^F]**6** (6.36 nM). Results are presented as % of applied dose of the radioligand per µg protein (% AD/µg protein) vs. incubation time. All curves fitted best with a one-site model. Data shown are representative curves from one of two experiments. Each experiment (*n* = 2) was done in triplicate.

The kinetics of cellular uptake of [^18^F]**6** were first investigated in living IDH1^wt^-U251 and IDH1^R132H^-U251 cells by incubation with [^18^F]**6** at various time points (15, 30, 60, 120 and 240 min). The results from the cell uptake studies suggest that [^18^F]**6** interacts with the cell membrane of both cell lines at a comparable rate, as reflected by similar association half-lives of the surface-bound fraction of 8.3 min for IDH1^wt^-U251 and 9.6 min for IDH1^R132H^-U251 (Figure 5A,B). In contrast, the internalization of the ligand was slower in IDH1^wt^-U251 cells than in IDH1^R132H^-U251 cells (67 min vs. 21 min), while the total amount of internalized activity was comparable after 4 h of incubation (Figure 5C,D).

The specificity of cellular uptake of [^18^F]**6** was then investigated in a blocking study by co-incubation of IDH1^wt^-U251 and IDH1^R132H^-U251 cells with their respective inhibitors **AG-881** and **BAY1436032**. An initial experiment with a range of concentrations of both blocking agents (1 nM, 10 nM, 100 nM, and 1 µM) has demonstrated a measurable effect only at the highest concentration of 1 µM. The curves in Figure 5C,D show that internalization was blocked by 1 µM **AG-881** or **BAY1436032** in both cell lines to similar levels.

## 3. Discussion

Developing an ^18^F-labeled PET tracer that targets mutant IDH1 requires balancing target affinity with chemical accessibility for radiolabeling. A small series of derivatives was synthesized to study the SAR by introducing three different fluorinated pyridyl ring moieties with fluorine positioned next to nitrogen to enable straightforward fluorine-18 labeling via nucleophilic aromatic substitution. In compound **6**, the chloropyridine substituent of the triazine core of **AG-881** was directly replaced by the corresponding fluoropyridine. With this substitution, the high lipophilicity of **AG-881** was slightly reduced from a logD_7.4_ value of 5.34 to 4.85, which might be beneficial with respect to a future application as brain PET tracer. However, the substitution also provoked a reduction in potency (IC_50_ = 405 nM), although analogous halogen and CF_3_-group substituted pyridines at this position have been described as being favorable for binding [13]. This reduced potency likely arises from a combination of several factors: (i) the weaker halogen-bonding ability of fluorine relative to chlorine diminishes the interaction with the D273 carbonyl [13], (ii) its higher electronegativity may withdraw electron density from the pyridine ring, altering π–π stacking and hydrogen-bonding interactions, (iii) the smaller van der Waals radius reduces hydrophobic contacts, and (iv) steric effects related to substituent orientation may impact allosteric pocket interactions.

To investigate this effect and maintain, for example, the potential halogen-bonding interaction with the enzyme, the derivatives **7**, **9** and **11** were synthesized with a 5-chloro-6-fluoropyridyl moiety. However, this modification led to a further decrease in potency with IC_50_ values of around 1000 nM. For compounds **9** and **11**, the amino side chains were also varied through chain elongation and asymmetric substitution to assess steric tolerance within the hydrophobic pocket. The minimal change in potency relative to **7** suggests that the binding pocket can tolerate moderate side-chain variations, indicating that steric hindrance is not the main factor. It seems that differences in inhibitory potency are driven by the electronic properties of the pyridyl substituents and their impact on key interactions.

Using the 6-fluoropyridin-3-yl moiety without chlorine substitution, two derivatives, **8** and **10**, were synthesized to directly evaluate the influence of the nitrogen–fluorine unit position in the pyridine ring in comparison to the corresponding chlorinated derivatives **7** and **9**. The removal of chlorine resulted in a complete loss of potency (IC_50_ > 10,000 nM). Compared with **6**, where the nitrogen–fluorine unit is in the *ortho*/*meta* position to C-6 of the triazine core, the *meta*/*para* orientation in **8** and **10** appears unfavorable for target binding.

SAR analysis of synthesized fluorinated heteroaryl and amino-substituted derivatives demonstrated that the position of the nitrogen–fluorine unit within the pyridyl ring is critical, and that a pyridyl substituent with chlorine (or CF_3_) in the *meta* position relative to C-6 of the triazine ring is essential for binding. Although substitution with fluorine at this position reduces potency, the new derivative **6** remained of interest. In this context, compound **6** was selected not as an optimized inhibitor, but as a representative scaffold to study the radiochemical feasibility and in vitro behavior of radiotracers derived from this class of compounds. The preserved aminotriazine core and retained key binding interactions provide a structurally robust framework, while the presence of accessible fluorinated motifs enables practical incorporation of fluorine-18. Thus, compound **6** served as a suitable model for evaluating radiolabeling strategies and assessing tracer-related properties in vitro, supporting further optimization of this chemical class.

The choices of precursor and reaction conditions strongly influence radiochemical conversion and side-product formation. Although the chlorine leaving group is less reactive, **AG-881** was chosen for its commercial availability to avoid time-consuming precursor synthesis. To achieve good radiochemical conversions, high temperatures in the range of 190–200 °C were required; however, they resulted in insufficient molar activities due to concomitant formation of non-radioactive **6**. Non-radioactive experiments demonstrated that higher temperatures accelerated the formation of **6** via the thermal release of fluoride from the CF_3_ groups, whereas no product was formed in DMSO without a base, indicating possible *β*-elimination under slightly basic conditions or decomposition of a part of the precursor present in large excess. Even though only a small fraction of **AG-881** underwent this process, it was sufficient to substantially reduce the molar activity of [^18^F]**6**, necessitating a reduction in reaction temperature to achieve the best compromise between labeling efficiency and acceptable molar activity. These results highlight the importance of a proper leaving group for radiofluorination of this class of compounds to ensure high radiochemical yield and molar activity. Despite the lower RCC due to the reduced labeling temperature needed, the radiosynthesis of [^18^F]**6** was successfully implemented on an automated radiosynthesis module. Using higher starting activities, the approach enabled reproducible production of [^18^F]**6** with sufficient radiochemical yield and good molar activities for preliminary in vitro evaluation. Indeed, with molar activities of 65–83 GBq/µmol, the final chemical concentration for the in vitro studies is in the range of 4.8 to 6.4 nM, at which no significant target occupancy is expected.

The comparable cellular uptake of [^18^F]**6** in both IDH1^wt^-U251 and IDH1^R132H^-U251 cells, together with the blocking effects of the selective IDH1^R132H^ inhibitor **BAY1436032** and the pan-mutant IDH1/2 inhibitor **AG-881**, indicated that, despite moderate target-specific inhibitory potency, the radioligand showed no specificity for IDH1^R132H^.

## 4. Materials and Methods

### 4.1. Chemistry

#### 4.1.1. Chemicals and Reagents

All chemicals and reagents were purchased from commercially available sources and used without further purification. Moisture-sensitive reactions were conducted under dry argon using anhydrous solvents. **AG-881** was purchased from BLDpharm (Shanghai, China) or MCE (Monmouth Junction, NJ, USA) and used as a precursor or reference, respectively. Its identity and purity were confirmed by NMR spectroscopy. ^1^H NMR (400 MHz, DMSO-*d*_6_): *δ* = 8.66–8.10 (m, 3H), 8.03 (t, *J* = 7.8 Hz, 1H), 7.76–7.59 (m, 1H), 5.18–5.03 (m, 1H), 5.03–4.79 (m, 1H), and 1.34 (dd, *J* = 7.2, 2.9 Hz, 6H). ^19^F NMR (377 MHz, DMSO-*d*_6_): *δ* = −75.49–−75.82 (m, 1F). ^13^C NMR (101 MHz, DMSO-*d*_6_): *δ* = 168.81 (d, *J* = 30.5 Hz), 168.10–165.01 (m), 154.45, 150.20, 140.66 (d, *J* = 8.7 Hz), 126.52 (d, *J* = 12.4 Hz), 130.59–121.45 (m), 122.72 (d, *J* = 42.9 Hz), 49.11–44.90 (m), and 13.49 (d, *J* = 15.3 Hz).

#### 4.1.2. Analytics

Reaction progress was monitored by thin-layer chromatography (TLC) on Alugram^®^ SIL G/UV254 precoated plates (Macherey-Nagel, Düren, Germany), and spots were visualized under UV light. Product purification was performed by flash column chromatography using silica gel (40–63 μm) from VWR International Chemicals (Darmstadt, Germany).

The purity of all tested compounds (>95%) was determined by a Liquid Chromatography–Mass Spectrometry (LC-MS) system with diode array detection (DAD) using a Dionex Ultimate 3000 system coupled to an MSQ 000 low-resolution mass spectrometer (Thermo Fisher Scientific Inc., Waltham, MA, USA). A Reprosil-Pur 120 C18-AQ column (150 × 3.0 mm, 3 µm; Dr. Maisch HPLC GmbH, Ammerbuch, Germany) was used with MeCN/20 mM NH_4_OAc (aq.) as the eluent mixture at a flow rate of 0.7 mL·min^−1^. Eluent A consisted of 5% MeCN in 20 mM NH_4_OAc (aq., *v*/*v*) and eluent B of 80% MeCN in 20 mM NH_4_OAc (aq., *v*/*v*). The gradients were 0–1.25 min, 100% A; 1.25–5 min, 0–100% B; 5–12 min, 100% B; 12–12.5 min, 100–0% B; and 12.5–15 min, 100% A. UV detection was performed at λ = 254 nm.

Nuclear magnetic resonance (NMR) spectra were recorded on Varian MERCURY plus 400 (Agilent Technologies, Santa Clara, CA, USA) or Bruker AVANCE III HD 400 spectrometers (Bruker Corporation, Billerica, MA, USA) 400 MHz for ^1^H, 100 MHz for ^13^C, and 376 MHz for ^19^F, see Figures S1–S11 in SI). Chemical shifts (δ) are reported in parts per million (ppm) relative to internal tetramethylsilane (TMS) and coupling constants (*J*) are given to 0.1 Hz. High-resolution mass spectra (HRMS) were obtained using an ESI-TOF micrOTOF mass spectrometer (Bruker Daltonik GmbH, Bremen, Germany) coupled to an Agilent 1100 HPLC system (Agilent Technologies, Santa Clara, CA, USA; isocratic pump, autosampler) and operated with otofControl 3.4 and HyStar 3.2-LC/MS, enabling accurate high-resolution mass measurements.

The logD values were calculated with the ACD/Labs software version 12.5.

#### 4.1.3. Chemical Synthesis

**General Procedure A**—Symmetric derivatives: 2,4,6-Trichloro-1,3,5-triazine (1.0 eq, 5.42 mmol) and (*R*)-1,1,1-trifluoropropan-2-amine hydrochloride or (*R*)-1,1,1-trifluoro-2-butylamine hydrochloride (2.0 eq, 10.84 mmol) were dissolved in dioxane (30 mL). DIPEA (5.0 eq, 27.11 mmol) was added at 0 °C. The reaction mixture was stirred for 30 min at room temperature and then for 5 h at 50 °C. After completion, 50 mL of water was added and the aqueous phase was extracted with EtOAc (3 × 20 mL). The combined organic layers were washed with brine (20 mL), dried over anhydrous MgSO_4_, filtered and concentrated under reduced pressure. The crude product was purified by flash column chromatography on silica gel using a gradient of petroleum ether/EtOAc 12:1 → 7:1, affording the corresponding products **2** and **3** as white solids.

6-chloro-*N*^2^,*N*^4^-bis((*R*)-1,1,1-trifluoropropan-2-yl)-1,3,5-triazine-2,4-diamine (**2**). Yield: 69%. TLC (silica gel, petroleum ether/EtOAc, 9/1, *v*/*v*): R_f_ = 0.26. ^1^H NMR (400 MHz, DMSO-*d*_6_): δ = 8.73–8.22 (m, 2H), 5.03–4.66 (m, 2H), 1.43–1.20 (m, 6H). ^19^F NMR (377 MHz, DMSO-*d*_6_): δ = −72.45–−80.26 (m, 1F). ^13^C NMR (101 MHz, DMSO-*d*_6_): δ = 168.64 (d, *J* = 16.9 Hz), 166.94–164.06 (m, 2C), 131.33–117.21 (m, 2C), 47.10 (q, *J* = 30.7 Hz, 2C), 13.27 (2C). LC-MS (ESI^−^, UV 254 nm): *t*_R_ = 7.64 min, *m*/*z* 336.1 ([M-H]^−^), purity > 98%.

6-chloro-*N*^2^,*N*^4^-bis((*R*)-1,1,1-trifluorobutan-2-yl)-1,3,5-triazine-2,4-diamine (**3**). Yield: 39%. TLC (silica gel, petroleum ether/EtOAc, 8/1, *v*/*v*): R_f_ = 0.25. ^1^H NMR (400 MHz, DMSO-*d*_6_): δ = 9.65 (d, *J* = 9.1 Hz, 1H), 4.61 (dddd, *J* = 16.6, 15.0, 7.6, 3.7 Hz, 1H), 1.80 (dtd, *J* = 14.8, 7.4, 3.8 Hz, 1H), 1.66 (ddq, *J* = 14.4, 10.7, 7.3 Hz, 1H), 0.93 (t, *J* = 7.3 Hz, 3H). ^19^F NMR (377 MHz, DMSO-*d*_6_): δ = −74.26–−74.58 (m, 1F). ^13^C NMR (101 MHz, DMSO-*d*_6_): δ = 168.06 (d, *J* = 10.0 Hz), 166.54–164.83 (m, 2C), 129.49–120.16 (m, 2C), 54.18–48.43 (m, 2C), 22.14–16.38 (m, 2C), 12.87–0.72 (m, 2C). LC-MS (ESI^−^, UV 254 nm): *t*_R_ = 7.98 min, *m*/*z* 366.1 ([M-H]^−^), purity > 99%.

**General Procedure B**—Asymmetric derivatives: 2,4,6-Trichloro-1,3,5-triazine (1.0 eq, 5.42 mmol) and (*R*)-1,1,1-trifluoropropan-2-amine hydrochloride (1.0 eq, 5.42 mmol) were dissolved in dioxane (30 mL). DIPEA (3.0 eq, 16.27 mmol) was added at 0 °C. The reaction mixture was stirred for 30 min at room temperature and then for 1 h at 50 °C. After completion, 50 mL of water was added and the aqueous phase was extracted with EtOAc (3 × 20 mL). The combined organic layers were washed with brine (20 mL), dried over anhydrous MgSO_4_, filtered and concentrated under reduced pressure. The crude product was purified by flash column chromatography on silica gel using a gradient of hexane/EtOAc 1:0 → 10:1, affording the desired intermediate, (*R*)-4,6-dichloro-*N*-(1,1,1-trifluoropropan-2-yl)-1,3,5-triazin-2-amine **4**, as a yellow oil. Subsequently, the intermediate **4** (1.0 eq, 2.30 mmol) and 3,3-difluorocyclobutan-1-amine hydrochloride (1.0 eq, 2.30 mmol) were dissolved in dioxane (6 mL). DIPEA (3.0 eq, 6.89 mmol) was added at 0 °C. The reaction mixture was stirred for 30 min at room temperature and then for 1 h at 50 °C. After completion, 50 mL of water was added and the aqueous phase was extracted with EtOAc (3 × 20 mL). The combined organic layers were washed with brine (20 mL), dried over anhydrous MgSO_4_, filtered and concentrated under reduced pressure. The crude product was purified by flash column chromatography on silica gel using a gradient of hexane/EtOAc 7:1 → 3:1, affording product **5** as a white solid.

(*R*)-4,6-dichloro-*N*-(1,1,1-trifluoropropan-2-yl)-1,3,5-triazin-2-amine (**4**). Yield: 84%. TLC (silica gel, petroleum ether/EtOAc, 9/1, *v*/*v*): R_f_ = 0.38. ^1^H NMR (400 MHz, CDCl_3_): δ = 5.82 (d, *J* = 10.7 Hz, 1H), 4.91 (dp, *J* = 9.9, 6.9 Hz, 1H), 1.45 (d, *J* = 7.0 Hz, 3H). ^19^F NMR (377 MHz, CDCl_3_): δ = −77.52 (d, *J* = 6.9 Hz, 1F). ^13^C NMR (101 MHz, CDCl_3_): δ = 171.22 (d, *J* = 60.3 Hz), 166.12 (2C), 124.88 (q, *J* = 281.4 Hz), 48.74 (q, *J* = 32.1 Hz), 14.29. LC-MS (ESI^−^, UV 254 nm): *t*_R_ = 7.64 min, *m*/*z* 259.1 ([M-H]^−^), purity > 98%.

(*R*)-6-chloro-*N*^2^-(3,3-difluorocyclobutyl)-*N*^4^-(1,1,1-trifluoropropan-2-yl)-1,3,5-triazine-2,4-diamine (**5**). Yield: 98%. TLC (silica gel, petroleum ether/EtOAc, 4/1, *v*/*v*): R_f_ = 0.46. ^1^H NMR (400 MHz, DMSO-*d*_6_): δ = 8.60–8.36 (m, 2H), 4.82 (ddt, *J* = 48.6, 14.9, 7.5 Hz, 1H), 4.28–4.04 (m, 1H), 3.06–2.79 (m, 2H), 2.79–2.55 (m, 2H), 1.30 (d, *J* = 7.0 Hz, 3H). ^19^F NMR (377 MHz, DMSO-*d*_6_): δ = −75.86 (q, *J* = 8.3 Hz, 3F), −80.50–−84.50 (m, 1F), −95.08–−98.78 (m, 1F). ^13^C NMR (101 MHz, DMSO-*d*_6_): δ = 170.11–166.32 (m), 166.32–163.23 (m, 2C), 128.74–112.79 (m, 2C), 47.03 (q, *J* = 30.4 Hz), 45.09–40.66 (m, 2C), 37.15–32.72 (m), 13.29. LC-MS (ESI^−^, UV 254 nm): *t*_R_ = 7.64 min, *m*/*z* 330.2 ([M-H]^−^), purity > 98%.

**General Procedure C**—Novel derivatives: The corresponding dialkylated 1,3,5-triazine-2,4-diamine (1 eq, 0.39 mmol) and fluoro-substituted pyridine boronate ester (1 eq, 0.39 mmol) were introduced in a sealed reaction vial and dissolved in dioxane (2.5 mL) and water (0.5 mL). Cs_2_CO_3_ (2.5 eq, 0.97 mmol) and Pd(PPh_3_)_4_ (0.1 eq, 0.04 mmol) were then added to the solution. The reaction mixture was heated either thermally for 2 h at 90 °C or under microwave (MW) irradiation for 10 min at 120 °C. Upon complete conversion, the reaction was quenched with water (20 mL), and the aqueous phase was extracted with EtOAc (3 × 20 mL). The combined organic layers were washed with brine (20 mL), dried over anhydrous MgSO_4_, filtered, and concentrated under reduced pressure. The crude product was purified by flash column chromatography on silica gel using a gradient of hexane/EtOAc 20:1 → 4:1, affording the desired product as a white solid.

6-(5-chloro-6-fluoropyridin-3-yl)-*N*^2^,*N*^4^-bis((*R*)-1,1,1-trifluoropropan-2-yl)-1,3,5-triazine-2,4-diamine (**6**). Yield: 30% (thermal). TLC (silica gel, petroleum ether/EtOAc, 4/1, *v*/*v*): R_f_ = 0.19. ^1^H NMR (400 MHz, DMSO-*d*_6_): δ = 8.56–8.20 (m, 2H), 8.20–8.09 (m, 1H), 7.37 (ddd, *J* = 10.7, 6.9, 2.5 Hz, 1H), 5.27–4.74 (m, 2H), 1.34 (dd, *J* = 7.2, 3.4 Hz, 6H). ^19^F NMR (377 MHz, DMSO-*d*_6_): δ = −66.57–−68.15 (m, 1F), −75.11–−76.37 (m, 6F). ^13^C NMR (101 MHz, DMSO-*d*_6_): δ = 168.75 (d, *J* = 29.7 Hz), 166.07 (d, *J* = 24.7 Hz, 2C), 162.56 (d, *J* = 235.9 Hz), 152.41 (d, *J* = 13.2 Hz), 142.94 (d, *J* = 7.4 Hz), 131.69–121.80 (m, 2C), 121.42 (d, *J* = 41.6 Hz), 112.93–111.57 (m), 48.45–44.86 (m, 2C), 13.40 (2C). LC-MS (ESI^−^, UV 254 nm): *t*_R_ = 8.63 min, *m*/*z* 396.6 ([M-H]^−^), purity > 97%. HRMS (ESI^+^): *m*/*z* calcd for C_14_H_13_F_7_N_6_ [M + H]^+^ 399.1163, found 399.1201.

6-(6-fluoropyridin-2-yl)-*N*^2^,*N*^4^-bis((*R*)-1,1,1-trifluoropropan-2-yl)-1,3,5-triazine-2,4-diamine (**7**). Yield: 82% (thermal). TLC (silica gel, petroleum ether/EtOAc, 4/1, *v*/*v*): R_f_ = 0.63. ^1^H NMR (400 MHz, MeOD): δ = 9.04 (dd, *J* = 2.0, 1.1 Hz, 1H), 8.83 (t, *J* = 7.7 Hz, 1H), 5.17–5.05 (m, 1H), 4.96 (tq, *J* = 14.8, 7.5 Hz, 1H), 1.40 (d, *J* = 7.1 Hz, 6H). ^19^F NMR (377 MHz, MeOD): δ = −71.22–−71.81 (m, 1F), −78.76 (ddd, *J* = 44.1, 34.4, 7.6 Hz, 6F). ^13^C NMR (101 MHz, MeOD): δ = 168.85 (d, *J* = 22.3 Hz), 167.54 (2C), 161.56 (d, *J* = 240.0 Hz), 147.07 (d, *J* = 14.5 Hz), 141.87, 133.83, 127.40 (q, *J* = 281.2 Hz), 117.88 (d, *J* = 35.2 Hz), 30.74 (2C), 14.17 (d, *J* = 17.3 Hz, 2C). LC-MS (ESI^−^, UV 254 nm): *t*_R_ = 8.89 min, *m*/*z* 431.0 ([M-H]^−^), purity > 95%. HRMS (ESI^+^): *m*/*z* calcd for C_14_H_12_ClF_7_N_6_ [M + H]^+^ 433.0773, found 433.0784.

6-(6-fluoropyridin-3-yl)-*N*^2^,*N*^4^-bis((*R*)-1,1,1-trifluoropropan-2-yl)-1,3,5-triazine-2,4-diamine (**8**). Yield: 96% (thermal). TLC (silica gel, petroleum ether/EtOAc, 4/1, *v*/*v*): R_f_ = 0.37. ^1^H NMR (400 MHz, DMSO-*d*_6_): δ = 9.21–8.95 (m, 1H), 8.85–8.61 (m, 1H), 8.36–7.85 (m, 2H), 7.33 (dt, *J* = 8.3, 3.5 Hz, 1H), 5.18 (dt, *J* = 15.3, 7.7 Hz, 1H), 4.94 (dq, *J* = 23.0, 7.6 Hz, 1H). ^19^F NMR (377 MHz, DMSO-*d*_6_): δ = −65.11–−66.23 (m, 1F), −75.11–−76.15 (m, 6F). ^13^C NMR (101 MHz, DMSO-*d*_6_): δ = 167.88 (d, *J* = 28.4 Hz), 165.69 (2C), 162.76 (d, *J* = 170.4 Hz), 148.00 (d, *J* = 16.5 Hz), 141.61 (d, *J* = 9.6 Hz), 130.53, 132.72–123.29 (m, 2C), 109.54 (d, *J* = 37.8 Hz), 46.87 (d, *J* = 31.3 Hz, 2C), 13.49 (d, *J* = 14.0 Hz, 2C). LC-MS (ESI^−^, UV 254 nm): *t*_R_ = 10.61 min, *m*/*z* 396.6 ([M-H]^−^), purity > 99%. HRMS (ESI^+^): *m*/*z* calcd for C_14_H_13_F_7_N_6_ [M + H]^+^ 399.1163, found 399.1186.

6-(5-chloro-6-fluoropyridin-3-yl)-*N*^2^,*N*^4^-bis((*R*)-1,1,1-trifluorobutan-2-yl)-1,3,5-triazine-2,4-diamine (**9**). Yield: 96% (MW). TLC (silica gel, petroleum ether/EtOAc, 8/1, *v*/*v*): R_f_ = 0.46. ^1^H NMR (400 MHz, DMSO-*d*_6_): δ = 9.18–8.65 (m, 2H), 8.38–7.83 (m, 2H), 5.05 (q, *J* = 9.5 Hz, 1H), 4.90–4.62 (m, 1H), 1.88–1.56 (m, 4H), 1.01–0.88 (m, 6H). ^19^F NMR (377 MHz, DMSO-*d*_6_): δ = −69.32–−70.70 (m, 1F), −73.93–−74.67 (m, 6F). ^13^C NMR (101 MHz, DMSO-*d*_6_): δ = 166.86 (d, *J* = 26.6 Hz), 166.38 (d, *J* = 17.2 Hz, 2C), 161.88–155.28 (m), 145.67 (td, *J* = 42.3, 14.9 Hz), 140.50 (d, *J* = 7.9 Hz), 132.25 (d, *J* = 5.2 Hz), 132.45–118.97 (m, 2C), 117.00–112.08 (m), 52.43 (q, *J* = 29.0 Hz, 2C), 20.36 (d, *J* = 7.7 Hz, 2C), 14.80–2.86 (m, 2C). LC-MS (ESI^−^, UV 254 nm): *t*_R_ = 9.93 min, *m*/*z* 460.5 ([M-H]^−^), purity > 95%. HRMS (ESI^+^): *m*/*z* calcd for C_16_H_16_ClF_7_N_6_ [M + H]^+^ 461.1086, found 461.1102.

6-(6-fluoropyridin-3-yl)-*N*^2^,*N*^4^-bis((*R*)-1,1,1-trifluorobutan-2-yl)-1,3,5-triazine-2,4-diamine (**10**). Yield: 95% (MW). TLC (silica gel, petroleum ether/EtOAc, 8/1, *v*/*v*): R_f_ = 0.30. ^1^H NMR (400 MHz, DMSO-*d*_6_): δ = 9.10 (td, *J* = 23.6, 2.4 Hz, 1H), 8.75 (ttd, *J* = 28.2, 8.2, 2.4 Hz, 1H), 8.32–7.77 (m, 2H), 7.45–7.22 (m, 1H), 5.06–4.89 (m, 1H), 4.89–4.61 (m, 1H), 1.87–1.58 (m, 4H), 1.03–0.81 (m, 6H). ^19^F NMR (377 MHz, DMSO-*d*_6_): δ = −64.98–−66.49 (m, 1F), −73.88–−74.76 (m, 6F). ^13^C NMR (101 MHz, DMSO-*d*_6_): δ = 167.92 (d, *J* = 36.0 Hz), 166.65–166.23 (m, 2C), 164.74 (dd, *J* = 239.7, 13.2 Hz), 147.90 (dd, *J* = 25.3, 16.2 Hz), 141.52 (dd, *J* = 23.7, 8.9 Hz), 130.58 (t, *J* = 5.0 Hz), 130.27–119.61 (m, 2C), 109.52 (d, *J* = 37.8 Hz), 54.57–50.14 (m, 2C), 20.39 (2C, 13.06–4.77 (m, 2C). LC-MS (ESI^−^, UV 254 nm): *t*_R_ = 8.63 min, *m*/*z* 425.2 ([M-H]^−^), purity > 99%. HRMS (ESI^+^): *m*/*z* calcd for C_16_H_17_F_7_N_6_ [M + H]^+^ 427.1476, found 427.1505.

(*R*)-6-(5-chloro-6-fluoropyridin-3-yl)-*N*^2^-(3,3-difluorocyclobutyl)-*N*^4^-(1,1,1-trifluoropropan-2-yl)-1,3,5-triazine-2,4-diamine (**11**). Yield: 71% (thermal). TLC (silica gel, petroleum ether/EtOAc, 4/1, *v*/*v*): R_f_ = 0.46. ^1^H NMR (400 MHz, DMSO-*d*_6_): δ = 9.11–8.62 (m, 2H), 8.39–7.75 (m, 2H), 5.37–4.74 (m, 1H), 4.48–4.13 (m, 1H), 2.96 (dtdd, *J* = 20.5, 13.3, 8.0, 3.8 Hz, 2H), 2.84–2.59 (m, 2H), 1.37–1.27 (m, 3H). ^19^F NMR (377 MHz, DMSO-*d*_6_): δ = −70.10 (ddd, *J* = 100.7, 84.1, 8.9 Hz, 1F), −75.12–−76.63 (m, 3F), −80.82–−83.14 (m, 1F), −94.89–−98.66 (m, 1F). ^13^C NMR (101 MHz, DMSO-*d*_6_): δ = 167.01–166.11 (m), 166.11–164.51 (m, 2C), 159.39 (dd, *J* = 237.6, 13.8 Hz), 147.24–143.55 (m), 140.32 (d, *J* = 13.2 Hz), 132.43 (t, *J* = 5.7 Hz), 128.36–116.60 (m, 3C), 115.96 (dd, *J* = 35.4, 8.1 Hz), 46.74 (q, *J* = 30.3 Hz), 42.06 (q, *J* = 27.4 Hz, 2C), 37.14–33.63 (m), 13.53 (d, *J* = 19.5 Hz). LC-MS (ESI^−^, UV 254 nm): *t*_R_ = 8.83 min, *m*/*z* 425.2 ([M-H]^−^), purity > 96%. HRMS (ESI^+^): *m*/*z* calcd for C_15_H_13_ClF_6_N_6_ [M + H]^+^ 427.0867, found 427.0867.

### 4.2. Radiochemistry

#### 4.2.1. General

No-carrier-added [^18^F]fluoride was produced via the [^18^O(p,n)^18^F] nuclear reaction by irradiation of an [^18^O]H_2_O target (Hyox 18 enriched water, Rotem Industries Ltd., Mishor Yamin, Israel) on a Cyclone 18/9 (IBA RadioPharma Solutions, Louvain-la-Neuve, Belgium) with a fixed-energy proton beam using a Nirta [^18^F]fluoride XL target.

Reaction progress in the radioactive experiments was monitored by radio-thin-layer chromatography (radio-TLC) on Polygram^®^ SIL G/UV254 precoated plates (Macherey-Nagel, Düren, Germany) using ethyl acetate/*n*-hexane (1/1, *v*/*v*) as the eluent. The plates were exposed to storage phosphor screens (BAS-IP MS 2025, FUJIFILM Co., Tokyo, Japan) and recorded using the Amersham Typhoon RGB Biomolecular Imager (GE Healthcare Life Sciences, Marlborough, MA, USA). Images were quantified with the ImageQuant TL8.1 software (GE Healthcare Life Sciences).

Analytical chromatographic separations were performed on a JASCO LC-2000 system, incorporating a PU-2080*Plus* pump, AS-2055*Plus* auto injector (100 μL sample loop), and a UV-4075 detector (JASCO Deutschland GmbH, Pfungstadt, Germany) coupled with a gamma radioactivity HPLC detector (Gabi Star, Elysia-raytest GmbH, Straubenhardt, Germany). Data analysis was performed with the Galaxie chromatography software (Version 1.10.0.5590; Agilent Technologies, Santa Clara, CA, USA). Chromatographic analyses were performed on a Reprosil-Pur 120 C18-AQ column (radioactive experiments: 150 × 3.0 mm, 3 µm; non-radioactive experiments: 250 × 4.6 mm, 5 µm; Dr. Maisch HPLC GmbH, Ammerbuch, Germany) using MeCN/20 mM NH_4_OAc (aq., pH 6.8) as the eluent system. The flow rates were 0.4 mL·min^−1^ for radioactive and 1.0 mL·min^−1^ for non-radioactive experiments. Eluent A consisted of 10% MeCN in 20 mM NH_4_OAc (aq.), and eluent B of 90% MeCN in 20 mM NH_4_OAc (aq.). The gradients were as follows for [^18^F]**6**: 0–2 min, 100% A; 2–14 min, 0–100% B; 14–17 min, 100% B; 17–18 min, 100–0% B; 18–20 min, 100% A and for **6**: 0–20 min, 45% A; 20–21 min, 0–100% B; 21–24 min, 100% B; 24–25 min, 100–45% B; and 25–30 min, 45% A. The ammonium acetate concentration (20 mM NH_4_OAc) refers to the concentration in the aqueous component of the eluent mixture.

The anion exchange cartridges Sep-Pak^®^ Accell Plus QMA carbonate light (46 mg, Waters GmbH, Eschborn, Germany) were preconditioned with 6 mL of a 0.5 M aqueous NaHCO_3_ solution followed by 10 mL water to ensure standardized conditions. The Sep-Pak^®^ C18 Plus light cartridges (130 mg, Waters GmbH, Eschborn, Germany) were preconditioned with 10 mL EtOH followed by 10 mL water.

#### 4.2.2. Manual Radiosynthesis

No-carrier-added [^18^F]fluoride in 0.5 mL water was trapped on a Sep-Pak^®^ Accell Plus QMA carbonate light cartridge. The activity was eluted into a 4 mL V-vial using a mixture of 700 µL MeCN, 150 µL water and an aqueous solution of Et_4_NHCO_3_ (200 µL of 0.075 M, 15 µmol). The aqueous [^18^F]fluoride was azeotropically dried under vacuum and nitrogen flow within 7–10 min using a single-mode microwave (75 W, at 50–60 °C, power cycling mode, PETWave from CEM GmbH, Kamp-Lintford, Germany). Two aliquots of MeCN (2 × 1.0 mL) were added during the drying procedure and the final complex was dissolved in 500 µL of the respective labeling anhydrous solvent (DMSO, DMF, DMI or MeCN) ready for radiolabeling. Thereafter, a solution of 1.0–2.0 mg (2.4–4.8 µmol) of precursor in 300 µL of the respective solvent was added, and the ^18^F-labeling was performed at different temperatures (100–200 °C). To analyze the reaction mixture and to determine RCCs, samples were taken for radio-TLC and radio-HPLC at different time points (5, 10, 15 and 20 min).

#### 4.2.3. Automated Radiosynthesis

The automated radiosynthesis of [^18^F]**6** was performed using a TRACERlab FX2 N synthesizer (GE Healthcare, Waukesha, WI, USA) equipped with a Laboport vacuum pump N810.3FT.18 (KNF Neuburger GmbH, Freiburg, Germany), a BlueShadow UV detector 10D (KNAUER GmbH, Berlin, Germany) and the TRACERlab FX Software (Version 2.2.2).

**Figure 6:**
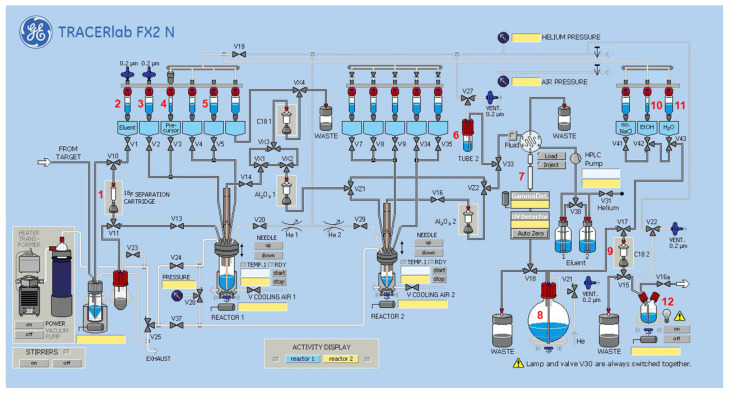
Scheme of the synthesis module TRACERlab FX2 N for the radiosynthesis of [^18^F]**6**. (1) Sep-Pak^®^ Accell Plus QMA carbonate light, (2) Et_4_NHCO_3_ in MeCN/water (15 µmol in 350 µL water and 700 µL MeCN), (3) 1.5 mL MeCN, (4) precursor **AG-881** (1.0 mg,2.4 µmol, in 1.0 mL anhydrous DMSO), (5) 3.0 mL water and 1.0 mL MeCN, (6) injection vial, (7) Reprosil-Pur 120 C18-AQ, 55% MeCN in 20 mM NH_4_OAc (aq., *v*/*v*), 4.0 mL/min, (8) 30 mL water, (9) Sep-Pak^®^ C18 Plus light, (10) 2.0 mL water, (11) 1.3 mL EtOH, and (12) product vial.

[^18^F]Fluoride (~25 GBq) was trapped on a Sep-Pak^®^ Accell Plus QMA carbonate light cartridge (Figure 6, entry **1**) and eluted into the reactor with a mixture of 700 µL MeCN, 150 µL water and an aqueous solution of Et_4_NHCO_3_ (200 µL of 0.075 M, 15 µmol, entry **2**). The mixture was azeotropically dried for 4 min at 75 °C and then 1.5 mL anhydrous MeCN (entry **3**) was added for a further 3 min at 85 °C. The reactor was then cooled to 60 °C and 1.0–1.4 mg (2.4–3.4 µmol) of the precursor (**AG-881**) dissolved in 1.0 mL anhydrous DMSO (entry **4**) was added. For radiolabeling, the reaction mixture was stirred at 160 °C for 20 min. After cooling, the reaction mixture was diluted with 3.0 mL H_2_O and 1.0 mL MeCN (entry **5**) and transferred into the injection vial (entry **6**). Semi-preparative HPLC was performed on a Reprosil-Pur 120 C18-AQ column (250 × 10 mm, 10 µm; Dr. Maisch HPLC GmbH, Germany) using 55% MeCN in 20 mM NH_4_OAc (aq., *v*/*v*) as the eluent at a flow rate of 4.0 mL·min^−1^ (entry **7**). [^18^F]**6** was collected in the dilution vessel (entry **8**) pre-loaded with 30 mL H_2_O. Final purification was performed by passing the solution through a Sep-Pak^®^ C18 Plus light cartridge (entry **9**), followed by washing with 2.0 mL water (entry **10**) and an elution of [^18^F]**6** with 1.3 mL EtOH (entry **11**) into the product vial (entry **12**). The ethanolic solution was transferred out of the hot cell and the solvent was reduced under gentle argon stream at 70 °C to a final volume of 30–80 µL. Afterwards, the radiotracer was diluted in isotonic saline to obtain a final product containing 10% of EtOH (*v*/*v*) with an activity concentration of about 1 MBq/µL.

The molar activity was determined based on a calibration curve under isocratic HPLC conditions using a Reprosil-Pur 120 C18-AQ column (150 × 3.0 mm, 3 µm; Dr. Maisch HPLC GmbH, Germany) with 50% MeCN in 20 mM NH_4_OAc (aq., *v*/*v*) as the eluent at a flow rate of 0.4 mL·min^−1^. Chromatograms were recorded at 270 nm, corresponding to the maximum UV absorbance, and were used for quantification.

#### 4.2.4. In Vitro Stability Measurements of [^18^F]**6**

The in vitro stability of [^18^F]**6** was investigated by incubation of small tracer amounts (~10 MBq) at 37 °C in isotonic saline (pH 6.5), phosphate-buffered saline (pH 7.4) and the cell culture medium RPMI 1640 containing 10% FBS (500 µL of each medium). From saline and PBS incubations, aliquots were taken after 1, 2 and 3 h and were directly analyzed by radio-HPLC. For the preparation of the RPMI samples, proteins were precipitated by the addition of 1 mL ice-cold acetonitrile to each of the four incubations (1, 2, 3 and 4 h). The samples were then vortexed for 2 min and centrifuged for 5 min at 10,000 rpm. From the supernatants, 100 µL was taken and analyzed by radio-HPLC. The recovery rate of this process was 98–100%, as determined by measuring aliquots of the supernatants as well as the precipitates in a γ-counter (PerkinElmer Wallac Wizard 1480 Gamma Counter, manufactured by WALLAC, Turku, Finland).

### 4.3. In Vitro Biology

#### 4.3.1. Cell Culture

Human U251 glioblastoma cells, stably transfected and overexpressing either wild-type IDH1 (IDH1^wt^-U251) or IDH1^R132H^ (IDH1^R132H^-U251), were obtained from Jaqueline Kessler and Dirk Vordermark (Department of Radiotherapy, Martin Luther University Halle-Wittenberg, Halle/Saale, Germany). Cells were grown in RPMI 1640 medium (10% FCS), supplemented with puromycin, to ensure the cultivation of transfected cells only.

#### 4.3.2. Mutant IDH1 Enzyme Assay for the Determination of Inhibitory Potency

Determination of the activity and inhibition of mutant IDH1 recombinant protein, IDH1^R132H^, is based on the reduction of α-KG acid to D-2-HG accompanied by a concomitant oxidation of NADPH to NADP. The amount of NADPH remaining at the end of the reaction is measured in a secondary diaphorase/resazurin reaction, in which the NADPH is consumed in a 1/1 molar ratio with the conversion of resazurin to the highly fluorescent resorufin. For the determination of the inhibitory potential of ligands, the IDH1^R132H^ Assay Kit (BPS-79376, BPS Bioscience, San Diego, CA, USA) was used. The enzyme activity assay was performed in a volume of 100 μL buffer (20 mM TRIS buffer (pH = 7.5), 150 mM NaCl, 10 mM MgCl_2_, 0.05% bovine serum albumin (BSA), and 4 mM β-mercaptoethanol) containing 0.5 ng/μL IDH1^R132H^ enzyme, 2 mM α-KG, and 12 μM NADPH. For inhibition assays, triplicate samples of the ligands in the concentration range from 10^−5^ M to 10^−10^ M were incubated with the enzyme for 30 min before the addition of α-KG and NADPH to initiate the reaction. The reaction ran for 60 min at room temperature and was terminated with the addition of 25 μL of detection buffer (36 μg/mL diaphorase and 30 mM resazurin) to 50 μL of the reaction solution. The conversion of resazurin to resorufin by diaphorase was measured fluorometrically at Ex544/Em590 (Synergy H1 microplate reader, BioTek, Winooski, VT, USA). The data were imported into GraphPad Prism 4.1 (GraphPad Inc., La Jolla, CA, USA), and the IC_50_ values were calculated with a standard dose–response curve fitting.

#### 4.3.3. In Vitro Cell Uptake

The IDH1^wt^-U251 and IDH1^R132H^-U251 cells were seeded at 400,000 cells/mL in a 24-well cell culture plate 1 day before the experiment. The medium was replaced by 400 μL/well, and 5 μL of a 100 x stock solution of **AG-881** and **BAY1436032** was added. The experiment was started with the addition of 100 μL of [^18^F]**6** (0.382 ± 0.054 MBq/mL; 4.8 and 6.4 nM) diluted in cell culture medium per well, and the well plates were incubated in a humidified-air atmosphere incubator containing 5% CO_2_ at 37 °C for various times. Incubation was stopped by aspiration of the supernatant and washing the cell layers twice with prechilled PBS (500 μL/well). Cell surface-bound activity was released by the addition of an acid–glycine buffer (0.2 M glycine, 0.15 M NaCl, pH 3; 500 μL/well) and incubation at room temperature for 10 min. The supernatant was collected and pooled with the supernatant obtained by subsequent washing with PBS (500 μL/well). Finally, the cells were lysed (0.1 M NaOH + 1% SDS; 500 μL/well; 37 °C, 30 min). Activities in the acidic wash and lysis samples, along with aliquots of the radioligand solution, were measured in a γ-counter (Wallac 2480 Wizard, PerkinElmer, Waltham, MA, USA). Cells cultured in an additional well plate and treated as above, except for the addition of radioligand, were used as a control and to determine the protein concentration per well by a BCA assay (Pierce, #23227). The concentrations of surface-bound and internalized activities per well were calculated as a percentage of the applied dose per well and normalized to the protein concentration per well (% AD/μg protein). Two experiments were performed in triplicate.

## 5. Conclusions

The findings emphasize the importance of optimizing both structural and physicochemical properties, such as substituent positioning and steric compatibility, to improve target-specific potency and binding. Further chemical modifications are necessary to develop aminotriazine-based PET tracers with enhanced target engagement. Future efforts may focus on fine-tuning pyridyl substituents and side-chain architecture to balance physicochemical properties, enzymatic inhibition, radiolabeling feasibility and in vitro performance.

## Figures and Tables

**Table 1:** Overview of synthesized derivatives and their potency towards IDH1^R132H^.

Compound	IC_50_ IDH1^R132H^ (nM)
**AG-881**	18.0 ± 1.9 (*n* = 3)
**6**	405 ± 95 (*n* = 3)
**7**	944 (*n* = 1)
**8**	>10,000 (*n* = 2)
**9**	991 (*n* = 1)
**10**	>10,000 (*n* = 3)
**11**	1100 (*n* = 1)

## Data Availability

The original contributions presented in this study are included in the article/Supplementary Materials. Further inquiries can be directed to the corresponding authors.

## Supplementary Materials

The following supporting information can be downloaded at: https://www.mdpi.com/article/10.3390/ph19050660/s1.

## Abbreviations

The following abbreviations are used in this manuscript:

IDH	Isocitrate dehydrogenase
PET	Positron emission tomography
SAR	Structure–activity relationship
SPE	Solid phase extraction
RCC	Radiochemical conversion
EOB	End of bombardment
EOS	End of synthesis
