# Supplementary material for: Fluorinated Derivatives of AG-881 for Positron Emission Tomography Detection of Mutated Isocitrate Dehydrogenase 1
Source: Pharmaceuticals (Basel). 2026 Apr 23;19(5):660. doi: 10.3390/ph19050660 (PMC13210206; doi:10.3390/ph19050660)
Supplement: Supplementary file 1 [file pharmaceuticals-19-00660-s001.zip › pharmaceuticals-4201881-supplementary.pdf]

Supplementary Information: Fluorinated derivatives of AG-881 for Positron Emission Tomography detection of mutated Isocitrate Dehydrogenase 1

Thu Hang Lai [1,†], Sladjana Dukić-Stefanović [2,†], Winnie Deuther-Conrad [2], Aurélie Maisonial-Besset [3], Rodrigo Teodoro [2], Magali Toussaint [2,*] and Barbara Wenzel [2,*]

1 Department of Research and Development, ROTOP Pharmaka GmbH, 01328 Dresden, Germany; publications@rotop-pharmaka.de
2 Department of Experimental Neurooncological Radiopharmacy, Institute of Radiopharmaceutical Cancer Research, Research Site Leipzig, Helmholtz-Zentrum Dresden-Rossendorf (HZDR), 04318 Leipzig, Germany; s.dukic-stefanovic@hzdr.de (S.D.-S.); w.deuther-conrad@hzdr.de (W.D.-C.); rodrigo.teodoro@lantheus.com (R.T.)
3 UMR INSERM 1240, Molecular Imaging and Theranostic Strategies, University of Clermont Auvergne, 63000 Clermont-Ferrand, France; aurelie.maisonial@uca.fr
* Correspondence: m.toussaint@hzdr.de (M.T.); b.wenzel@hzdr.de (B.W.)
† These authors contributed equally to this work.

Chemical Synthesis 2

Figure S1. NMR spectra of 6-chloro-*N*²,*N*⁴-bis((*R*)-1,1,1-trifluoropropan-2-yl)-1,3,5-triazine-2,4-diamine (**2**). 2

Figure S2. NMR spectra of 6-chloro-*N*²,*N*⁴-bis((*R*)-1,1,1-trifluorobutan-2-yl)-1,3,5-triazine-2,4-diamine (**3**). 5

Figure S3. NMR spectra of (*R*)-4,6-dichloro-*N*-(1,1,1-trifluoropropan-2-yl)-1,3,5-triazin-2-amine **4**.. 7

Figure S4. NMR spectra of (*R*)-6-chloro-*N*²-(3,3-difluorocyclobutyl)-*N*⁴-(1,1,1-trifluoropropan-2-yl)-1,3,5-triazine-2,4-diamine (**5**) 10

Figure S5. NMR spectra of **AG-881**. 11

Figure S6. NMR spectra of 6-(5-chloro-6-fluoropyridin-3-yl)-*N*²,*N*⁴-bis((*R*)-1,1,1-trifluoropropan-2-yl)-1,3,5-triazine-2,4-diamine (**6**) 13

Figure S7. NMR spectra of 6-(6-fluoropyridin-2-yl)-*N*²,*N*⁴-bis((*R*)-1,1,1-trifluoropropan-2-yl)-1,3,5-triazine-2,4-diamine (**7**). 16

Figure S8. NMR spectra of 6-(6-fluoropyridin-3-yl)-*N*²,*N*⁴-bis((*R*)-1,1,1-trifluoropropan-2-yl)-1,3,5-triazine-2,4-diamine (**8**). 17

Figure S9. NMR spectra of 6-(5-chloro-6-fluoropyridin-3-yl)-*N*²,*N*⁴-bis((*R*)-1,1,1-trifluorobutan-2-yl)-1,3,5-triazine-2,4-diamine (**9**) 20

Figure S10. NMR spectra of 6-(6-fluoropyridin-3-yl)-*N*²,*N*⁴-bis((*R*)-1,1,1-trifluorobutan-2-yl)-1,3,5-triazine-2,4-diamine (**10**). 23

Figure S11. NMR spectra of (*R*)-6-(5-chloro-6-fluoropyridin-3-yl)-*N*²-(3,3-difluorocyclobutyl)-*N*⁴-(1,1,1-trifluoropropan-2-yl)-1,3,5-triazine-2,4-diamine (**11**). 26

Radiochemistry 29

Table S1. Investigated reaction parameters for the radiolabeling of the precursor **AG-881** to produce [^{18}F]**6** 29

Figure S12. Radio-HPLC chromatograms of stability tests 30

Chemical Synthesis

Figure S1. NMR spectra of 6-chloro-*N*[2],*N*[4]-bis((*R*)-1,1,1-trifluoropropan-2-yl)-1,3,5-triazine-2,4-diamine (**2**).

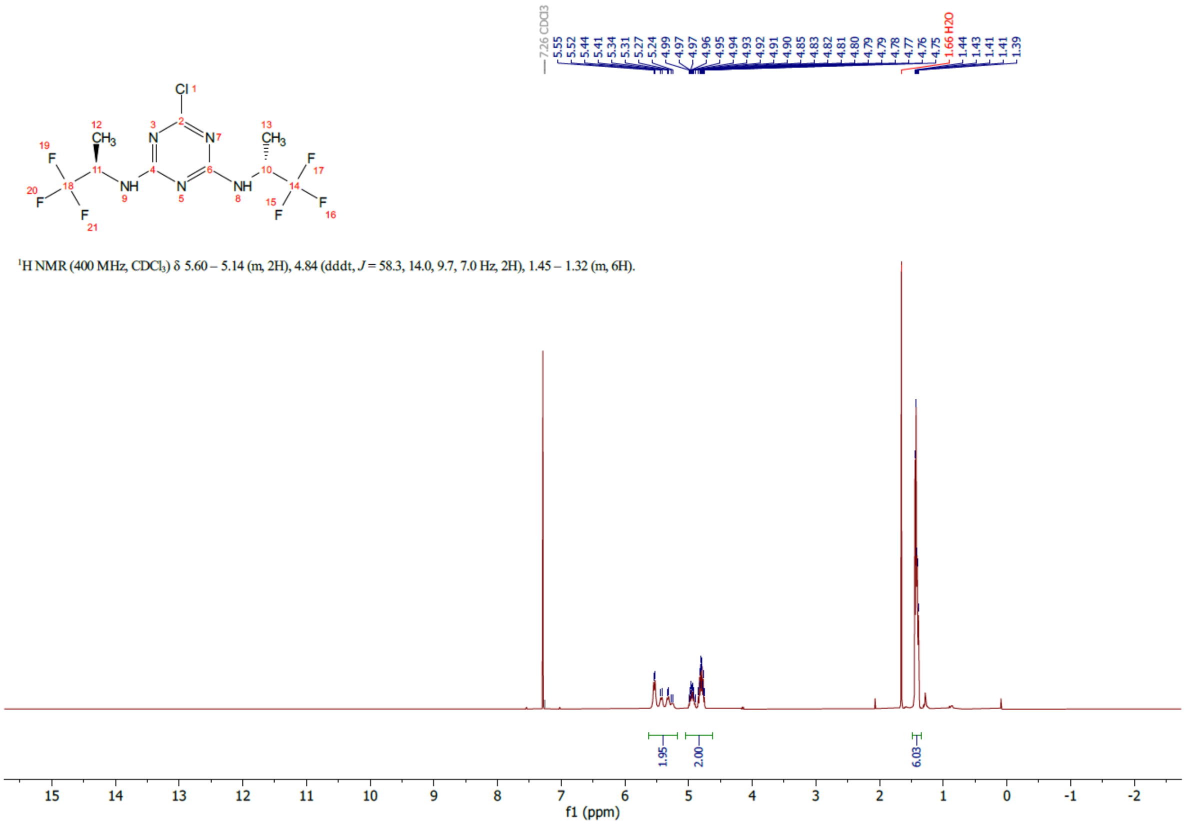

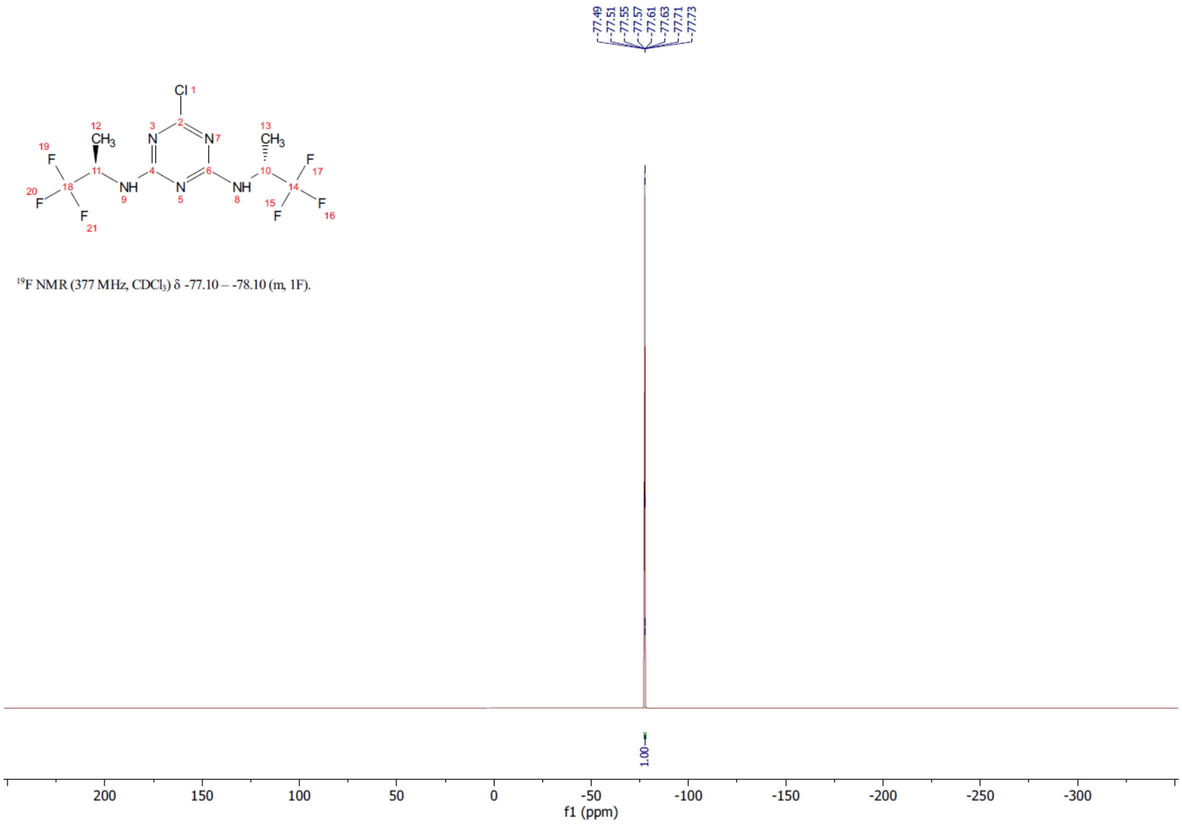

^{19}F NMR (377 MHz, $CDCl_3$) δ -77.10 – -78.10 (m, 1F).

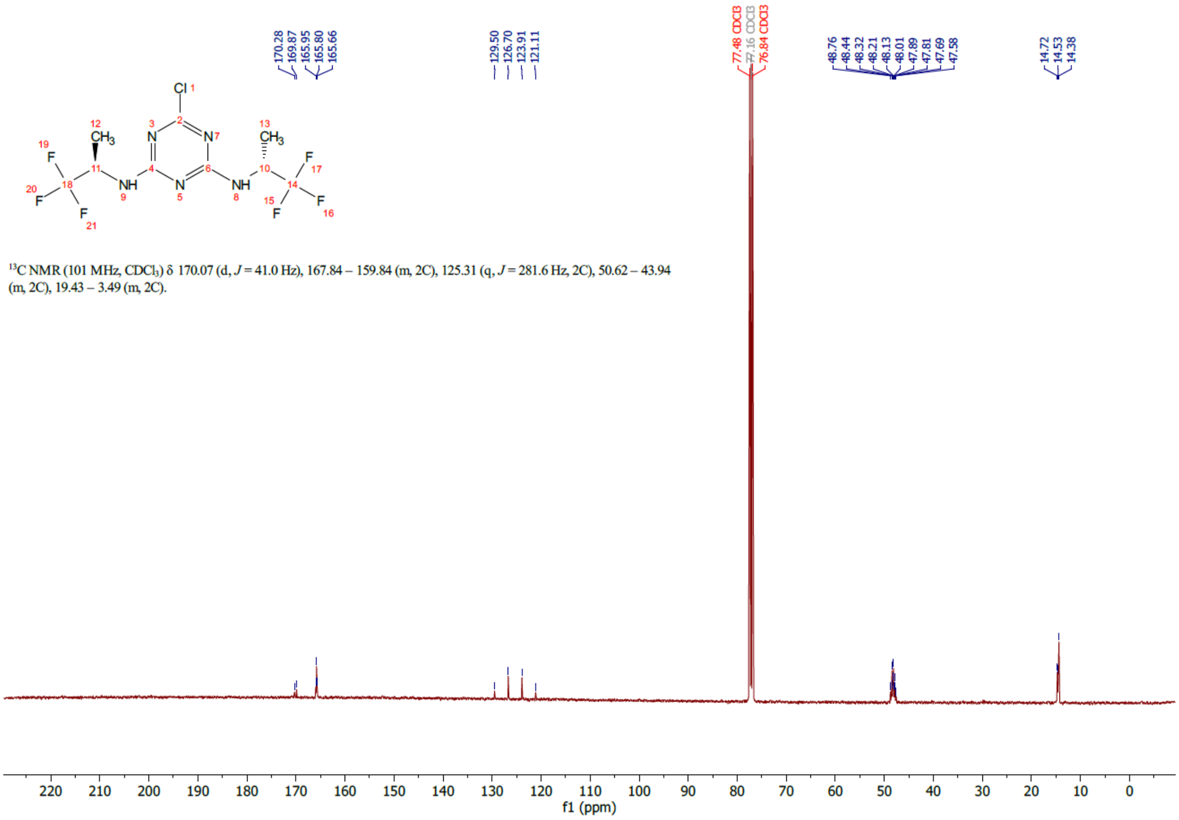

^{13}C NMR (101 MHz, $CDCl_3$) δ 170.07 (d, J = 41.0 Hz), 167.84 – 159.84 (m, 2C), 125.31 (q, J = 281.6 Hz, 2C), 50.62 – 43.94 (m, 2C), 19.43 – 3.49 (m, 2C).

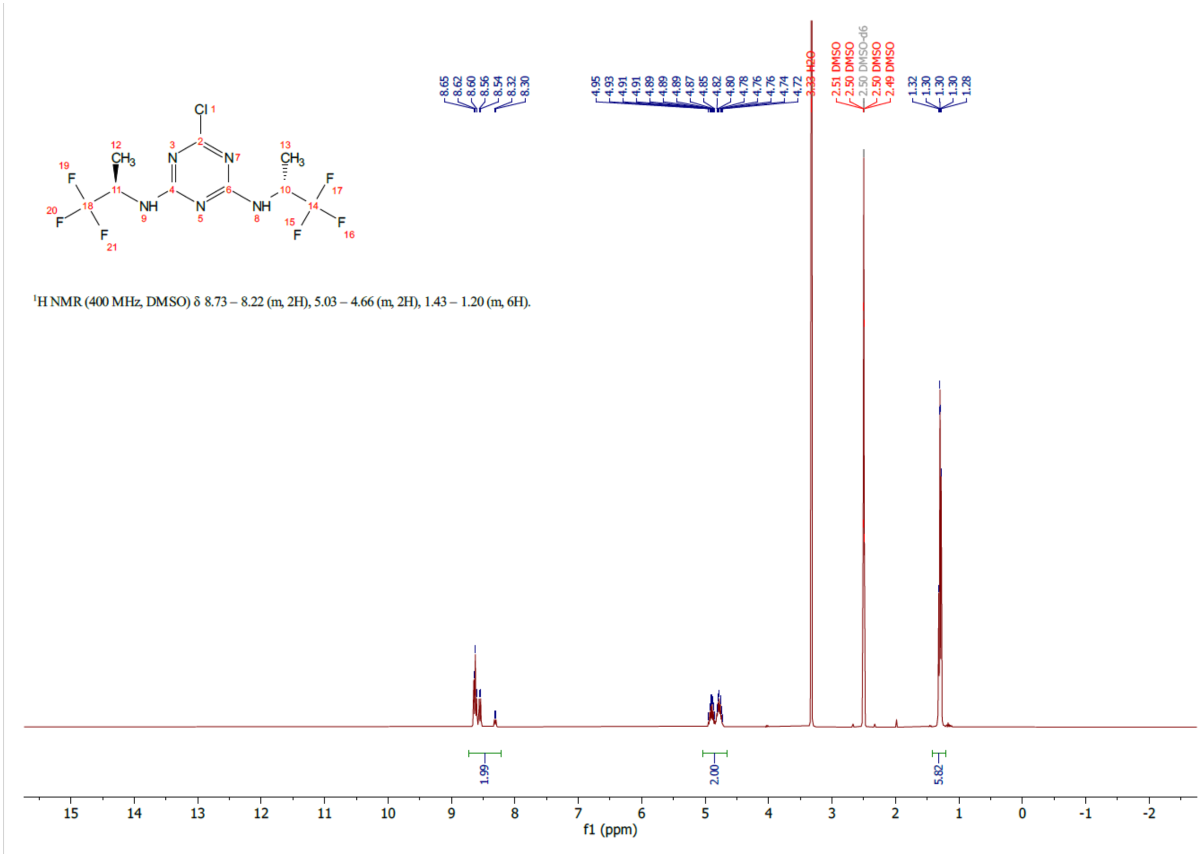

^{1}H NMR (400 MHz, DMSO) δ 8.73 – 8.22 (m, 2H), 5.03 – 4.66 (m, 2H), 1.43 – 1.20 (m, 6H).

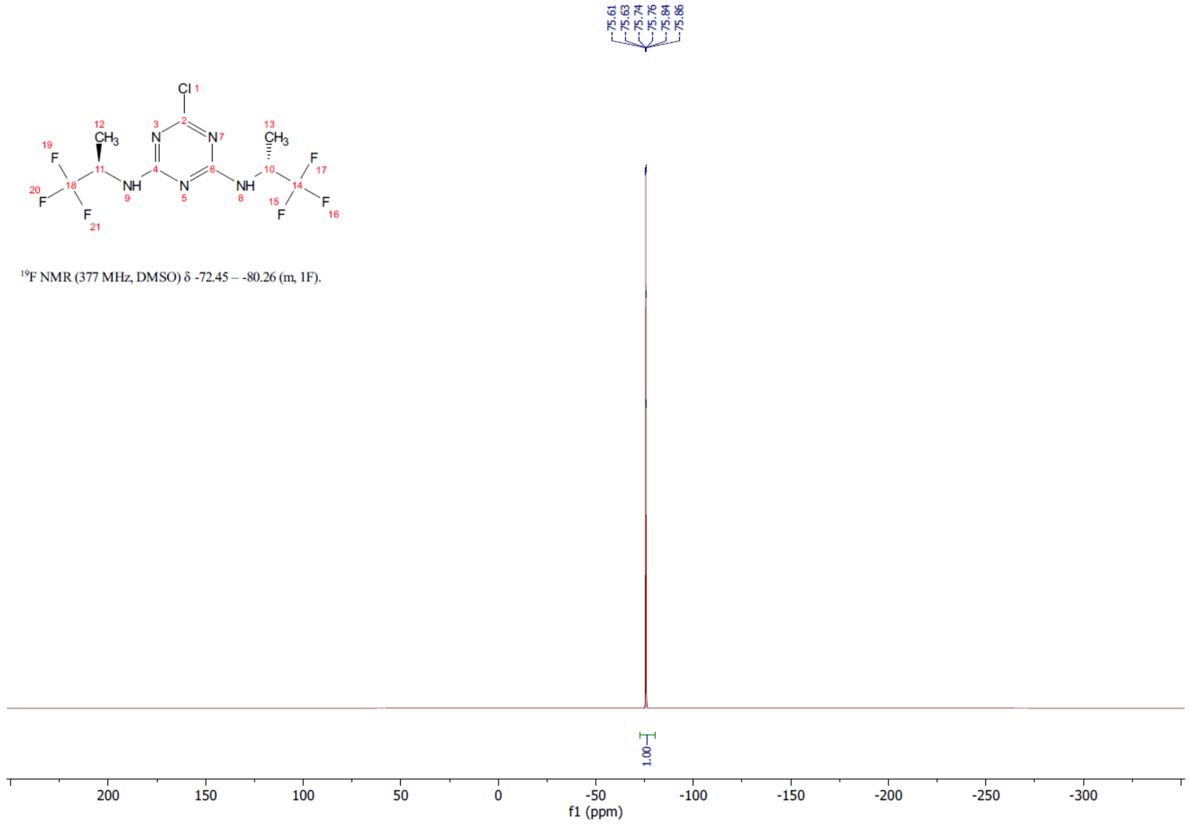

^{19}F NMR (377 MHz, DMSO) δ -72.45 – -80.26 (m, 1F).

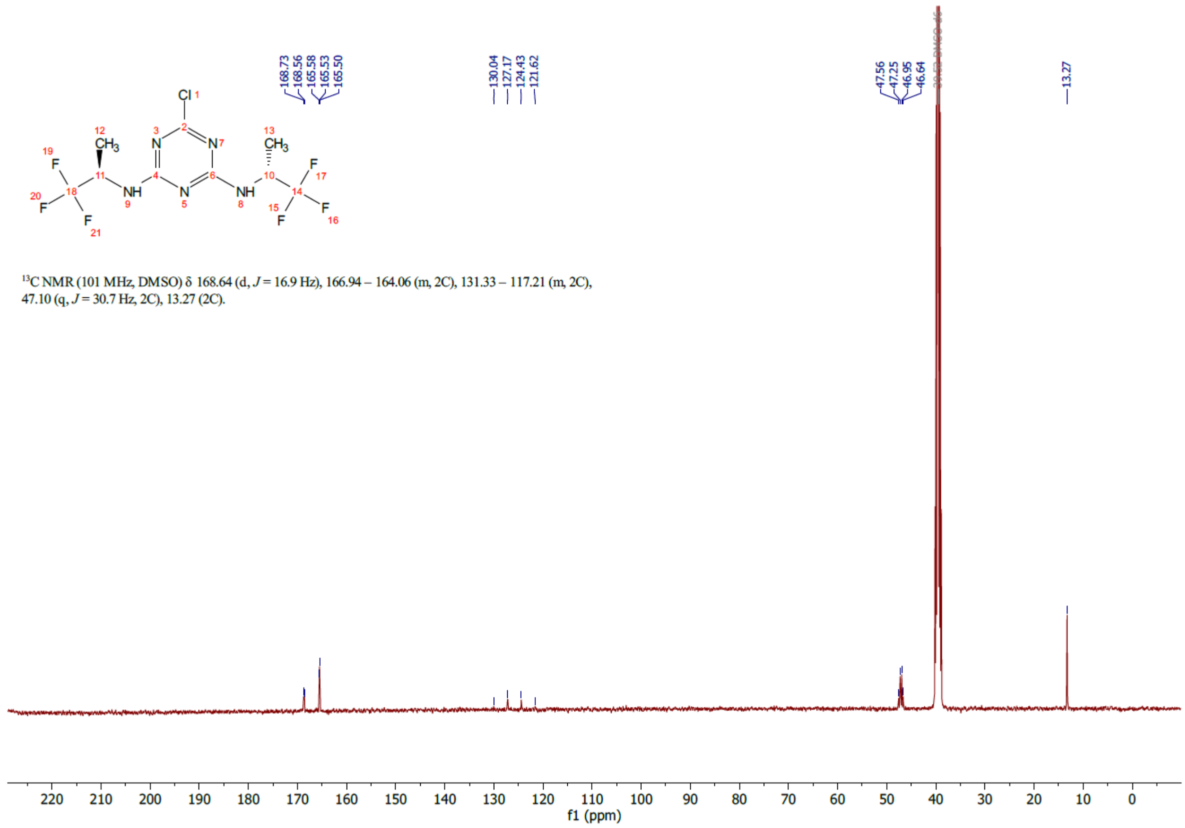

Figure S2. NMR spectra of 6-chloro-N^2,N^4-bis((*R*)-1,1,1-trifluorobutan-2-yl)-1,3,5-triazine-2,4-diamine (**3**).

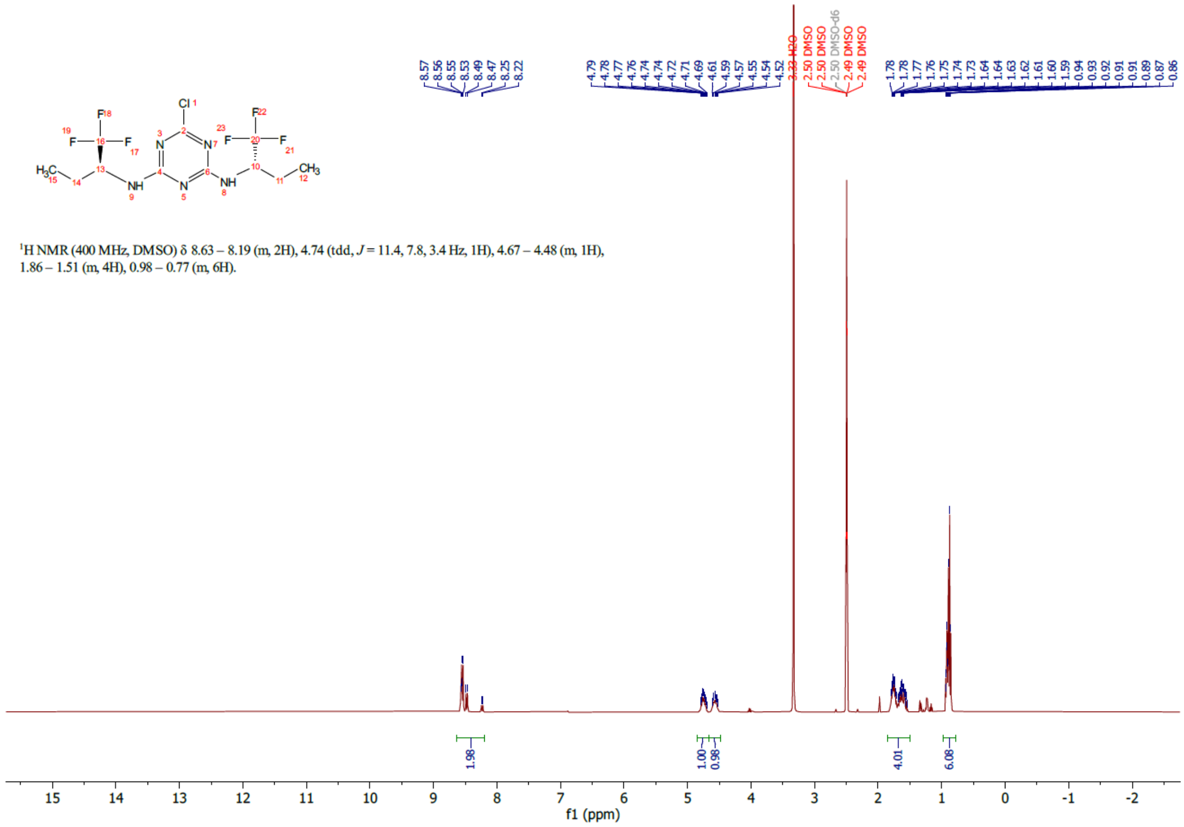

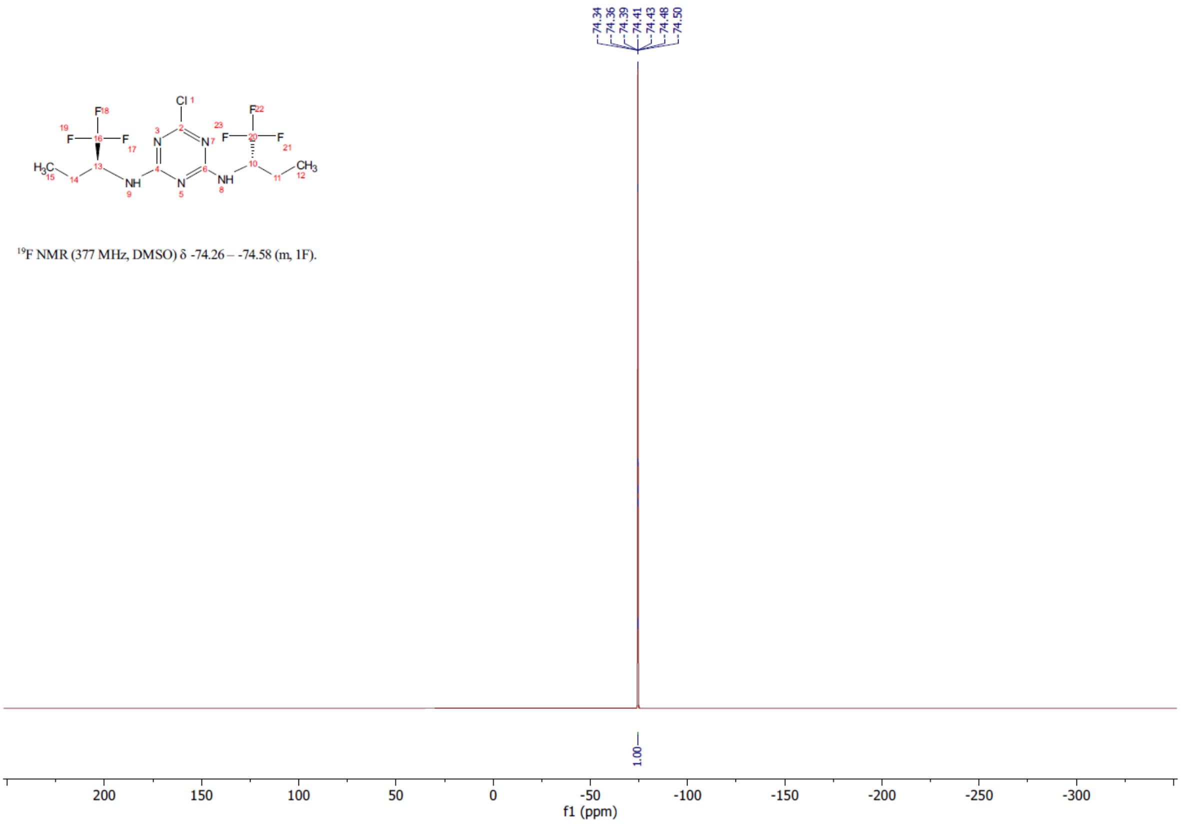

^{19}F NMR (377 MHz, DMSO) δ -74.26 – -74.58 (m, 1F).

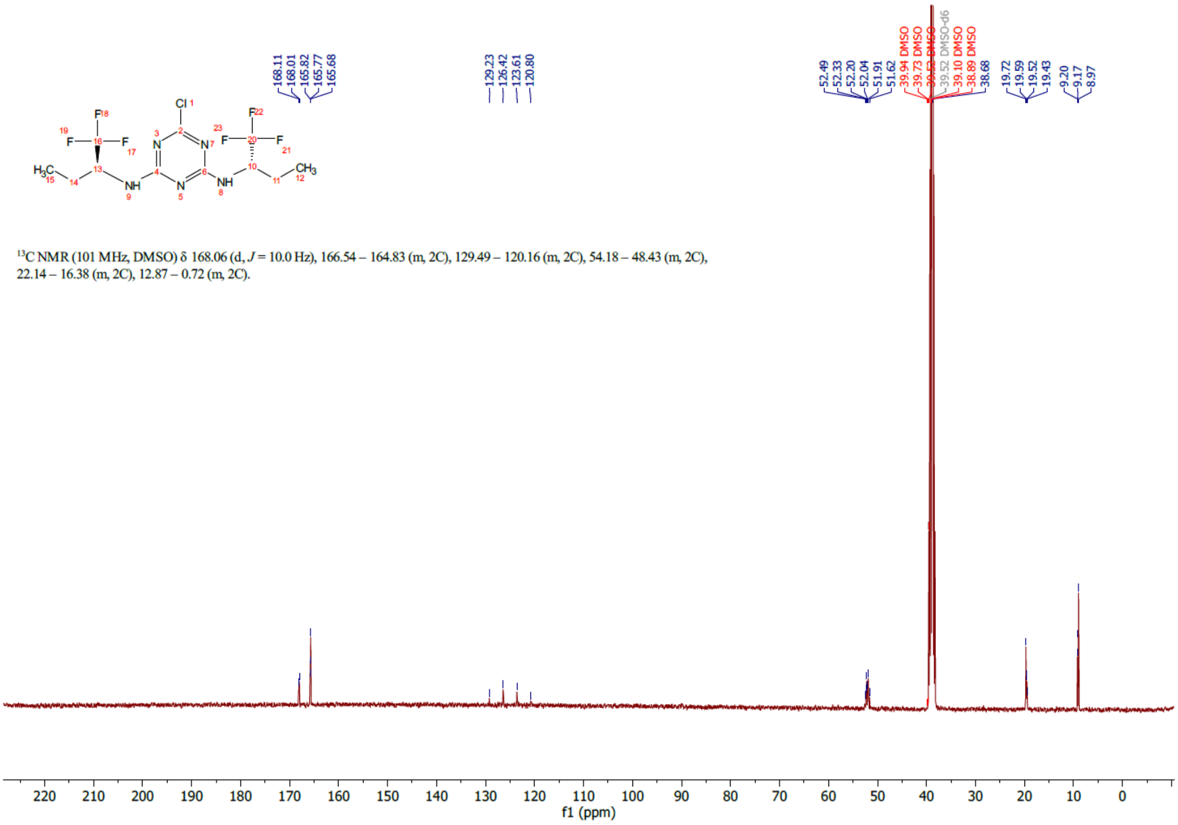

^{13}C NMR (101 MHz, DMSO) δ 168.06 (d, J = 10.0 Hz), 166.54 – 164.83 (m, 2C), 129.49 – 120.16 (m, 2C), 54.18 – 48.43 (m, 2C), 22.14 – 16.38 (m, 2C), 12.87 – 0.72 (m, 2C).

Figure S3. NMR spectra of (*R*)-4,6-dichloro-*N*-(1,1,1-trifluoropropan-2-yl)-1,3,5-triazin-2-amine **4**

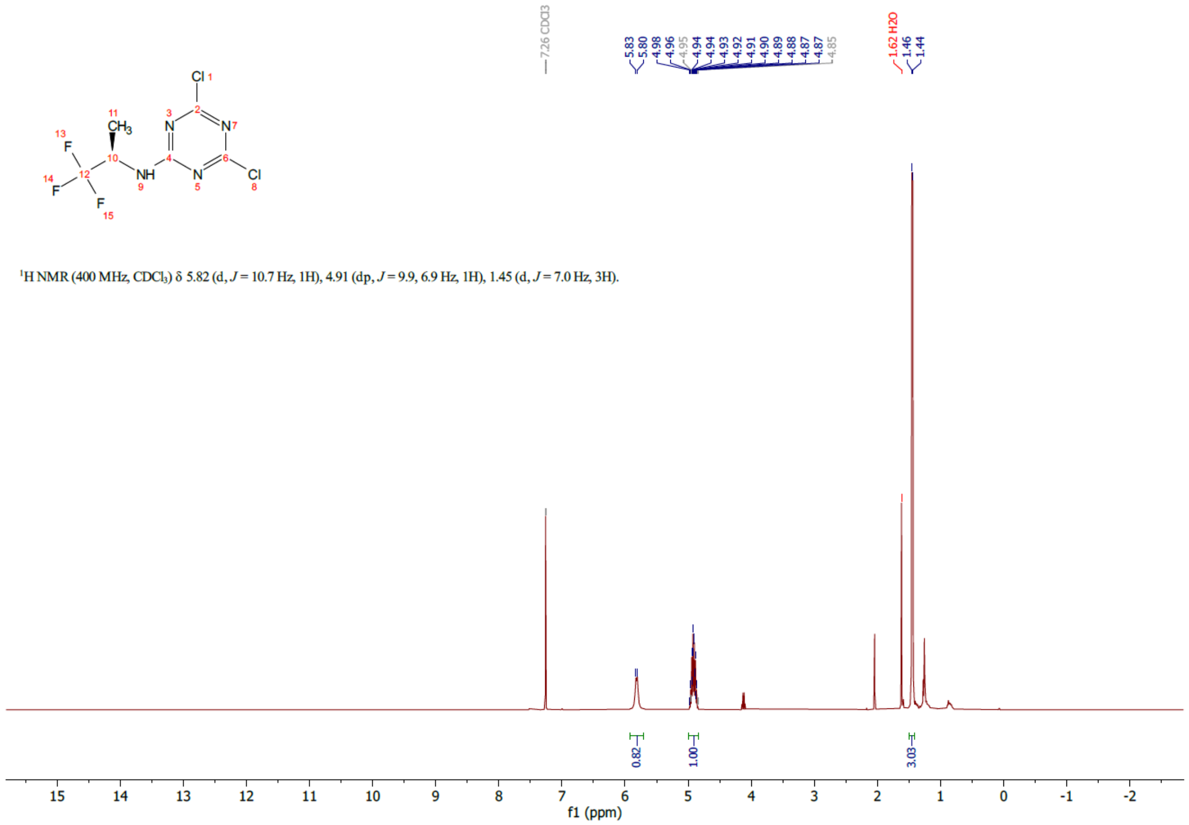

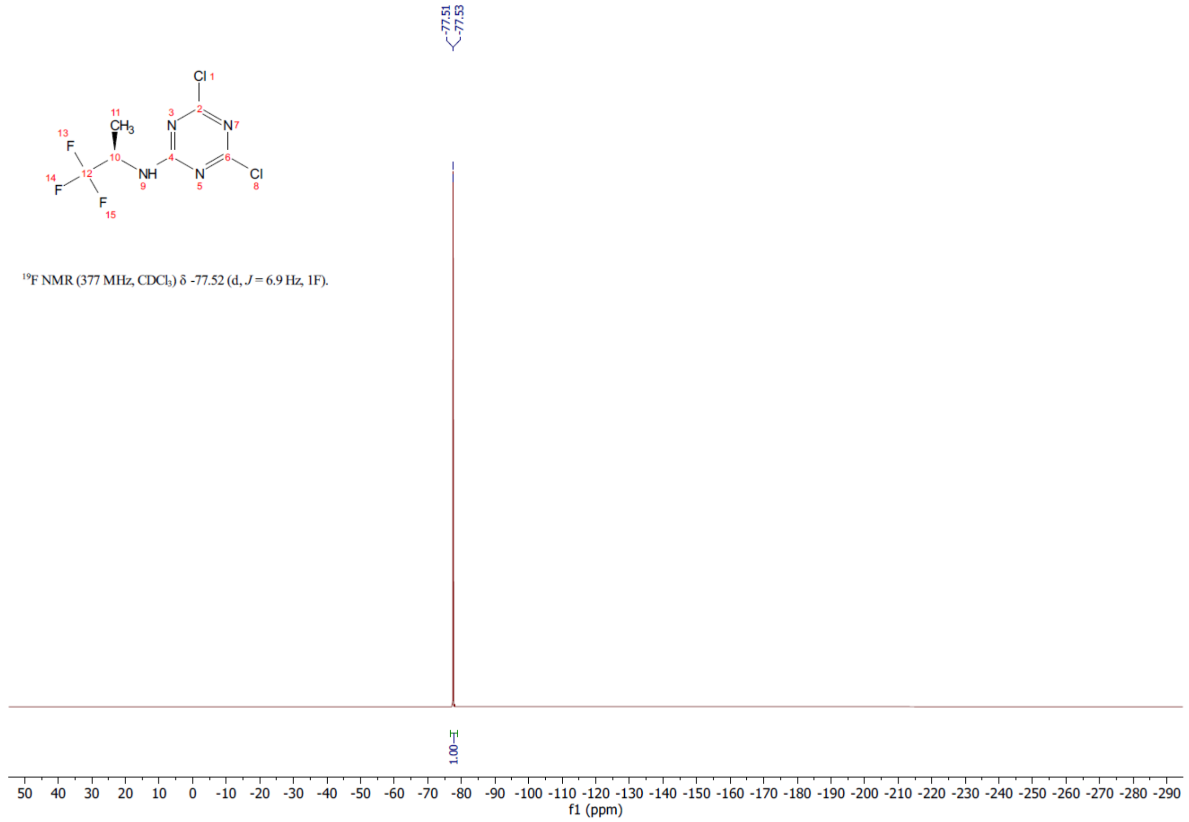

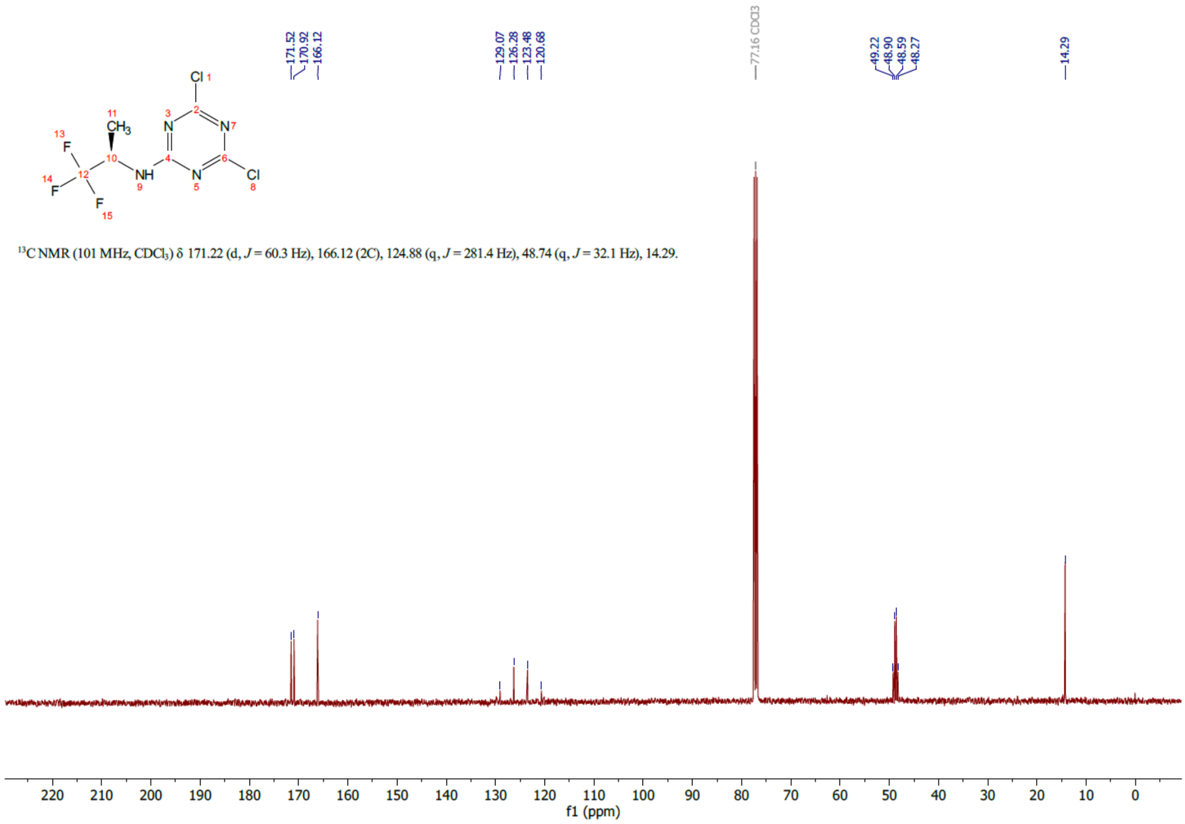

^{13}C NMR (101 MHz, $CDCl_3$) δ 171.22 (d, J = 60.3 Hz), 166.12 (2C), 124.88 (q, J = 281.4 Hz), 48.74 (q, J = 32.1 Hz), 14.29.

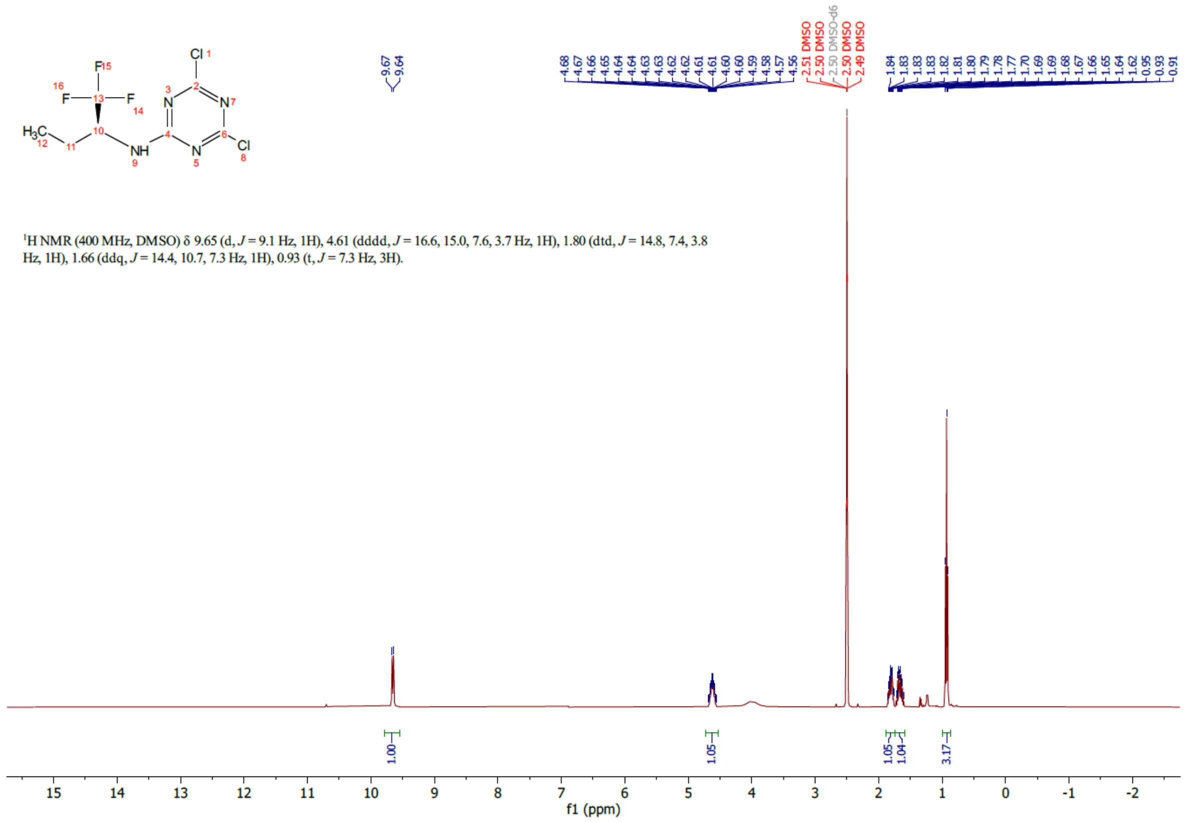

^{1}H NMR (400 MHz, DMSO) δ 9.65 (d, J = 9.1 Hz, 1H), 4.61 (dddd, J = 16.6, 15.0, 7.6, 3.7 Hz, 1H), 1.80 (dtd, J = 14.8, 7.4, 3.8 Hz, 1H), 1.66 (ddq, J = 14.4, 10.7, 7.3 Hz, 1H), 0.93 (t, J = 7.3 Hz, 3H).

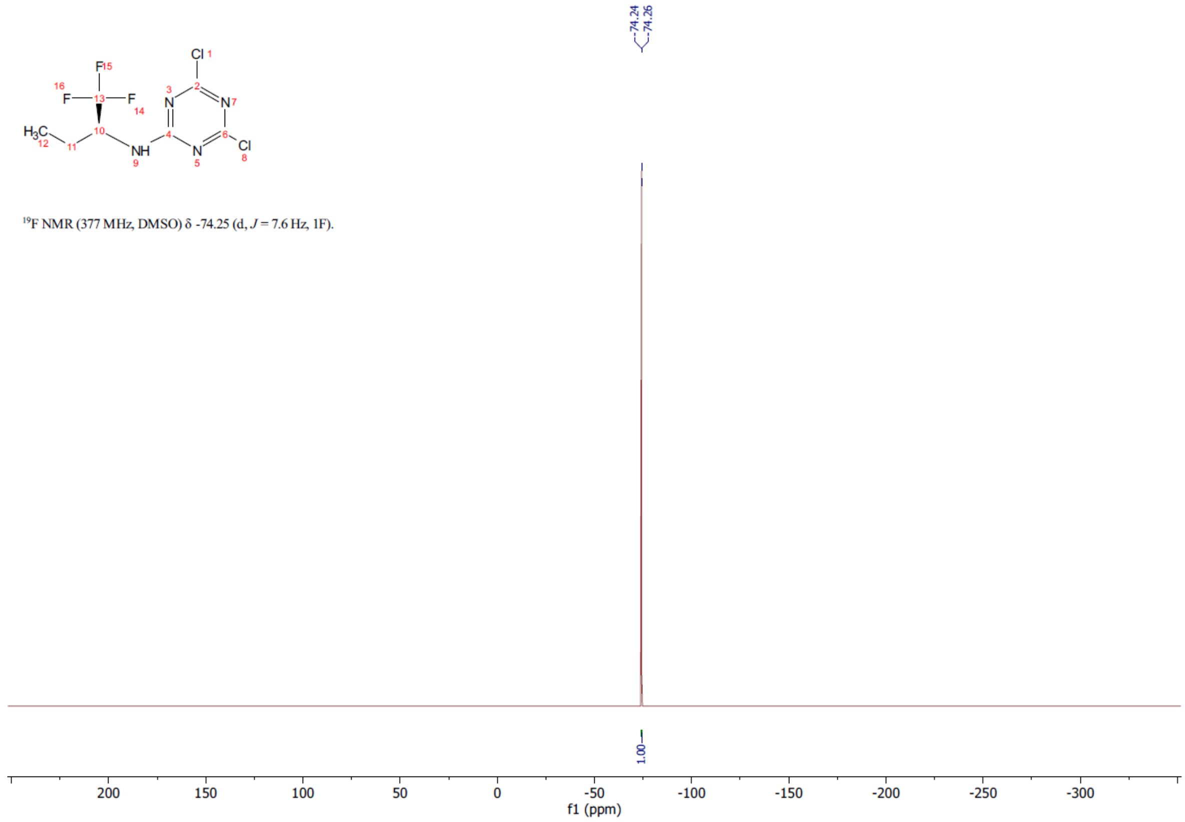

^{19}F NMR (377 MHz, DMSO) δ -74.25 (d, J = 7.6 Hz, 1F).

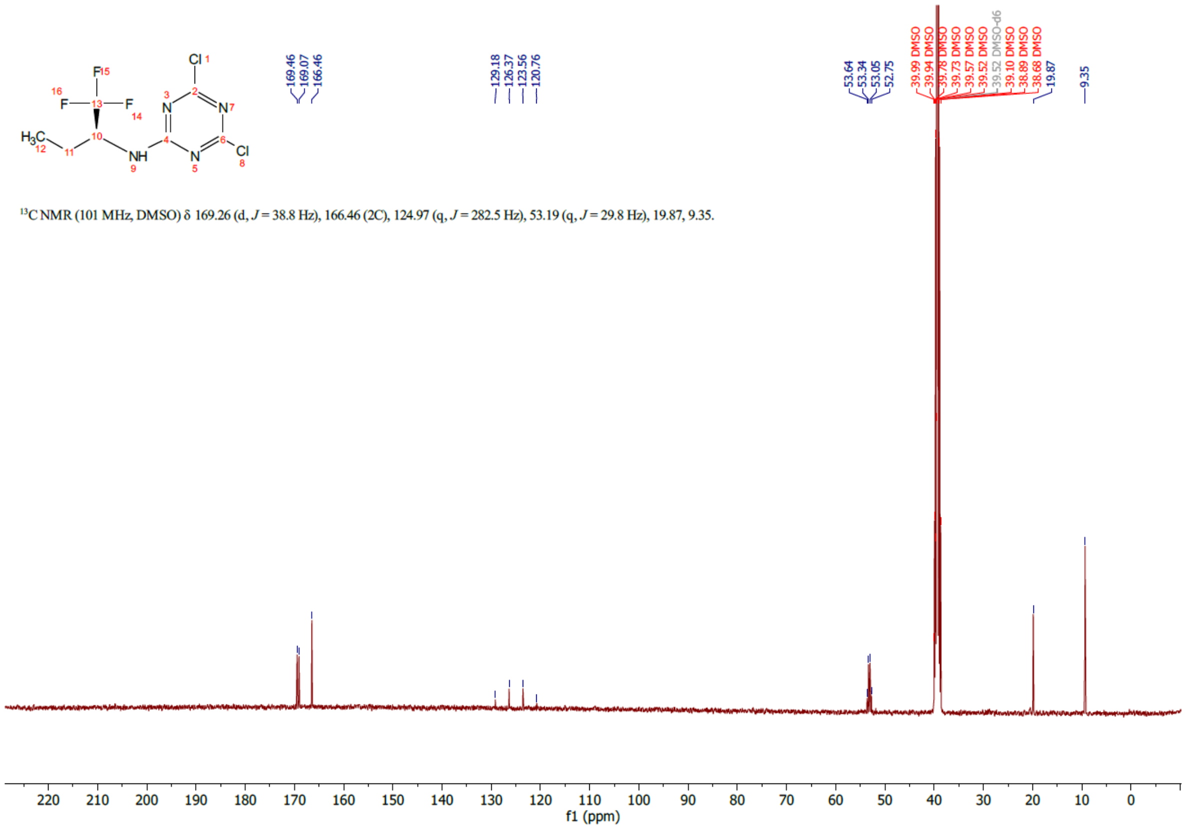

^{13}C NMR (101 MHz, DMSO) δ 169.26 (d, J = 38.8 Hz), 166.46 (2C), 124.97 (q, J = 282.5 Hz), 53.19 (q, J = 29.8 Hz), 19.87, 9.35.

Figure S4. NMR spectra of (*R*)-6-chloro-*N*[2]-(3,3-difluorocyclobutyl)-*N*[4]-(1,1,1-trifluoropropan-2-yl)-1,3,5-triazine-2,4-diamine (**5**).

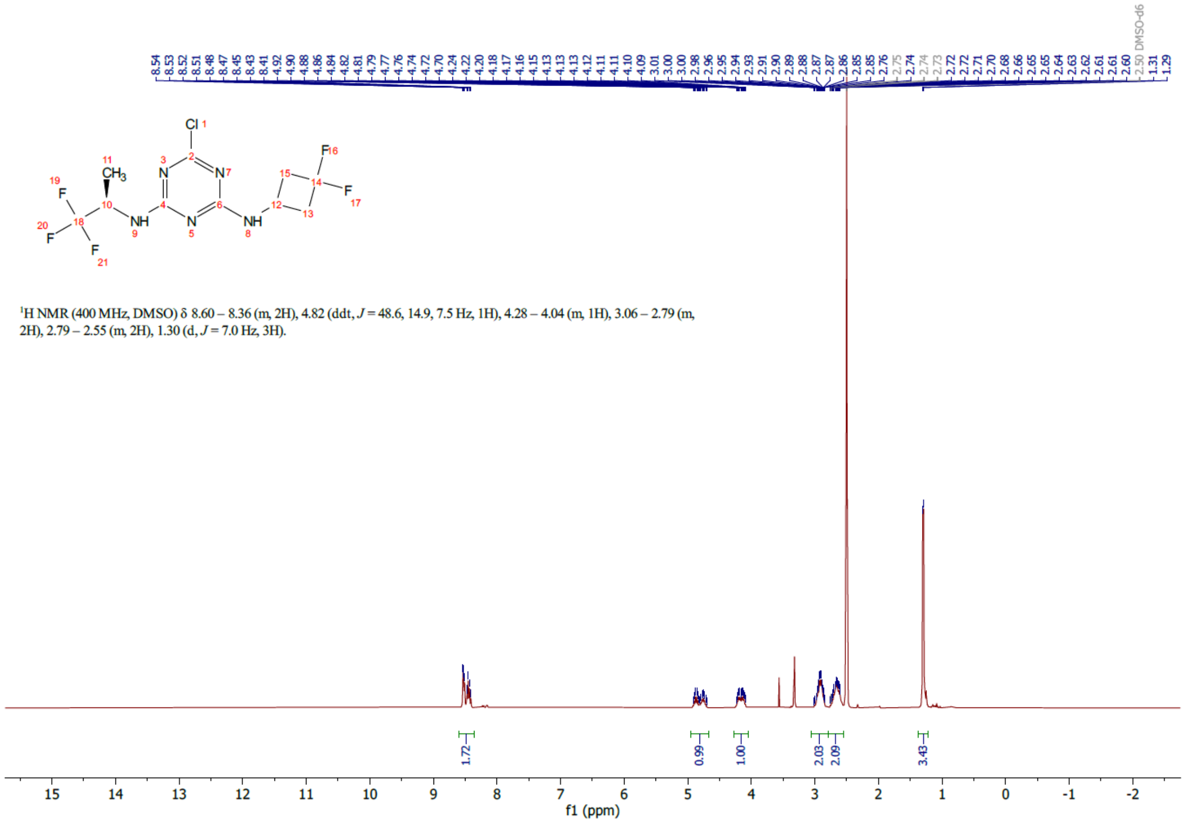

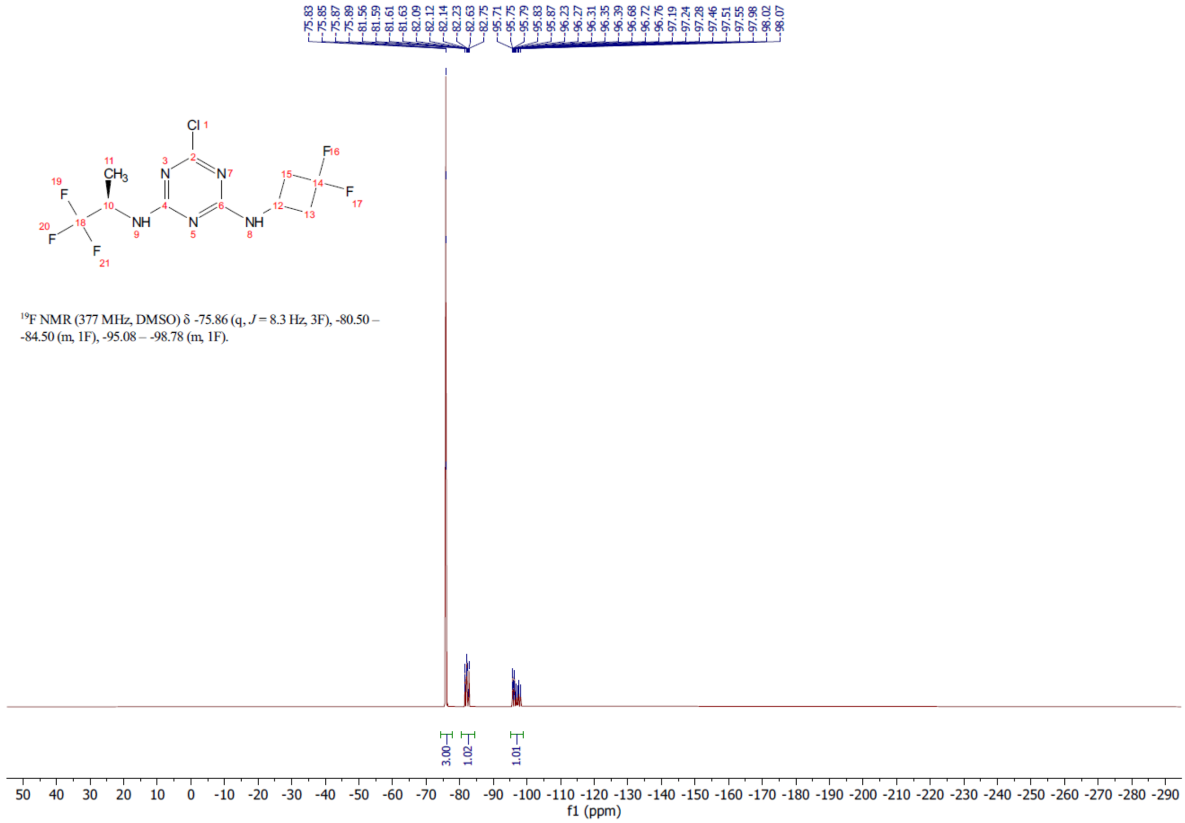

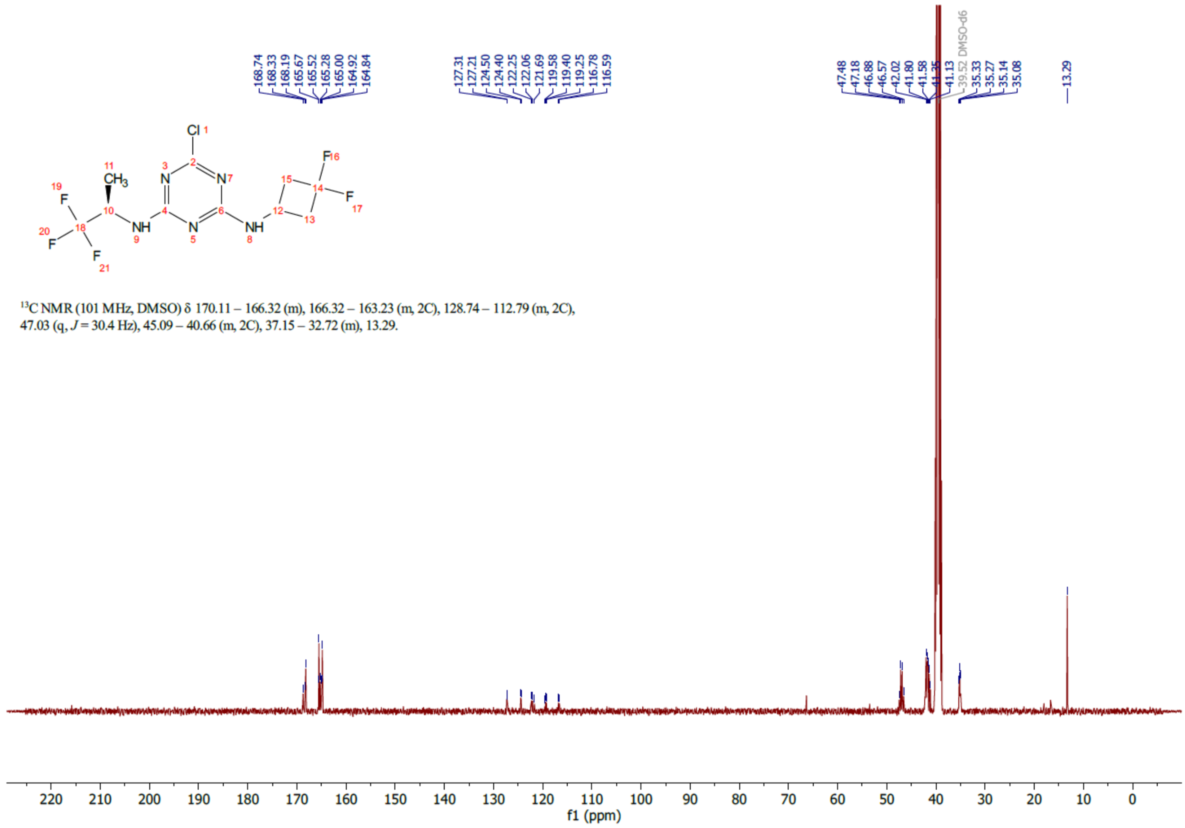

Figure S5. NMR spectra of **AG-881**.

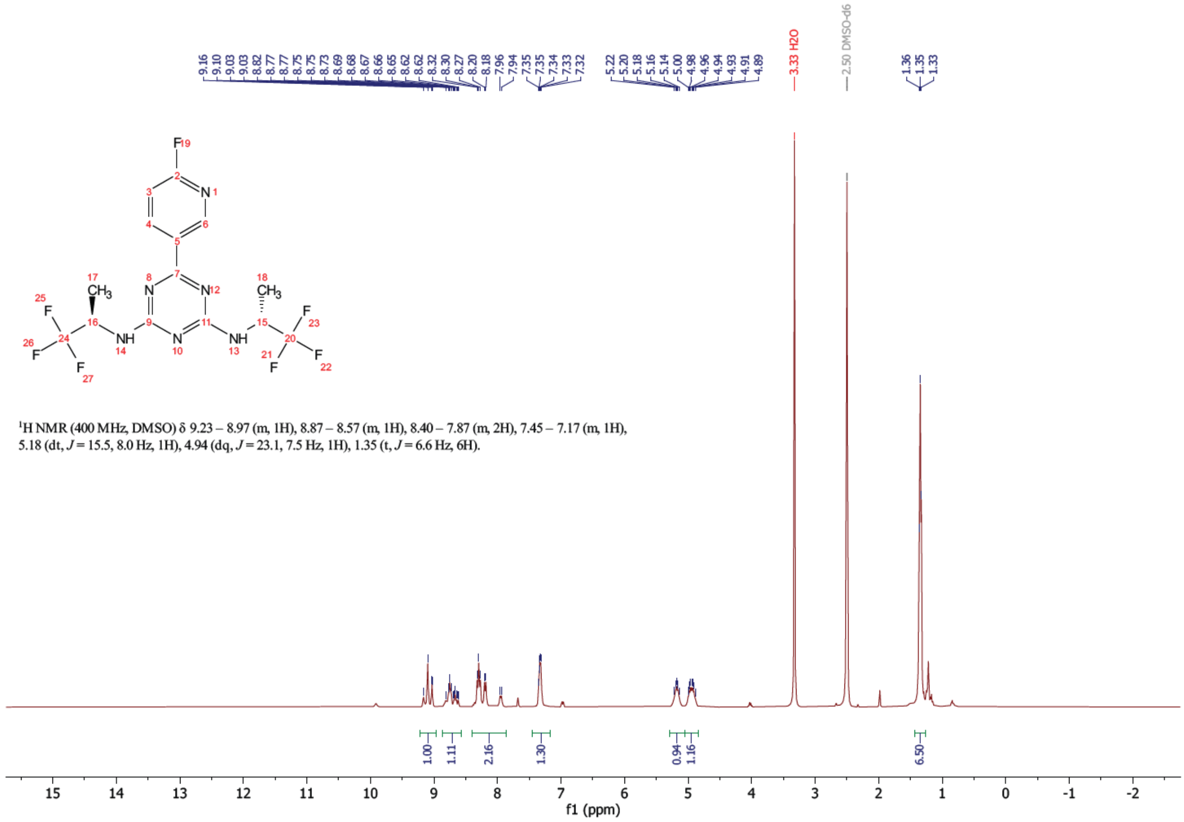

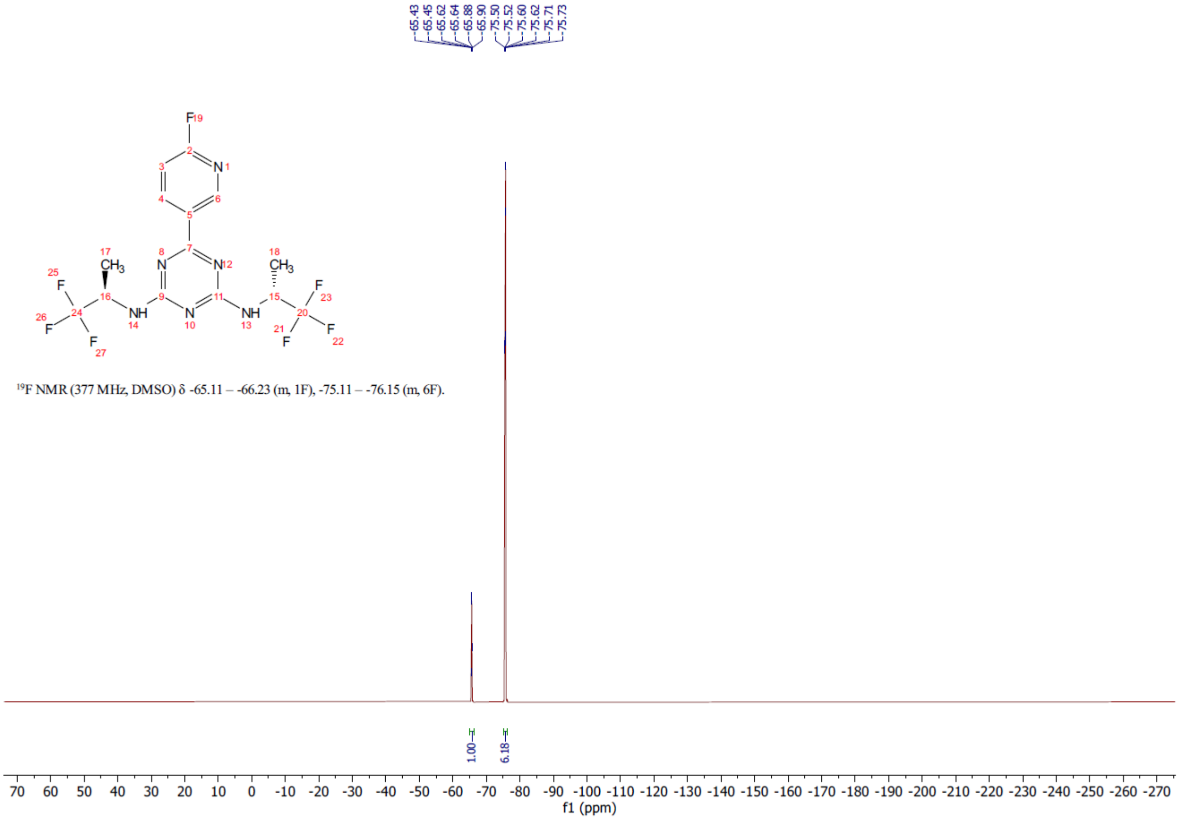

^{19}F NMR (377 MHz, DMSO) δ -65.11 – -66.23 (m, 1F), -75.11 – -76.15 (m, 6F).

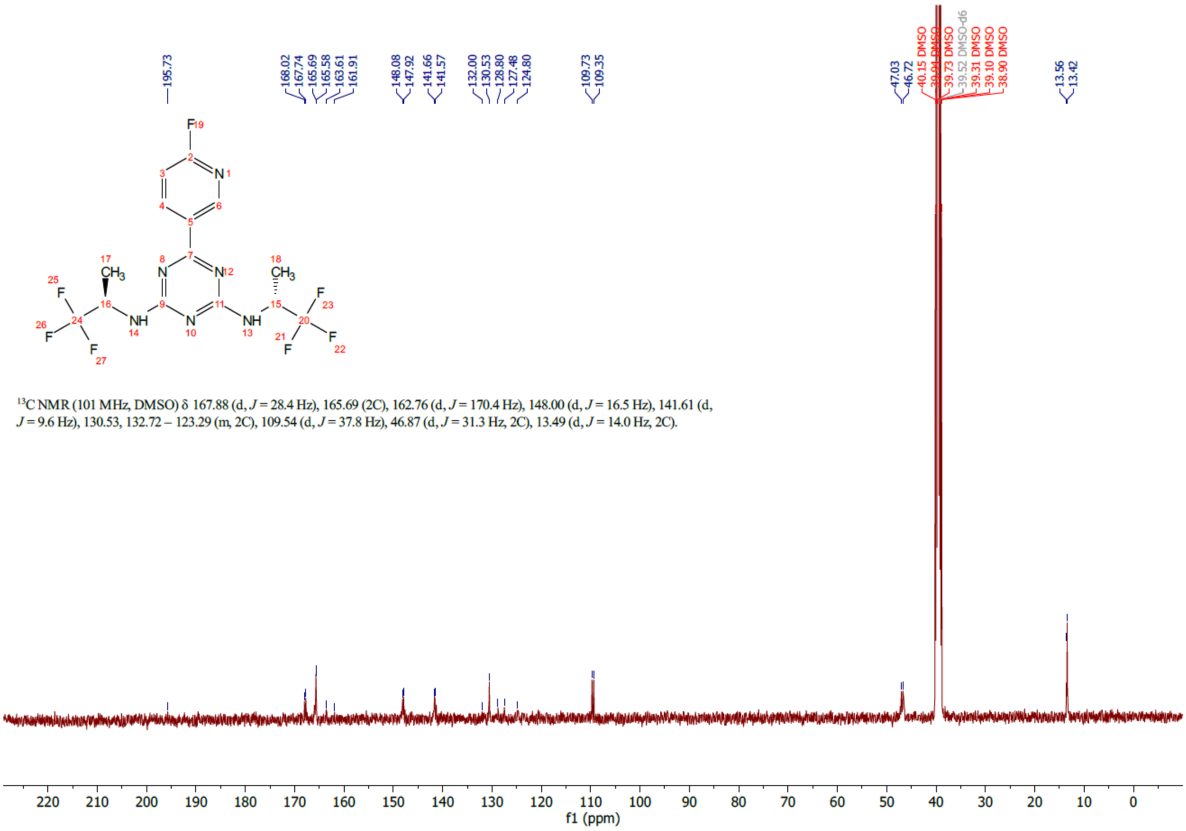

^{13}C NMR (101 MHz, DMSO) δ 167.88 (d, J = 28.4 Hz), 165.69 (2C), 162.76 (d, J = 170.4 Hz), 148.00 (d, J = 16.5 Hz), 141.61 (d, J = 9.6 Hz), 130.53, 132.72 – 123.29 (m, 2C), 109.54 (d, J = 37.8 Hz), 46.87 (d, J = 31.3 Hz, 2C), 13.49 (d, J = 14.0 Hz, 2C).

Figure S6. NMR spectra of 6-(5-chloro-6-fluoropyridin-3-yl)-*N*2,*N*4-bis((*R*)-1,1,1-trifluoropropan-2-yl)-1,3,5-triazine-2,4-diamine (**6**).

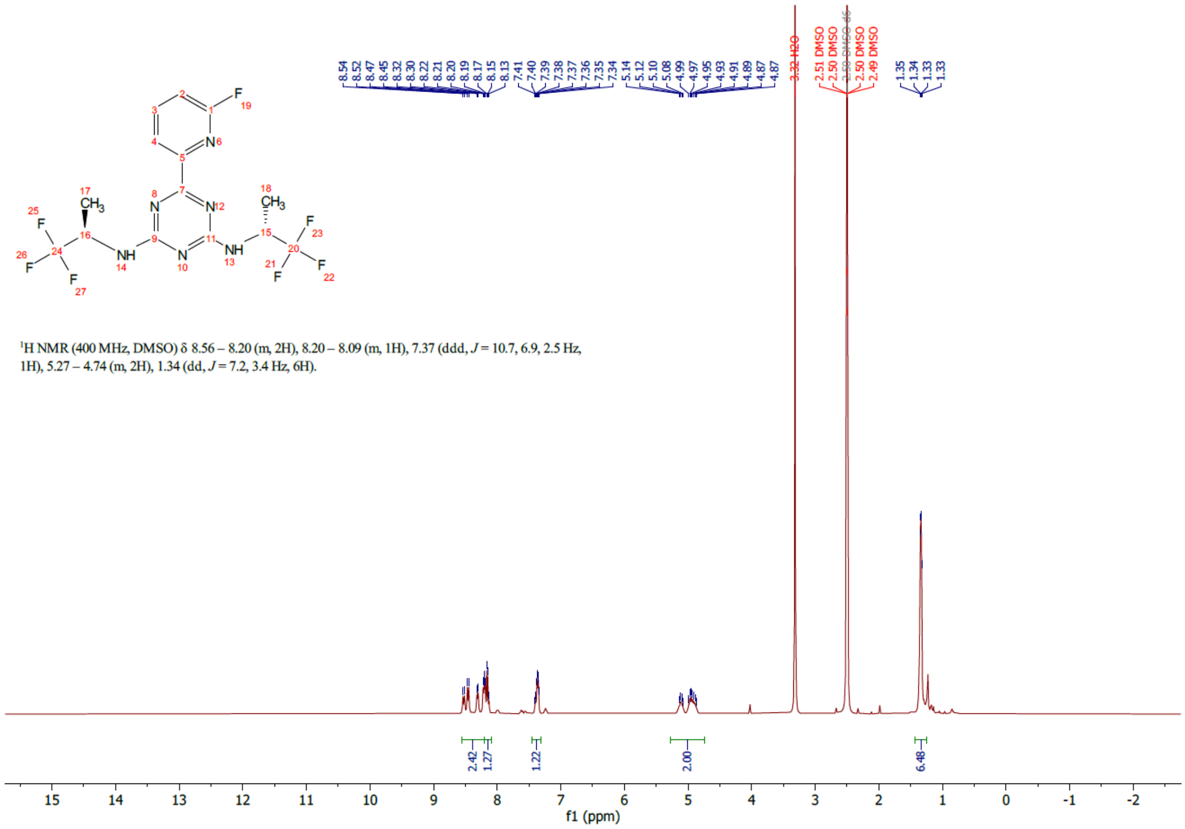

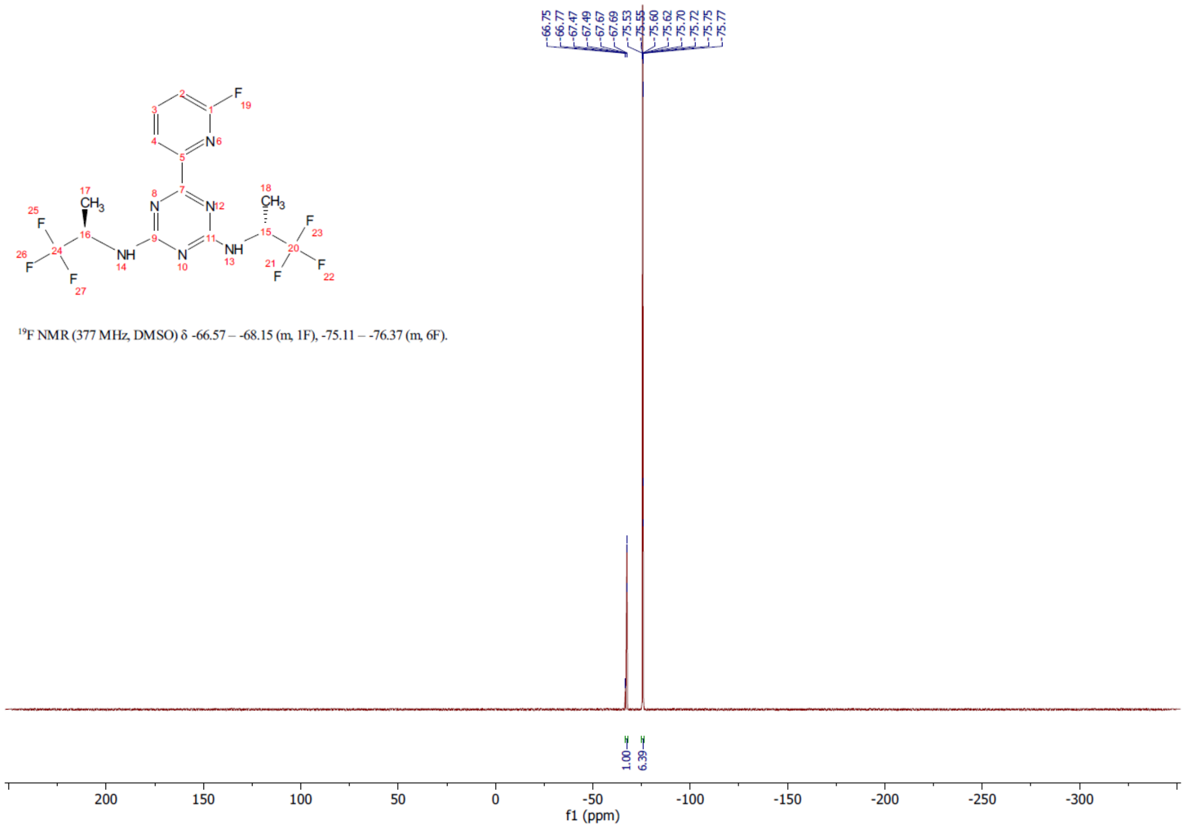

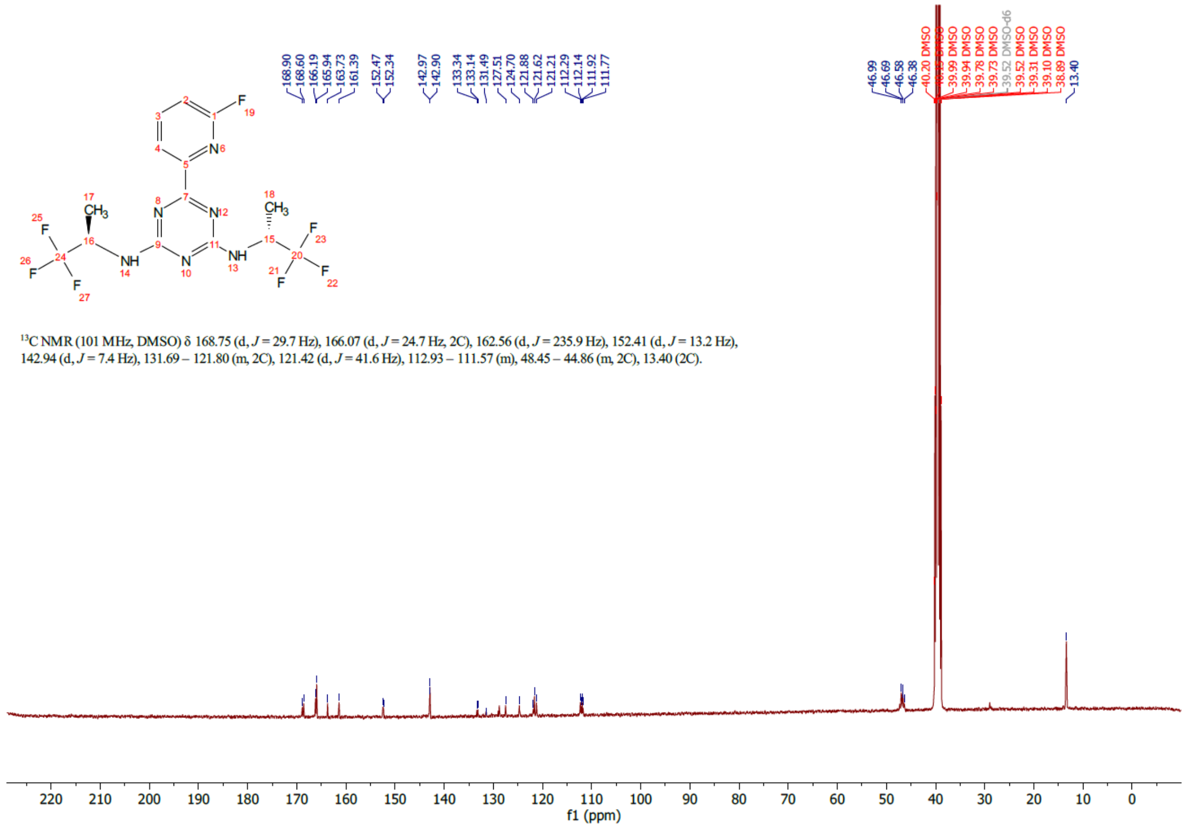

^{13}C NMR (101 MHz, DMSO) δ 168.75 (d, J = 29.7 Hz), 166.07 (d, J = 24.7 Hz, 2C), 162.56 (d, J = 235.9 Hz), 152.41 (d, J = 13.2 Hz), 142.94 (d, J = 7.4 Hz), 131.69 – 121.80 (m, 2C), 121.42 (d, J = 41.6 Hz), 112.93 – 111.57 (m), 48.45 – 44.86 (m, 2C), 13.40 (2C).

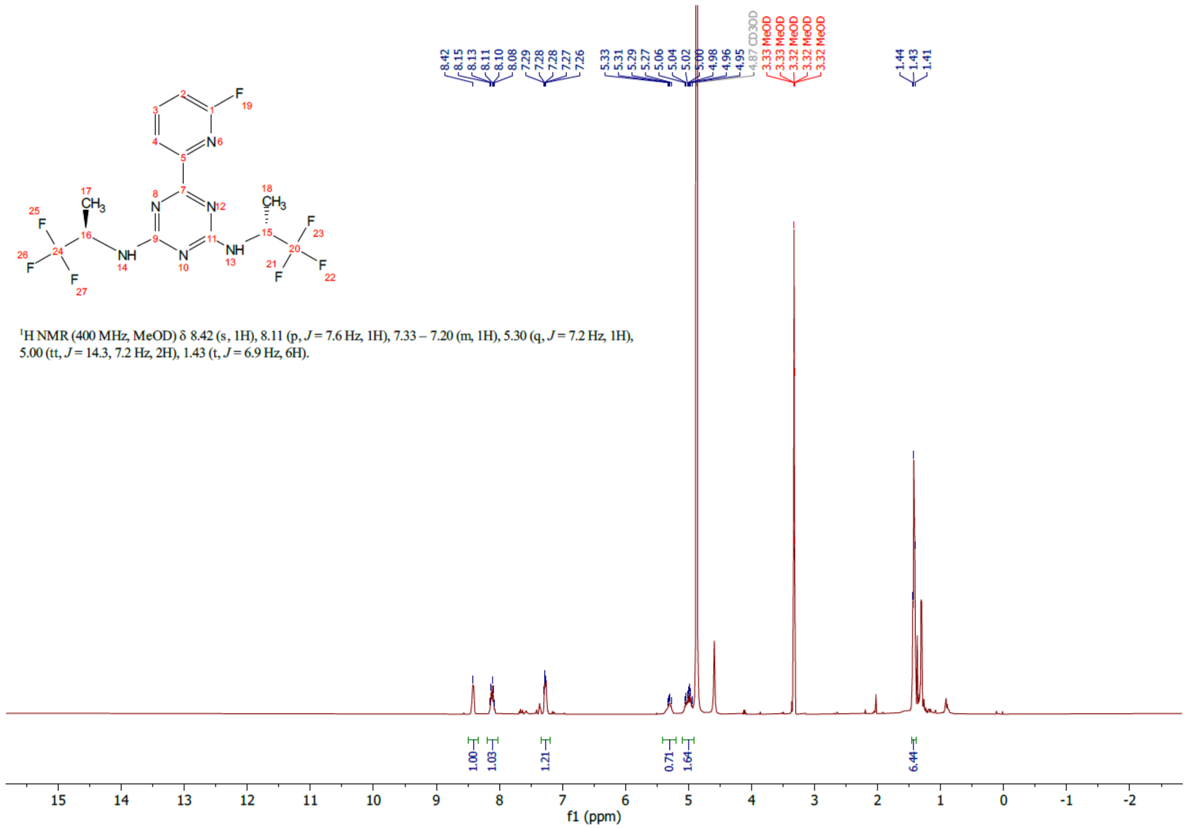

^{1}H NMR (400 MHz, MeOD) δ 8.42 (s, 1H), 8.11 (p, J = 7.6 Hz, 1H), 7.33 – 7.20 (m, 1H), 5.30 (q, J = 7.2 Hz, 1H), 5.00 (tt, J = 14.3, 7.2 Hz, 2H), 1.43 (t, J = 6.9 Hz, 6H).

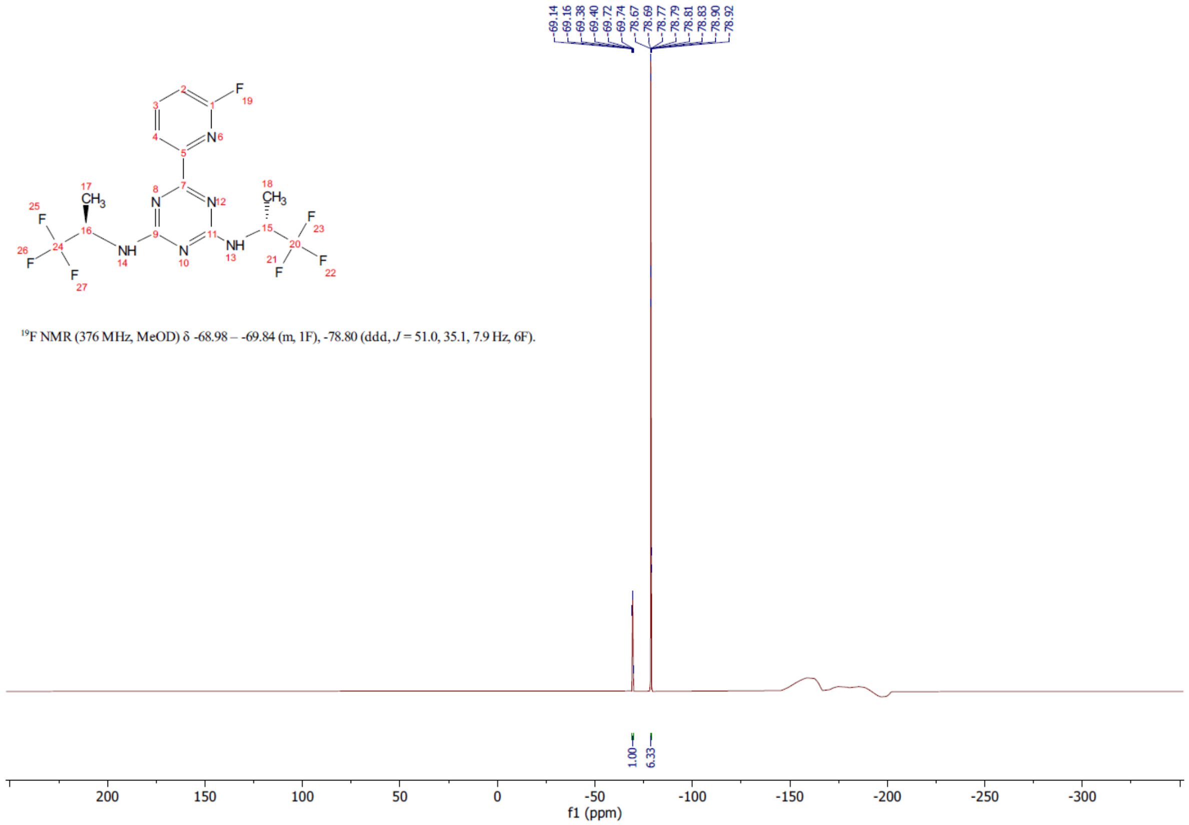

^{19}F NMR (376 MHz, MeOD) δ -68.98 – -69.84 (m, 1F), -78.80 (ddd, *J* = 51.0, 35.1, 7.9 Hz, 6F).

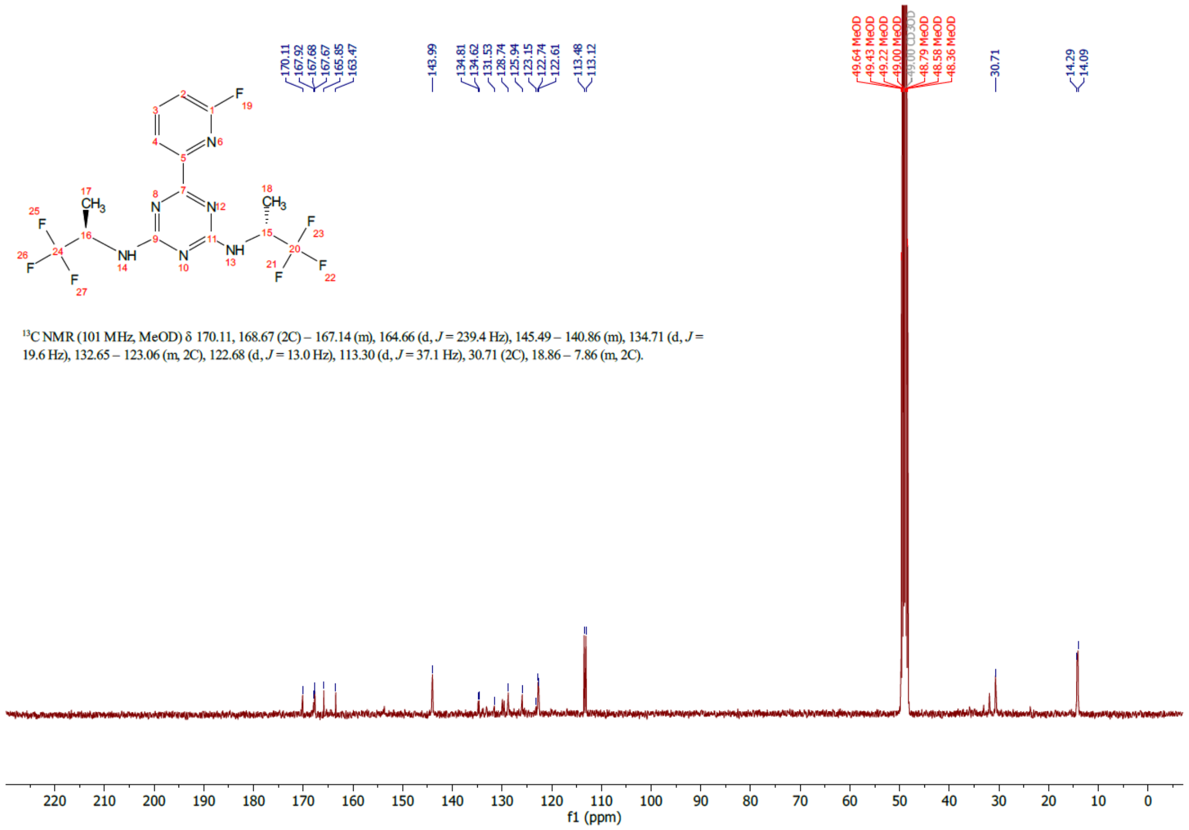

^{13}C NMR (101 MHz, MeOD) δ 170.11, 168.67 (2C) – 167.14 (m), 164.66 (d, *J* = 239.4 Hz), 145.49 – 140.86 (m), 134.71 (d, *J* = 19.6 Hz), 132.65 – 123.06 (m, 2C), 122.68 (d, *J* = 13.0 Hz), 113.30 (d, *J* = 37.1 Hz), 30.71 (2C), 18.86 – 7.86 (m, 2C).

Figure S7. NMR spectra of 6-(6-fluoropyridin-2-yl)-*N*[2],*N*[4]-bis((*R*)-1,1,1-trifluoropropan-2-yl)-1,3,5-triazine-2,4-diamine (**7**).

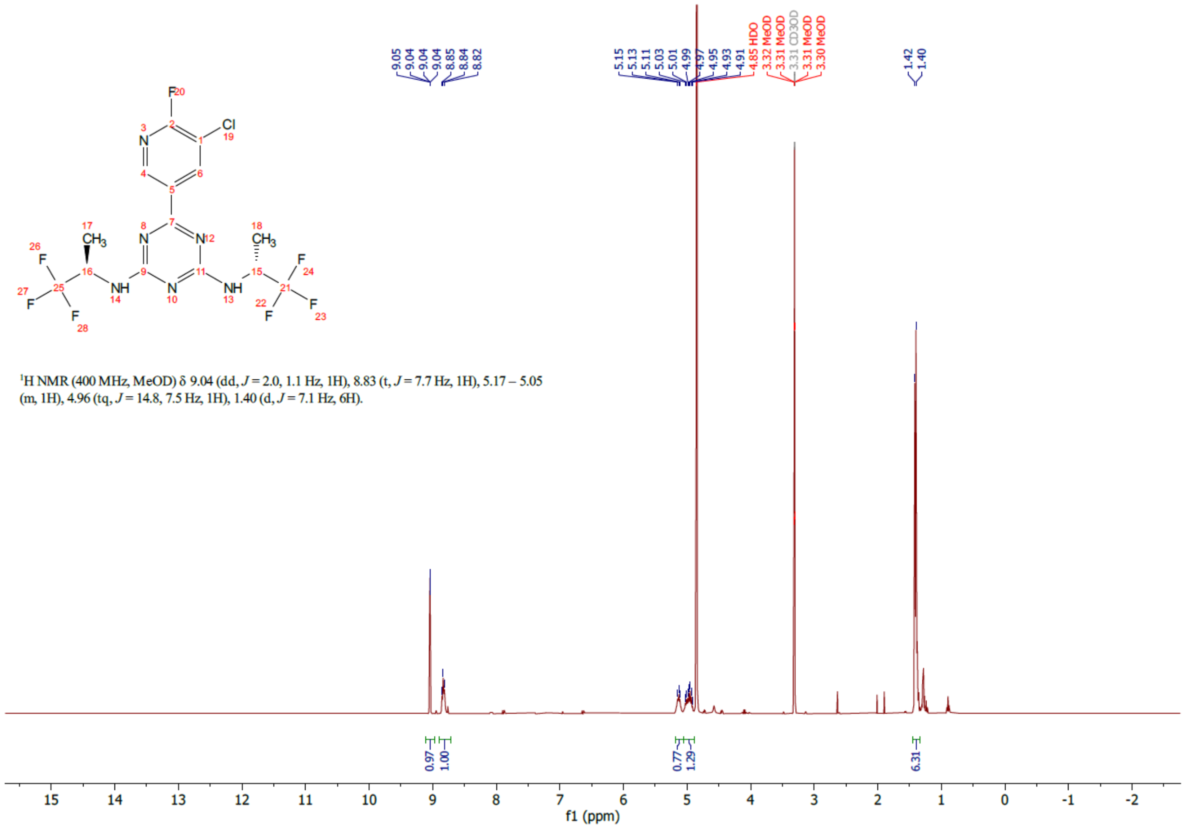

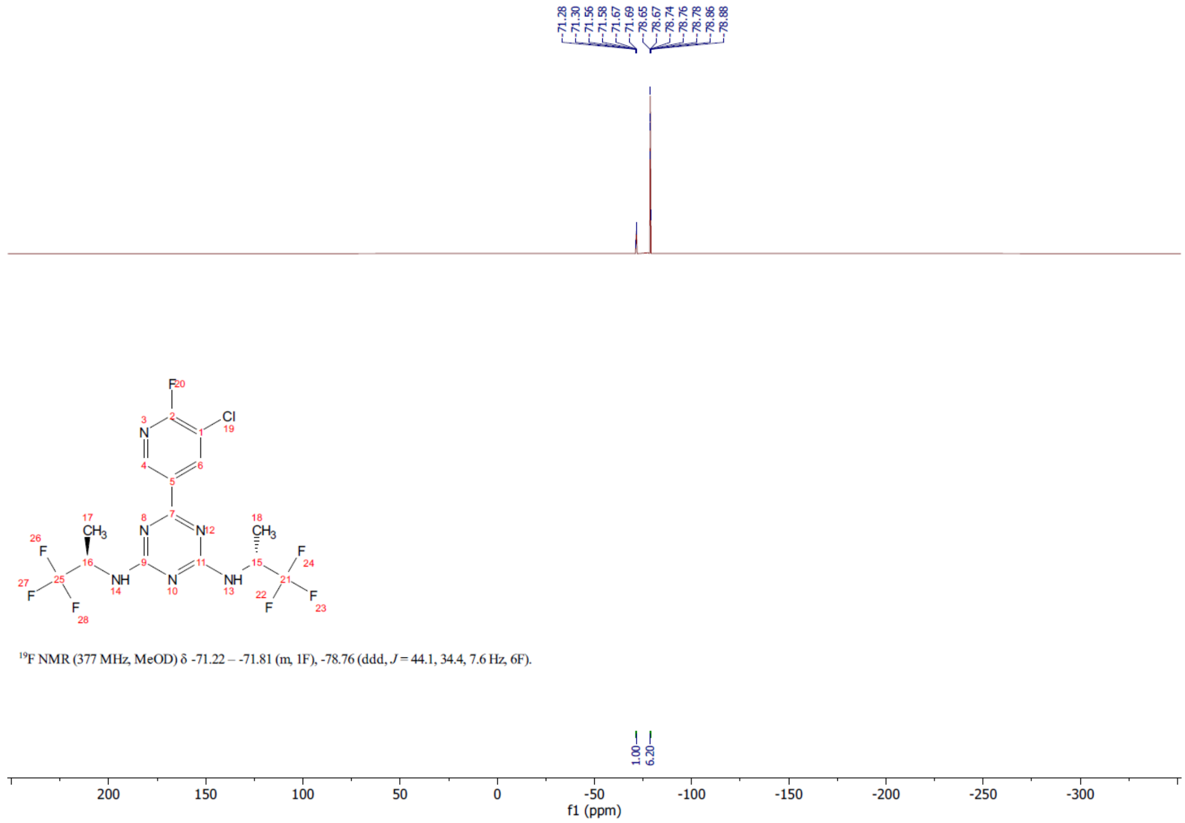

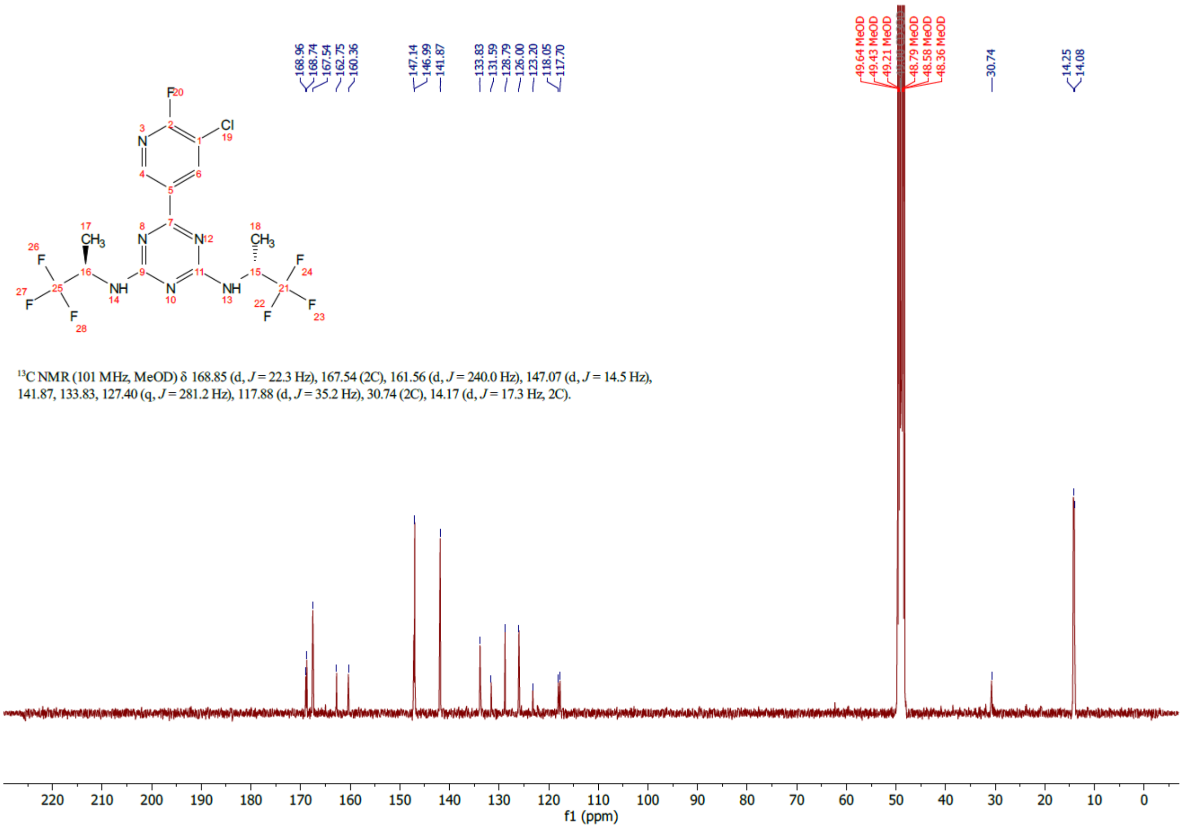

Figure S8. NMR spectra of 6-(6-fluoropyridin-3-yl)-N^2,N^4-bis((*R*)-1,1,1-trifluoropropan-2-yl)-1,3,5-triazine-2,4-diamine (**8**).

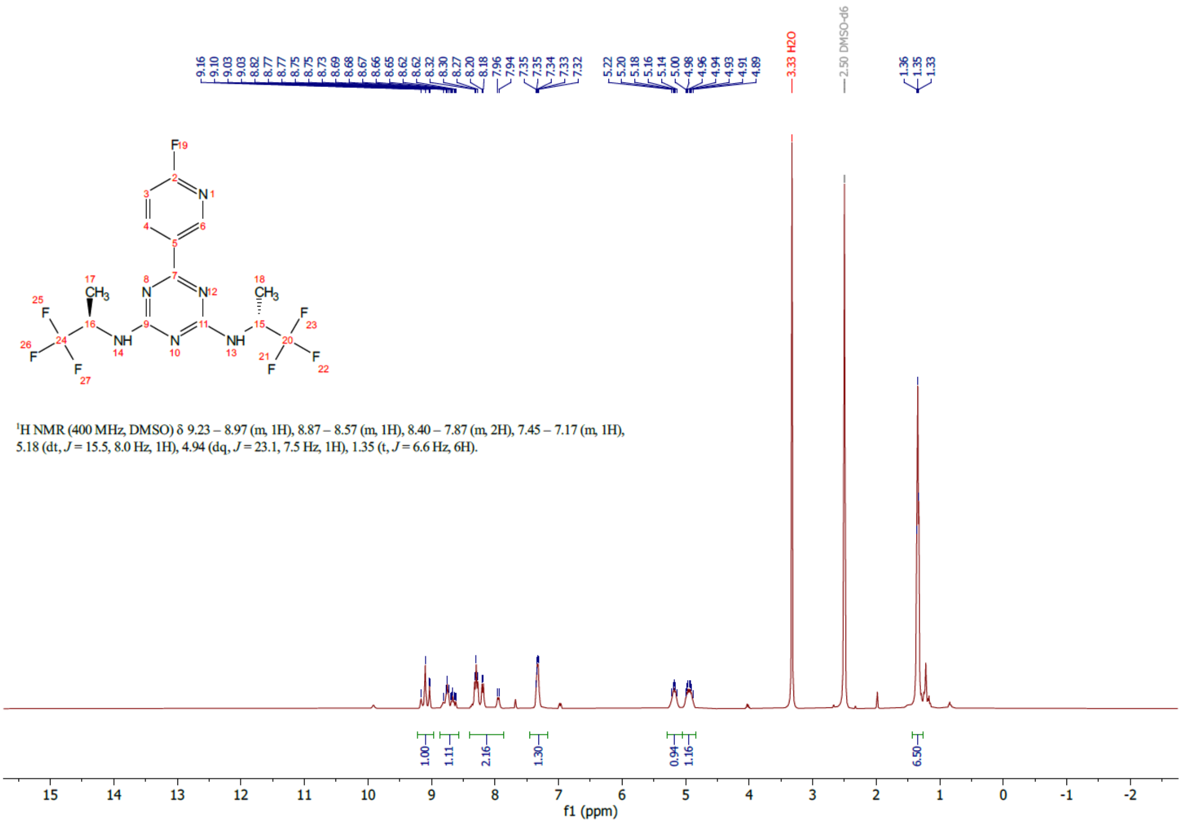

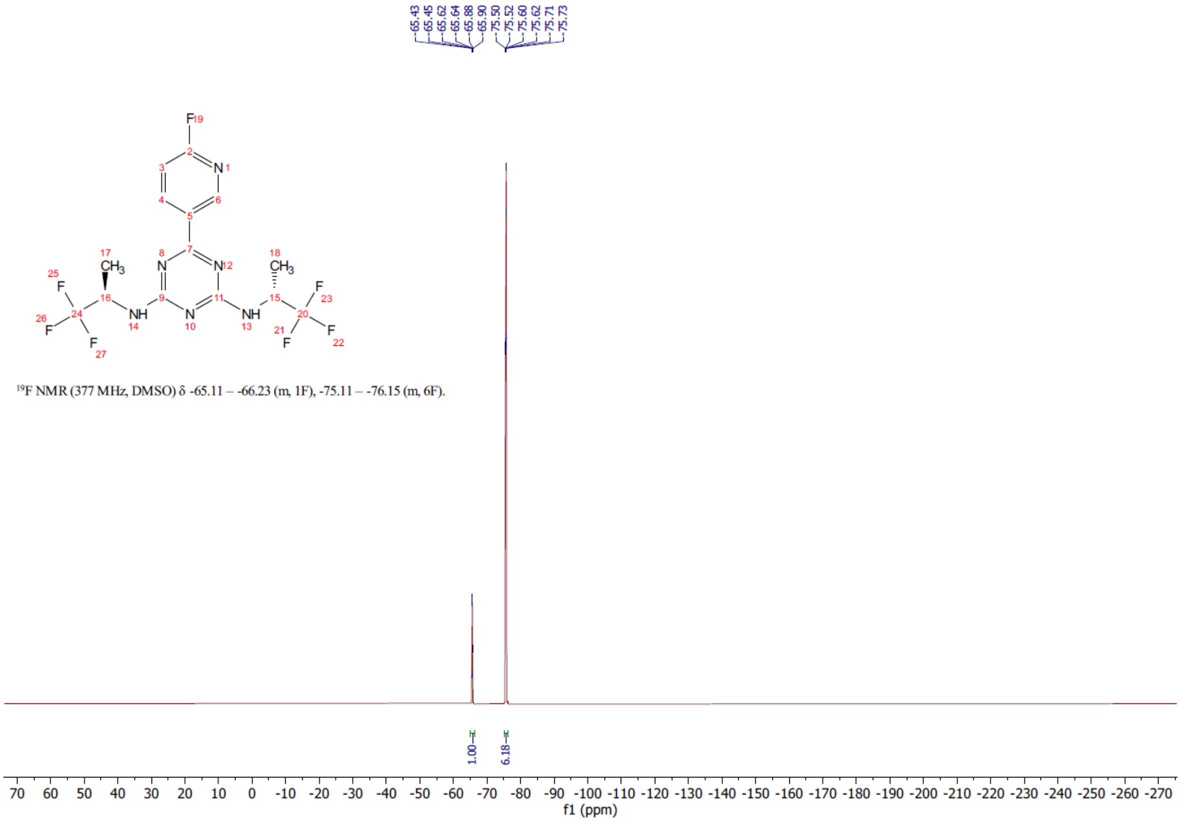

^{19}F NMR (377 MHz, DMSO) δ -65.11 – -66.23 (m, 1F), -75.11 – -76.15 (m, 6F).

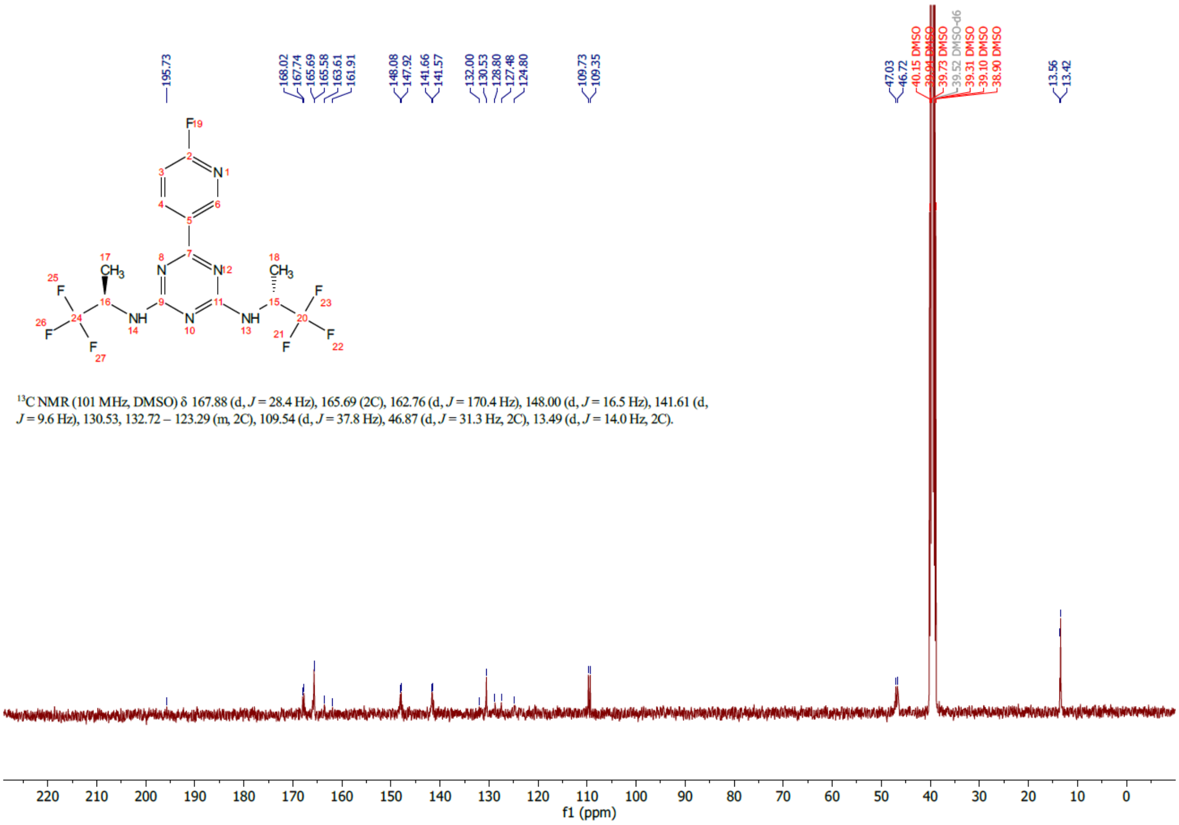

^{13}C NMR (101 MHz, DMSO) δ 167.88 (d, J = 28.4 Hz), 165.69 (2C), 162.76 (d, J = 170.4 Hz), 148.00 (d, J = 16.5 Hz), 141.61 (d, J = 9.6 Hz), 130.53, 132.72 – 123.29 (m, 2C), 109.54 (d, J = 37.8 Hz), 46.87 (d, J = 31.3 Hz, 2C), 13.49 (d, J = 14.0 Hz, 2C).

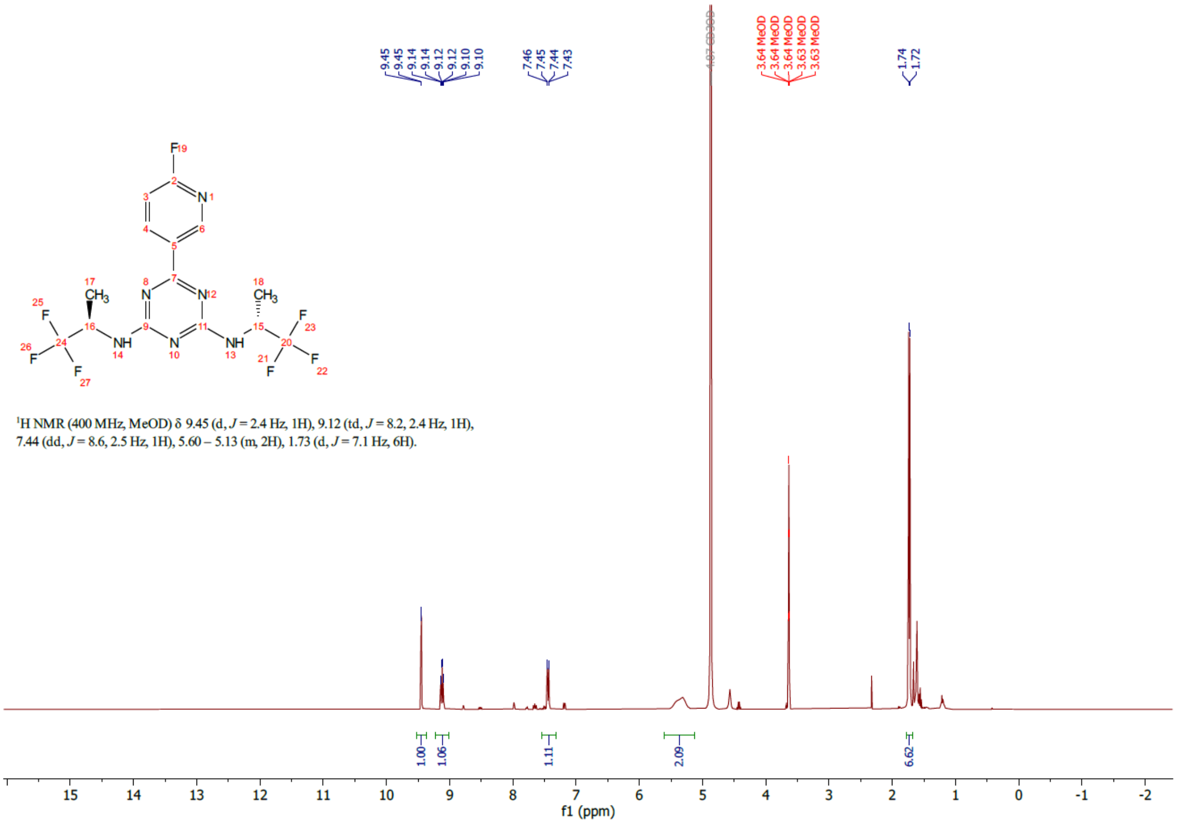

^{1}H NMR (400 MHz, MeOD) δ 9.45 (d, *J* = 2.4 Hz, 1H), 9.12 (td, *J* = 8.2, 2.4 Hz, 1H), 7.44 (dd, *J* = 8.6, 2.5 Hz, 1H), 5.60 – 5.13 (m, 2H), 1.73 (d, *J* = 7.1 Hz, 6H).

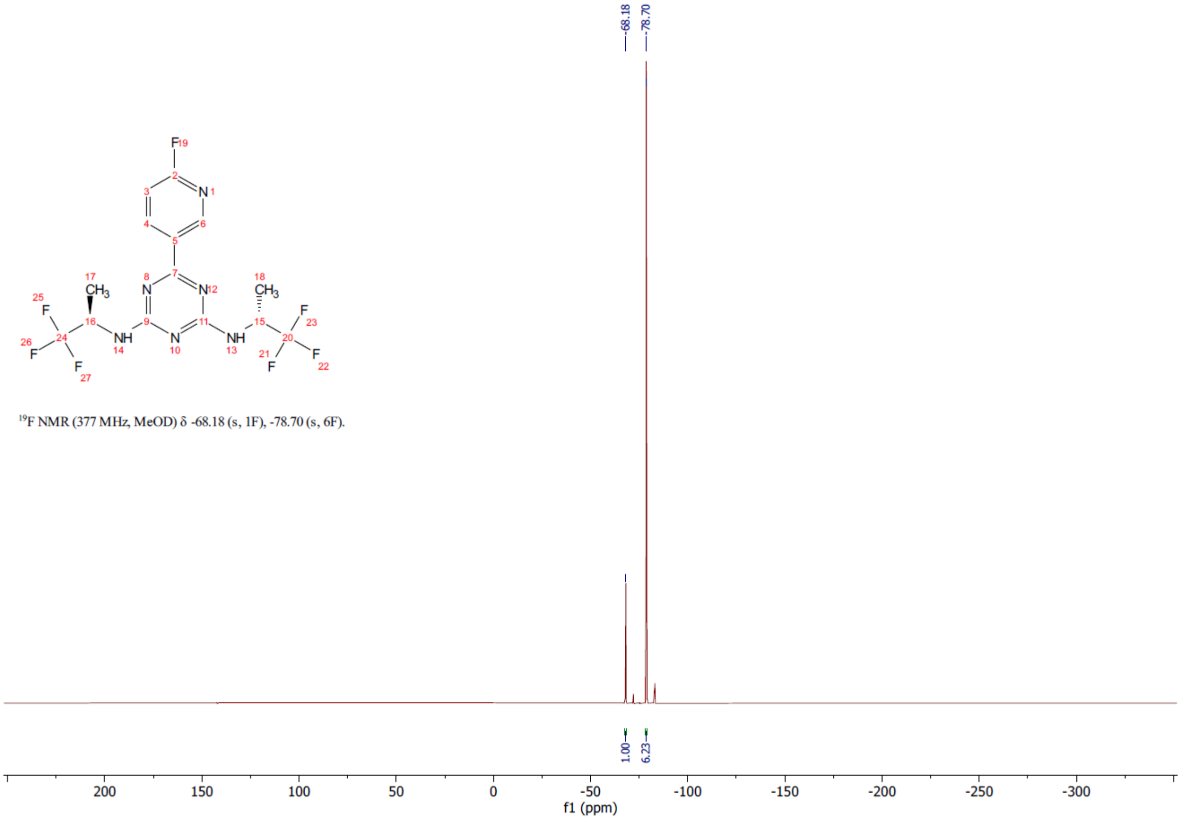

^{19}F NMR (377 MHz, MeOD) δ -68.18 (s, 1F), -78.70 (s, 6F).

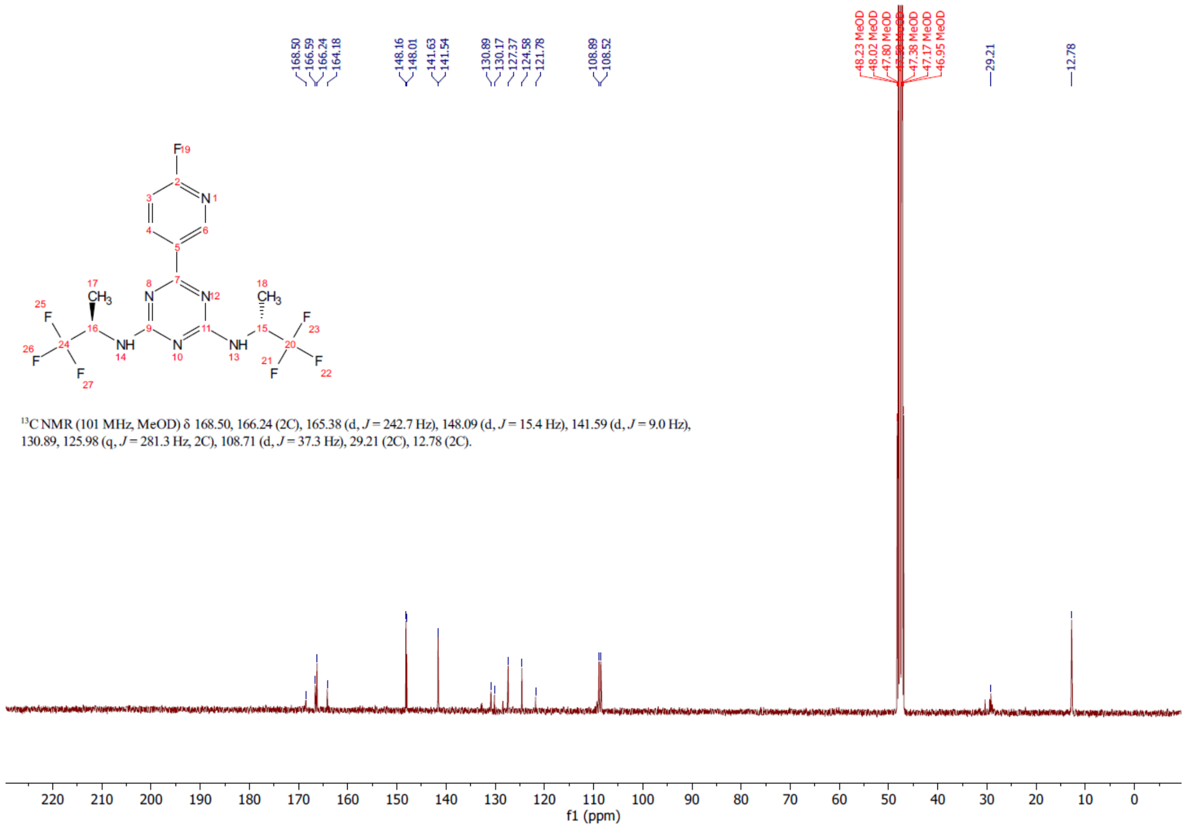

Figure S9. NMR spectra of 6-(5-chloro-6-fluoropyridin-3-yl)-N^2,N^4-bis((*R*)-1,1,1-trifluorobutan-2-yl)-1,3,5-triazine-2,4-diamine (**9**).

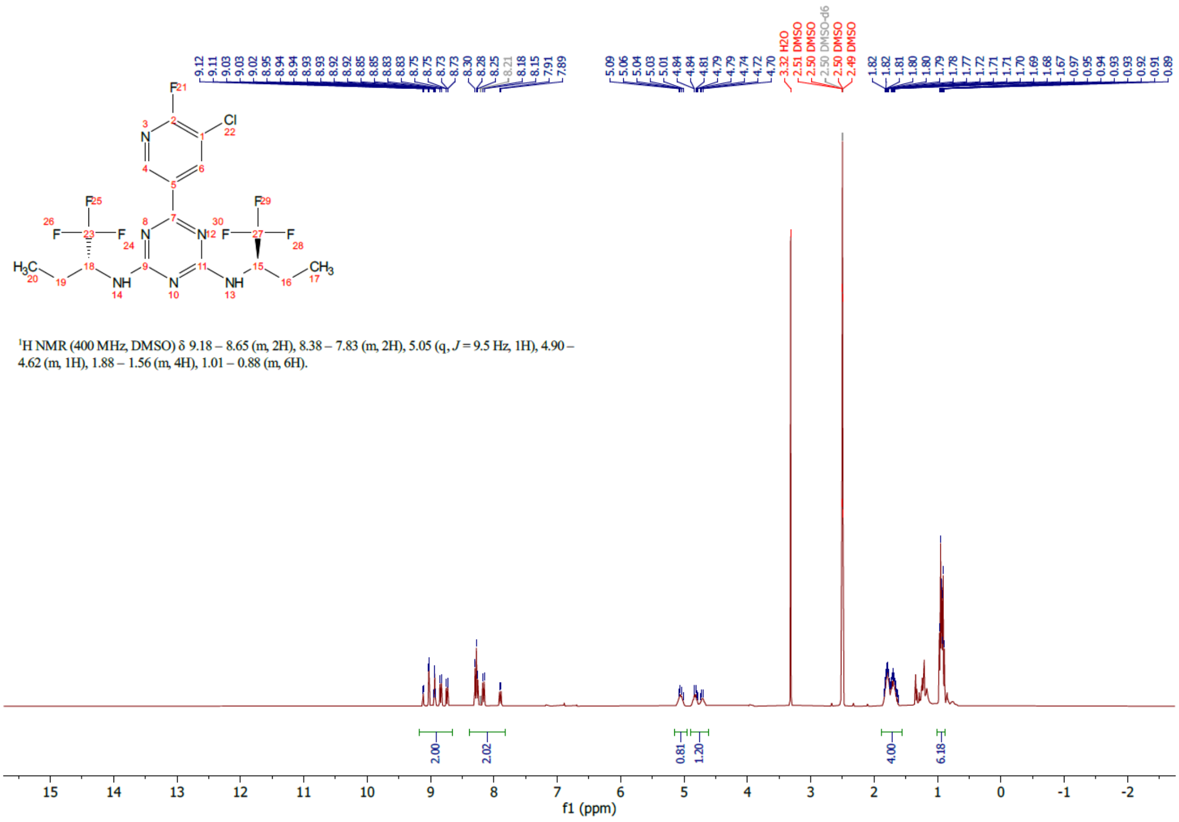

^{19}F NMR (377 MHz, DMSO) δ -69.32 – -70.70 (m, 1F), -73.93 – -74.67 (m, 6F).

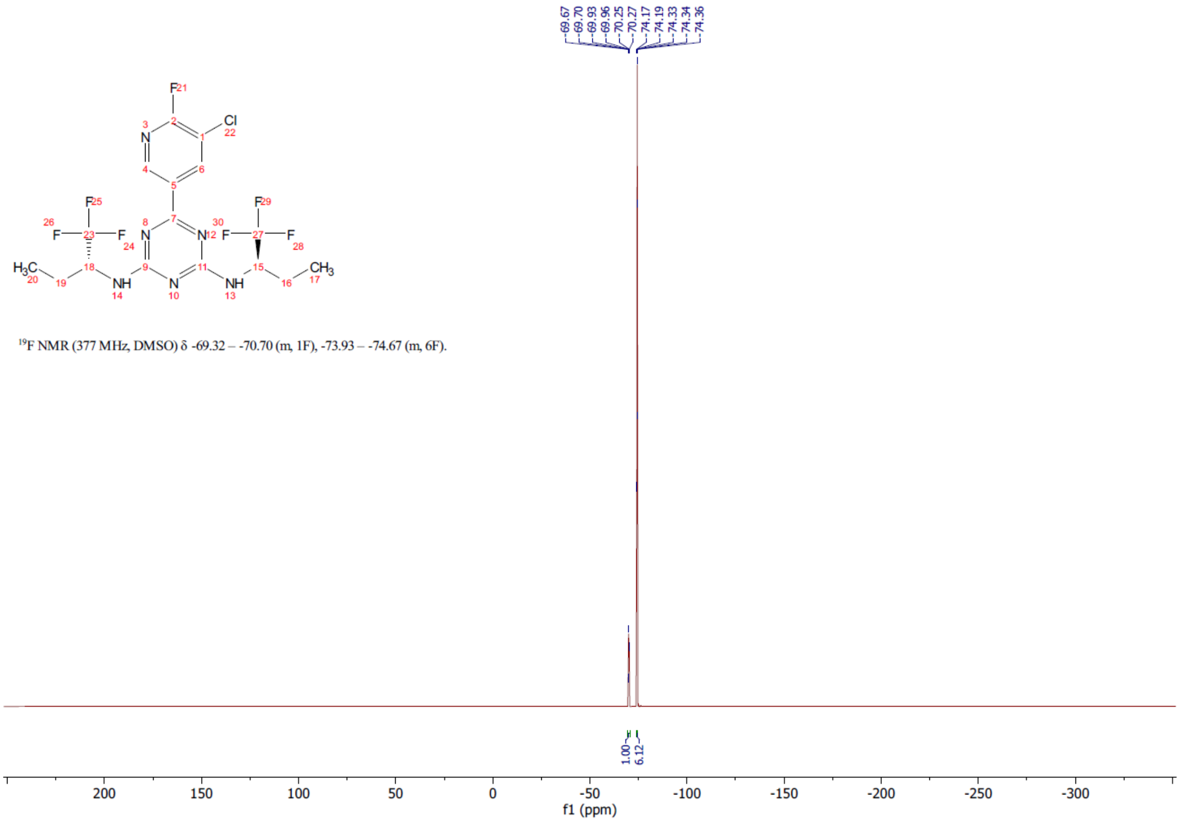

^{13}C NMR (101 MHz, DMSO) δ 166.86 (d, J = 26.6 Hz), 166.38 (d, J = 17.2 Hz, 2C), 161.88 – 155.28 (m), 145.67 (td, J = 42.3, 14.9 Hz), 140.50 (d, J = 7.9 Hz), 132.25 (d, J = 5.2 Hz), 132.45 – 118.97 (m, 2C), 117.00 – 112.08 (m), 52.43 (q, J = 29.0 Hz, 2C), 20.36 (d, J = 7.7 Hz, 2C), 14.80 – 2.86 (m, 2C).

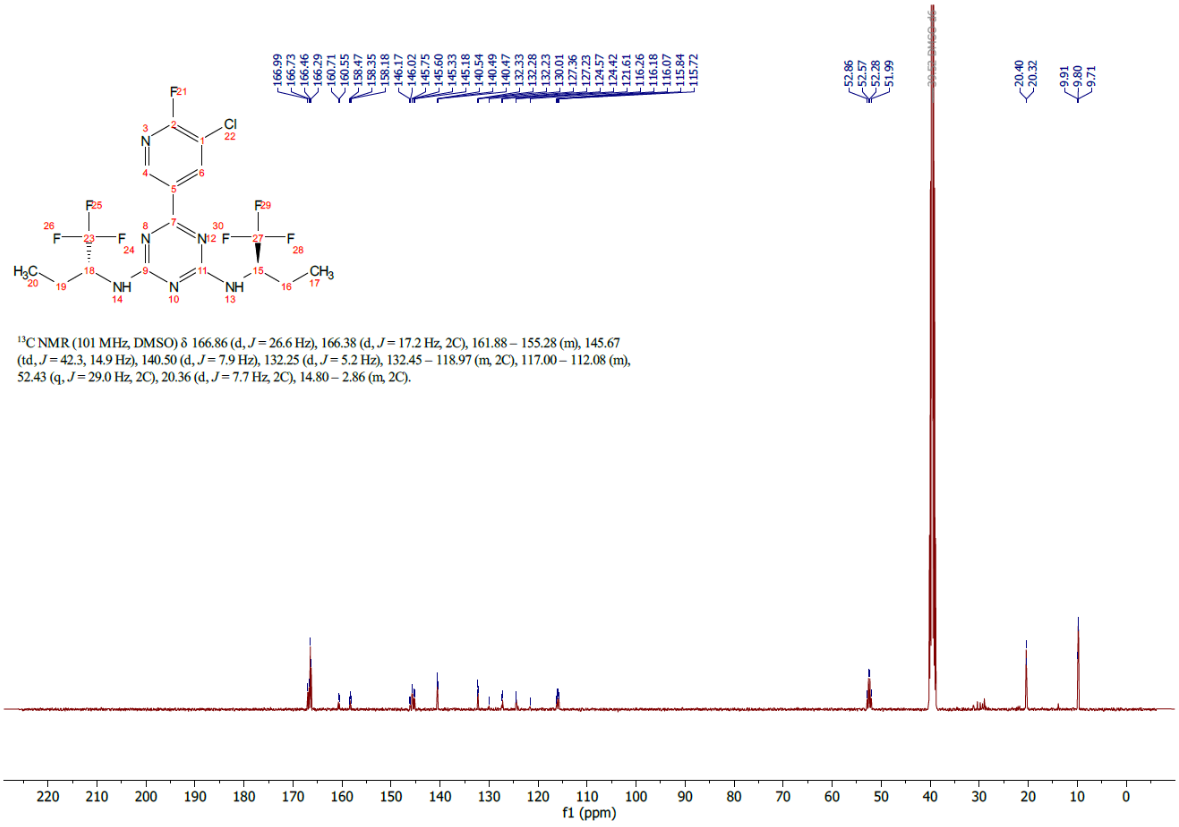

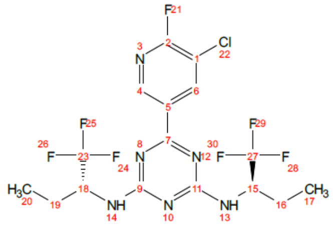

^{1}H NMR (400 MHz, MeOD) δ 9.07 (dd, J = 4.5, 2.4 Hz, 1H), 8.85 (tdd, J = 13.1, 8.9, 2.1 Hz, 1H), 4.96 (ddt, J = 14.7, 7.4, 3.5 Hz, 1H), 4.79 (ddp, J = 11.2, 7.7, 4.0 Hz, 1H), 1.93 (tqd, J = 11.7, 8.0, 3.8 Hz, 2H), 1.71 (ddq, J = 14.5, 11.0, 7.4 Hz, 2H), 1.04 (q, J = 7.3 Hz, 6H).

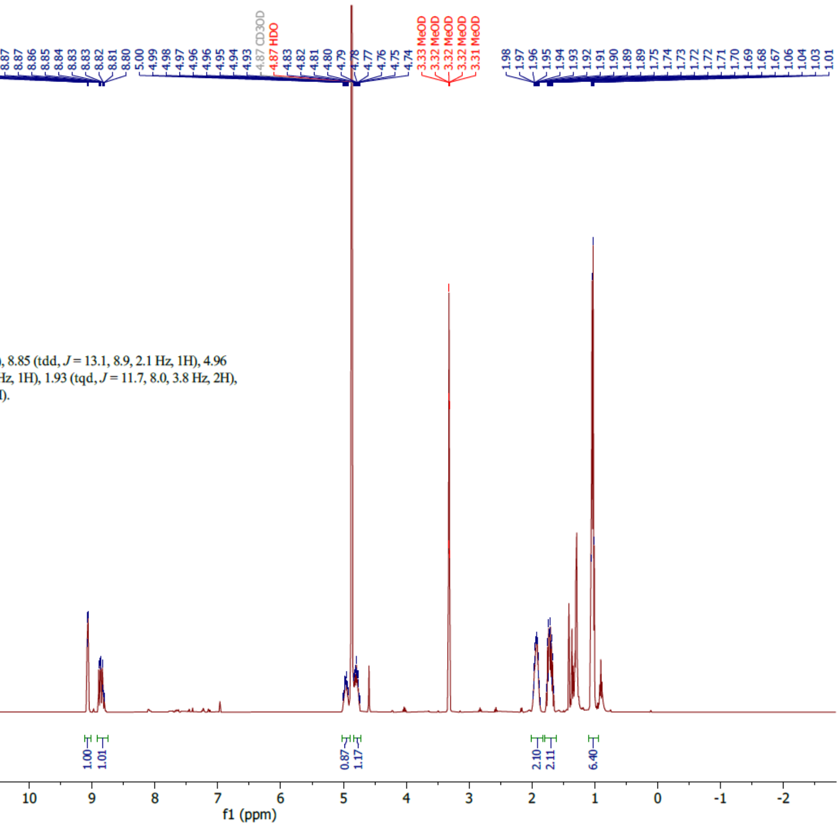

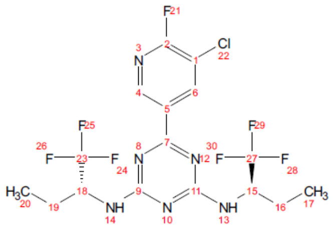

^{19}F NMR (376 MHz, MeOD) δ -71.18 – -71.80 (m, 1F), -77.21 – -77.59 (m, 6F).

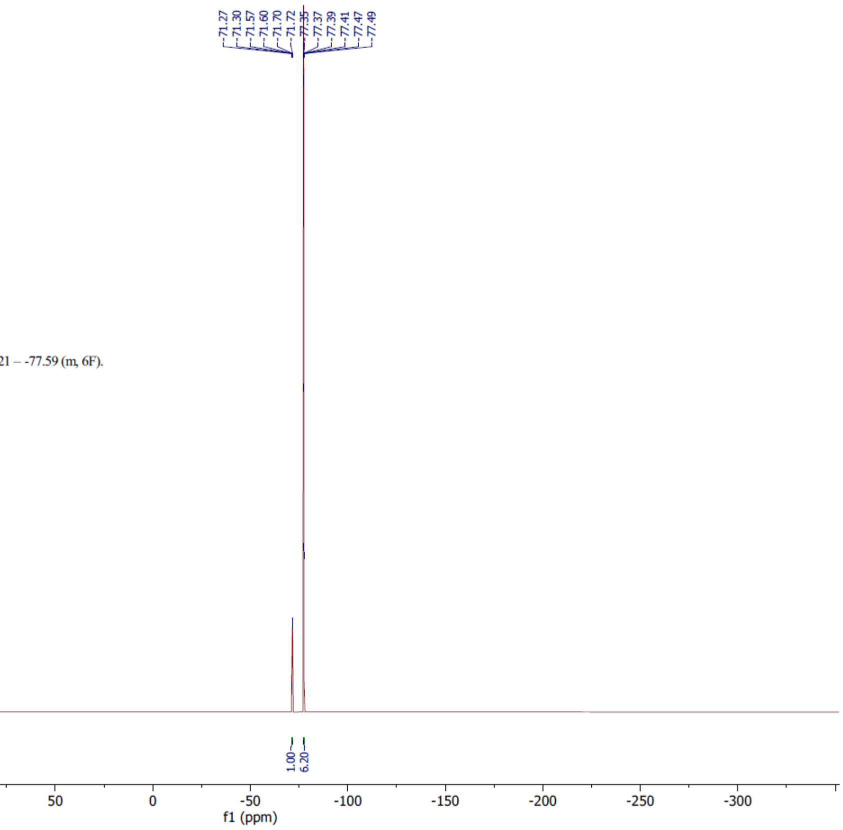

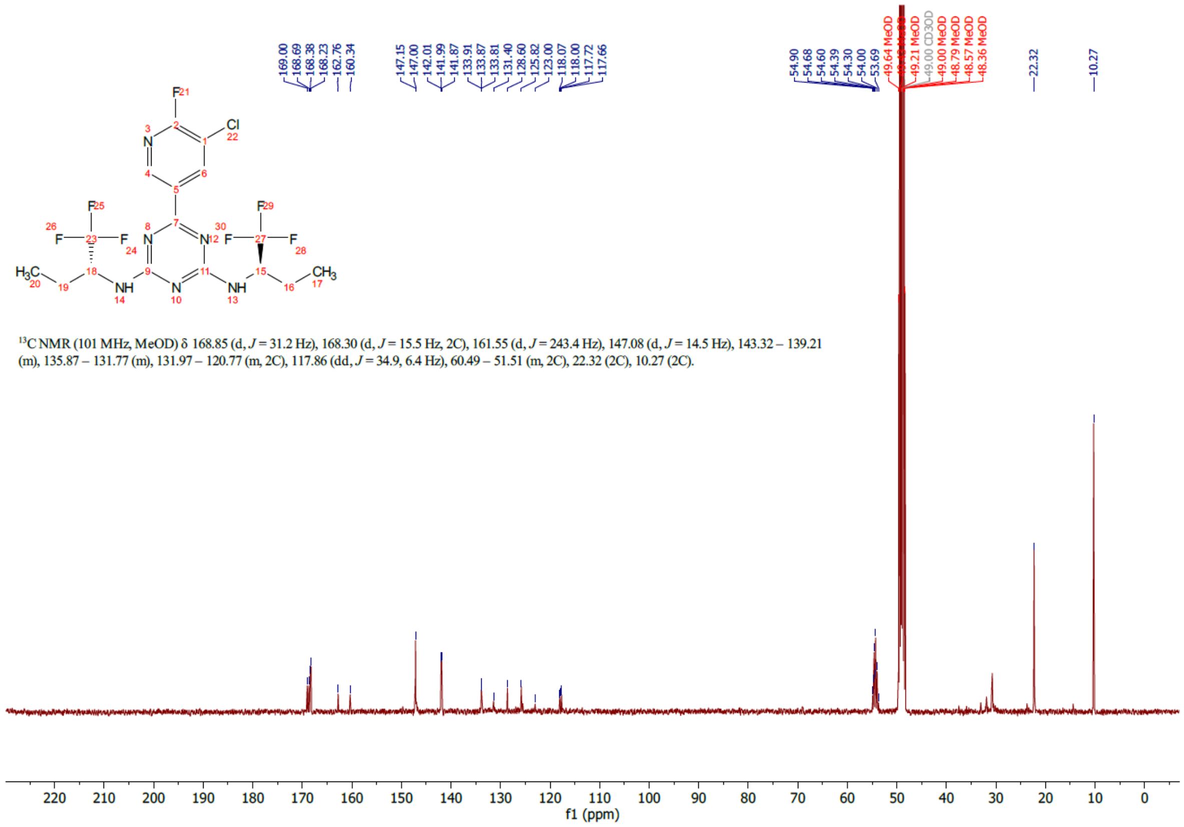

Figure S10. NMR spectra of 6-(6-fluoropyridin-3-yl)-N^2,N^4-bis((*R*)-1,1,1-trifluorobutan-2-yl)-1,3,5-triazine-2,4-diamine (**10**).

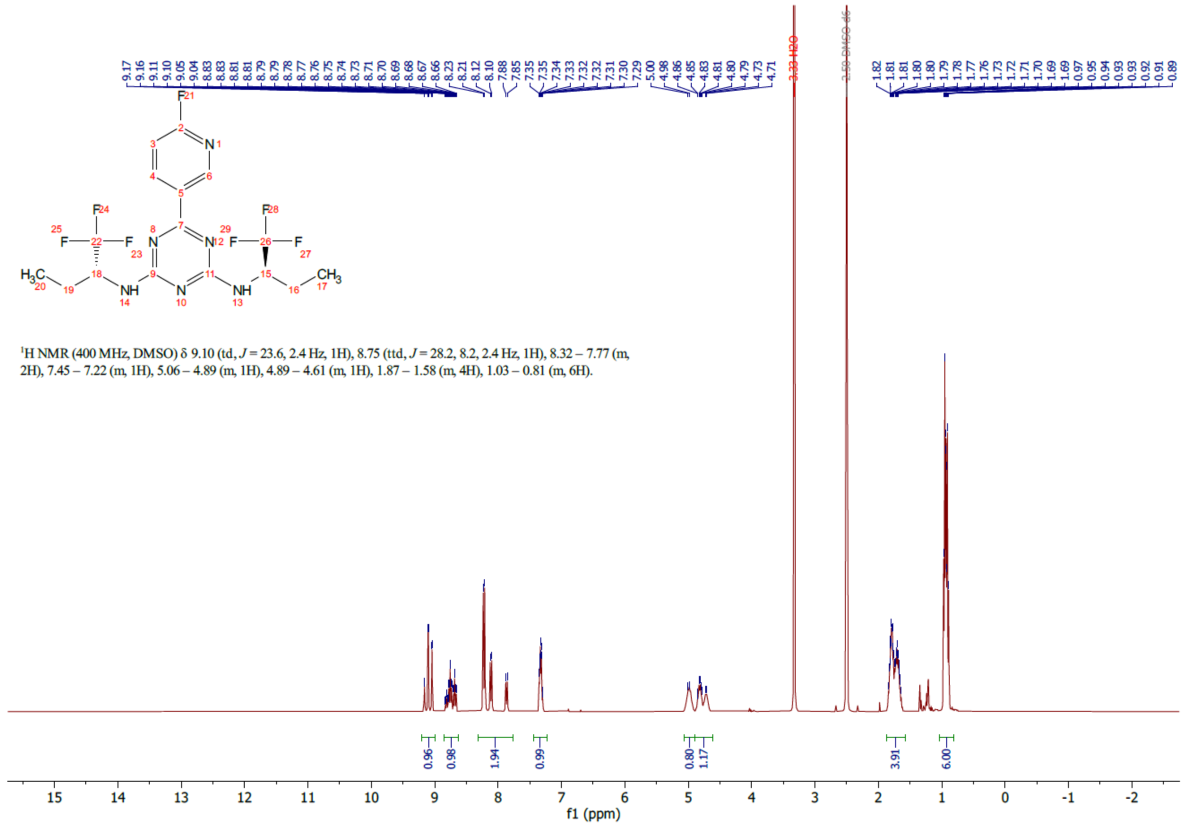

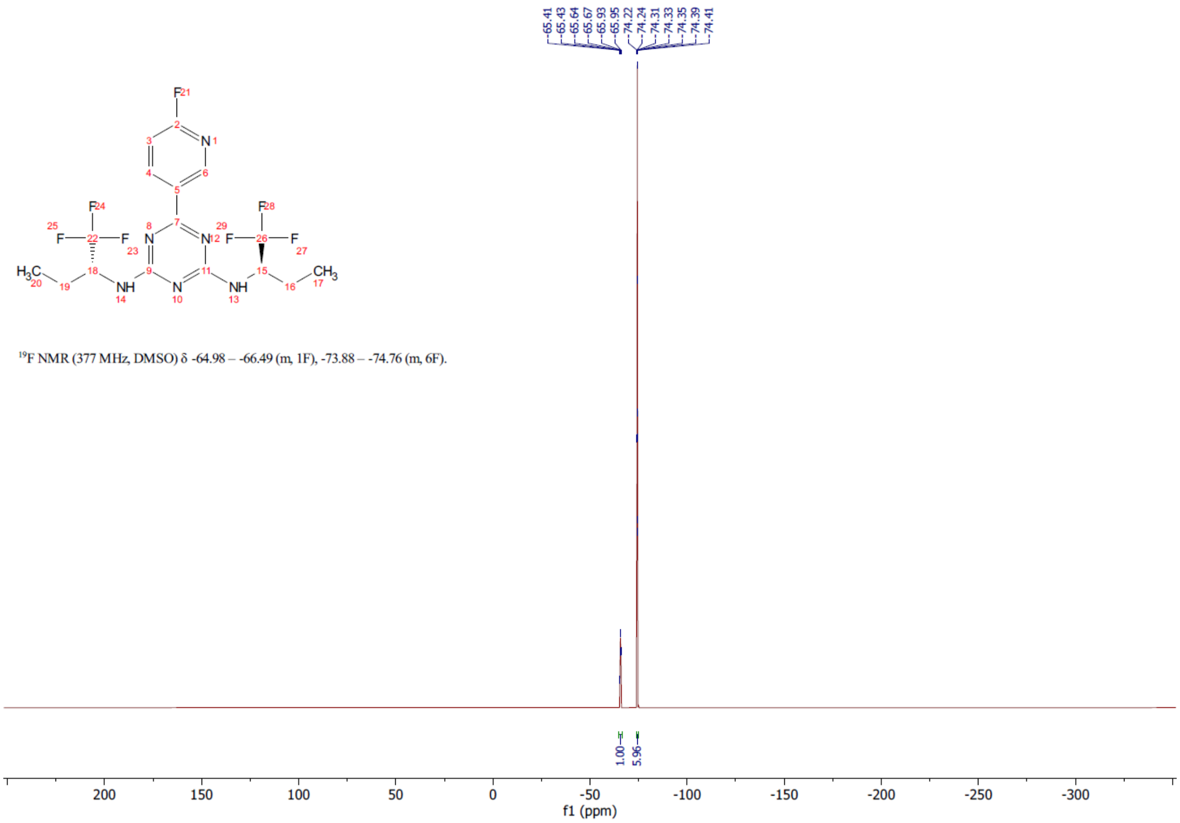

^{19}F NMR (377 MHz, DMSO) δ -64.98 – -66.49 (m, 1F), -73.88 – -74.76 (m, 6F).

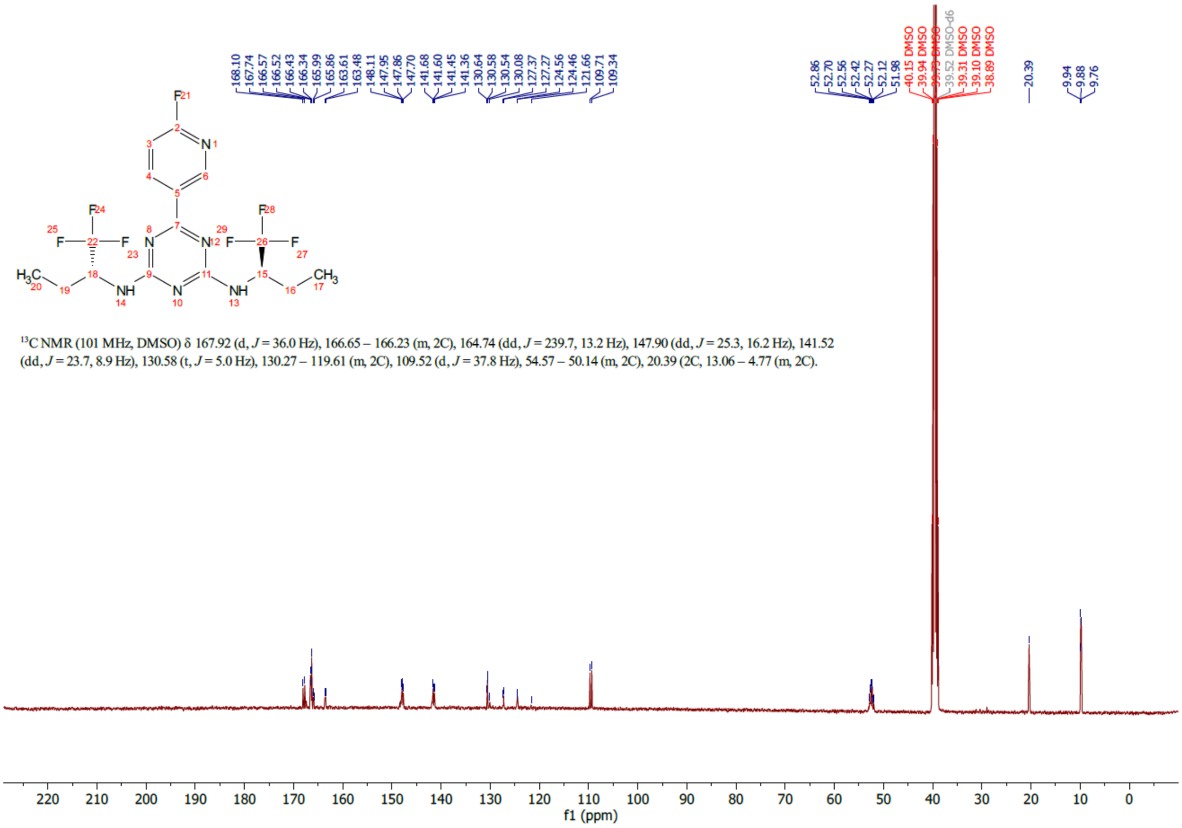

^{13}C NMR (101 MHz, DMSO) δ 167.92 (d, *J* = 36.0 Hz), 166.65 – 166.23 (m, 2C), 164.74 (dd, *J* = 239.7, 13.2 Hz), 147.90 (dd, *J* = 25.3, 16.2 Hz), 141.52 (dd, *J* = 23.7, 8.9 Hz), 130.58 (t, *J* = 5.0 Hz), 130.27 – 119.61 (m, 2C), 109.52 (d, *J* = 37.8 Hz), 54.57 – 50.14 (m, 2C), 20.39 (2C, 13.06 – 4.77 (m, 2C).

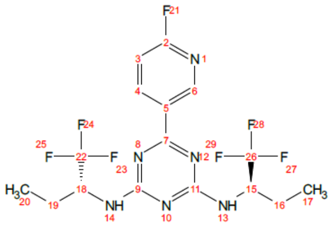

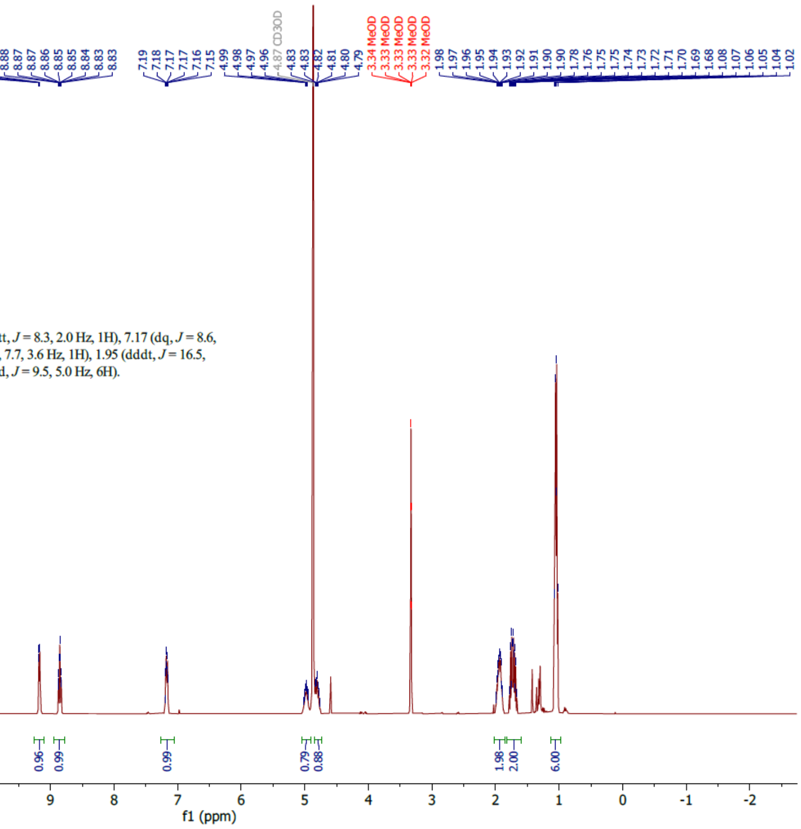

^{1}H NMR (400 MHz, MeOD) δ 9.18 (dd, *J* = 6.3, 2.4 Hz, 1H), 8.85 (tt, *J* = 8.3, 2.0 Hz, 1H), 7.17 (dq, *J* = 8.6, 3.5 Hz, 1H), 4.98 (dtd, *J* = 11.0, 7.2, 3.4 Hz, 1H), 4.80 (dtd, *J* = 11.2, 7.7, 3.6 Hz, 1H), 1.95 (dddt, *J* = 16.5, 12.6, 9.1, 3.9 Hz, 2H), 1.72 (ddq, *J* = 14.5, 11.0, 7.3 Hz, 2H), 1.04 (dd, *J* = 9.5, 5.0 Hz, 6H).

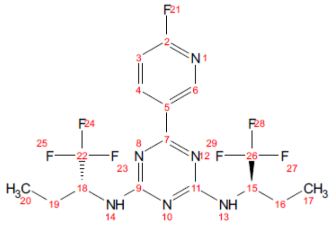

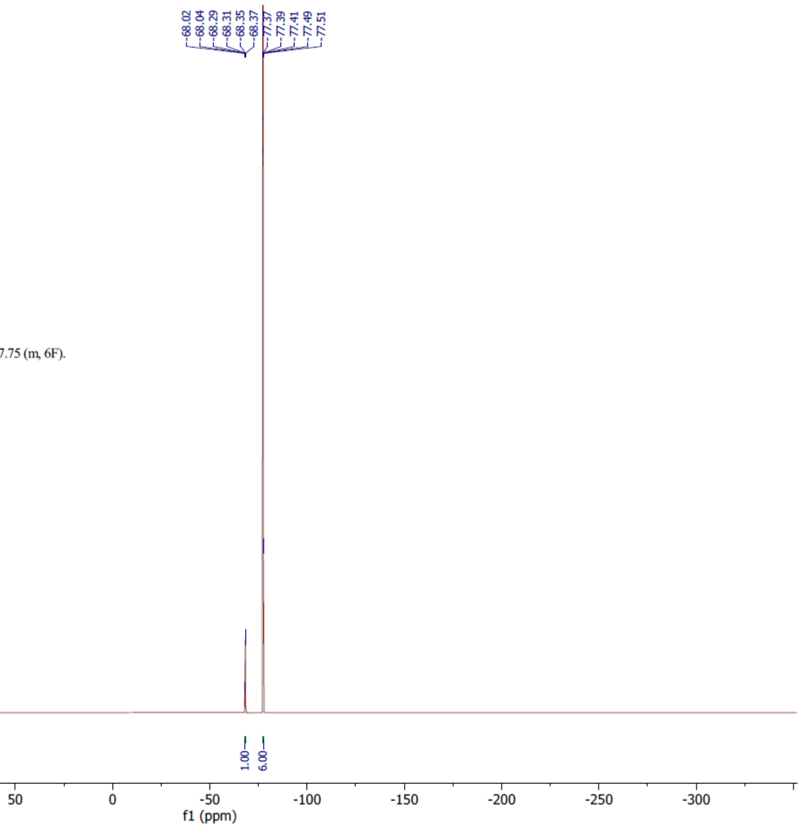

^{19}F NMR (377 MHz, MeOD) δ -67.89 – -68.53 (m, 1F), -77.18 – -77.75 (m, 6F).

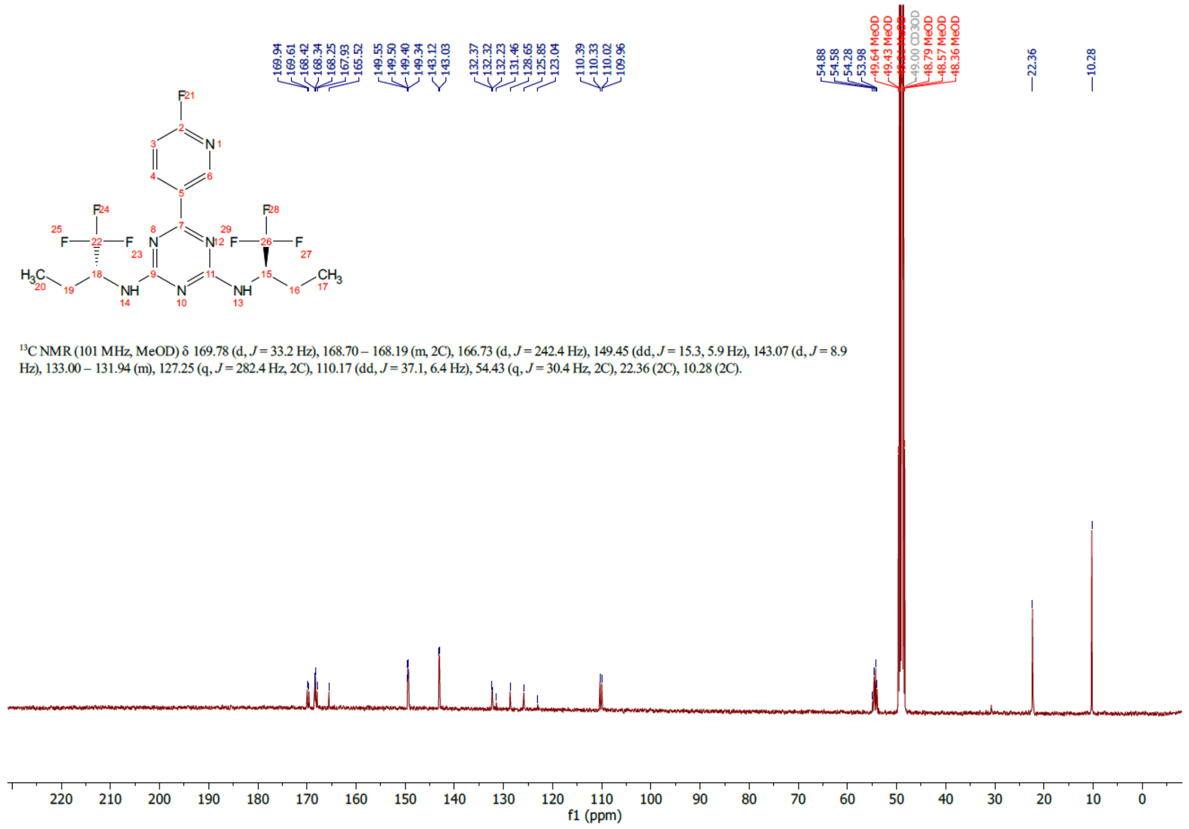

Figure S11. NMR spectra of (*R*)-6-(5-chloro-6-fluoropyridin-3-yl)-*N*[2]-(3,3-difluorocyclobutyl)-*N*[4]-(1,1,1-trifluoropropan-2-yl)-1,3,5-triazine-2,4-diamine (**11**).

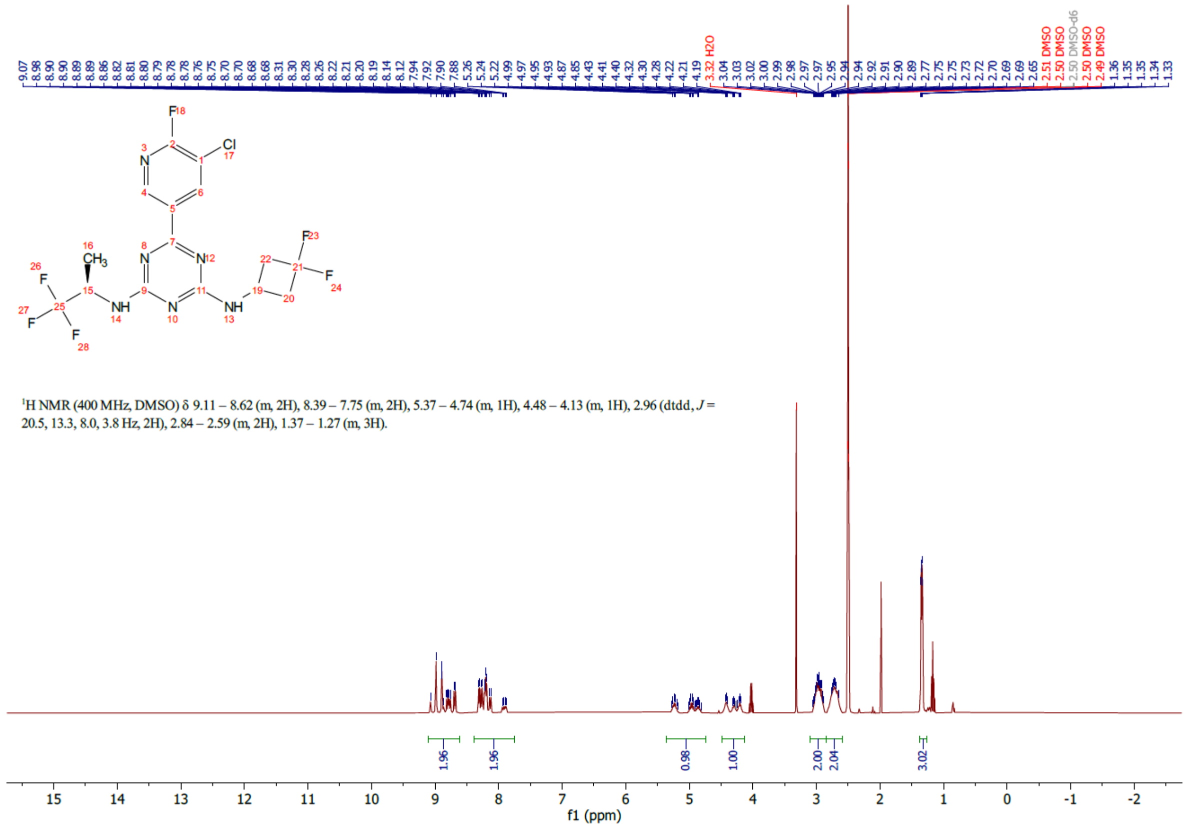

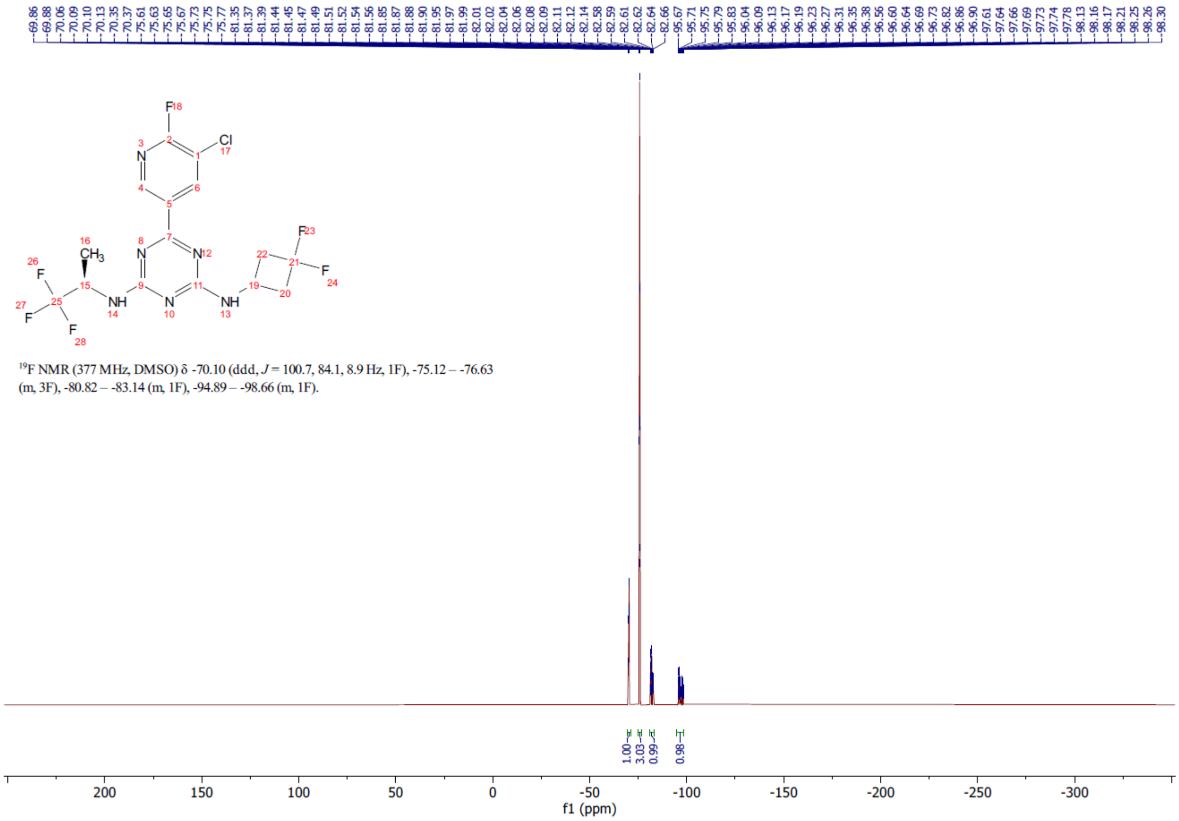

^{19}F NMR (377 MHz, DMSO) δ -70.10 (ddd, J = 100.7, 84.1, 8.9 Hz, 1F), -75.12 – -76.63 (m, 3F), -80.82 – -83.14 (m, 1F), -94.89 – -98.66 (m, 1F).

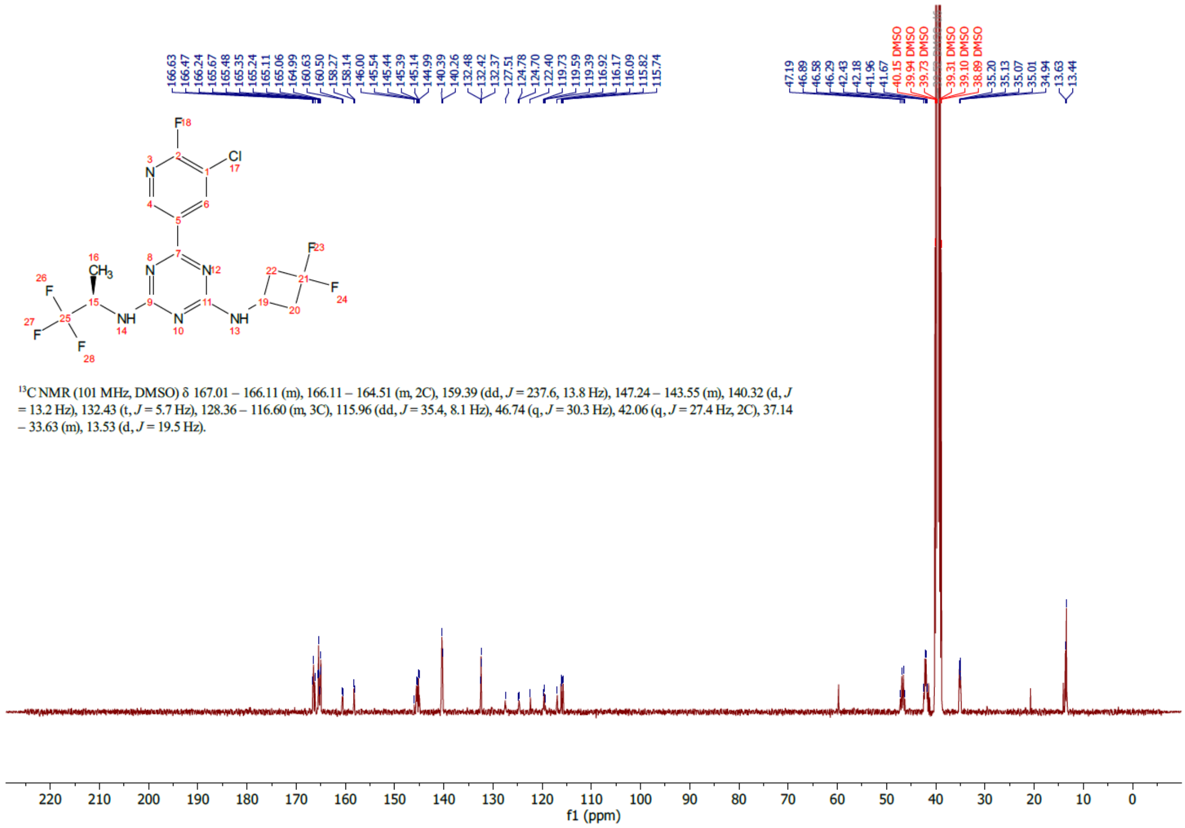

^{13}C NMR (101 MHz, DMSO) δ 167.01 – 166.11 (m), 166.11 – 164.51 (m, 2C), 159.39 (dd, J = 237.6, 13.8 Hz), 147.24 – 143.55 (m), 140.32 (d, J = 13.2 Hz), 132.43 (t, J = 5.7 Hz), 128.36 – 116.60 (m, 3C), 115.96 (dd, J = 35.4, 8.1 Hz), 46.74 (q, J = 30.3 Hz), 42.06 (q, J = 27.4 Hz, 2C), 37.14 – 33.63 (m), 13.53 (d, J = 19.5 Hz).

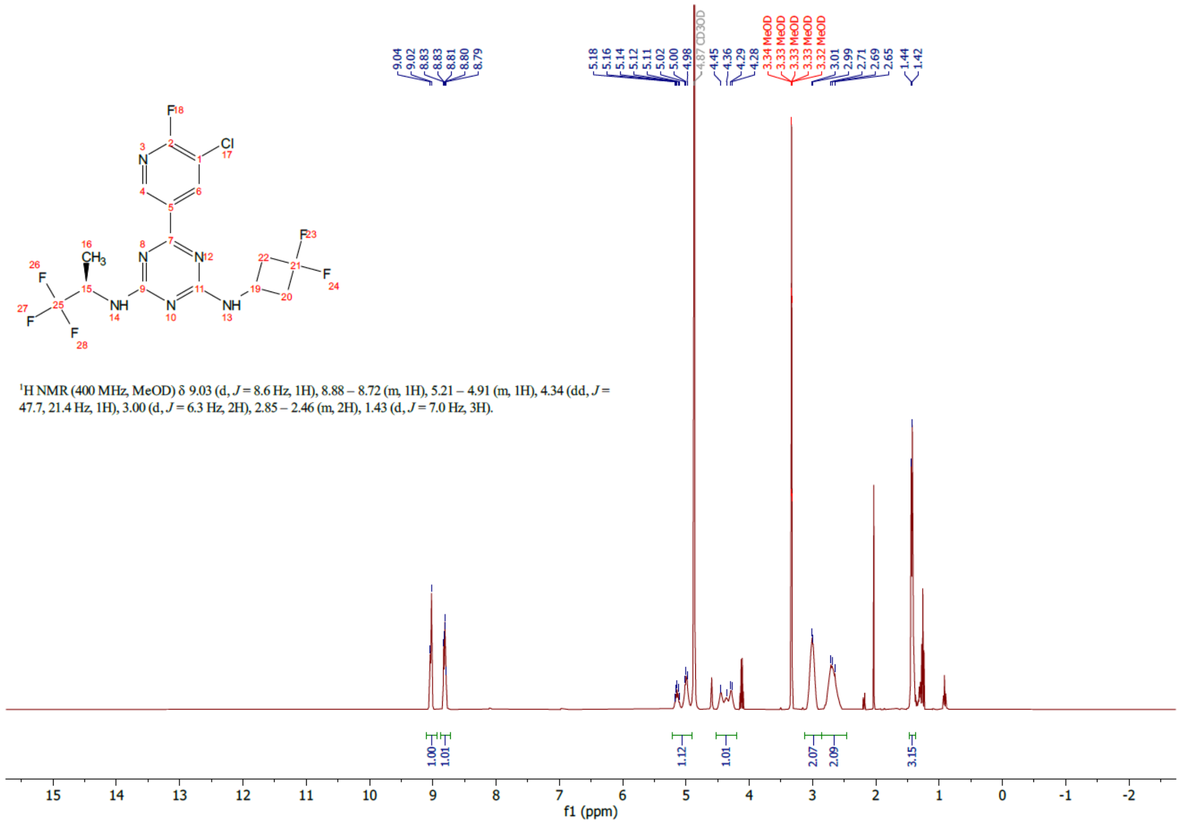

^{1}H NMR (400 MHz, MeOD) δ 9.03 (d, J = 8.6 Hz, 1H), 8.88 – 8.72 (m, 1H), 5.21 – 4.91 (m, 1H), 4.34 (dd, J = 47.7, 21.4 Hz, 1H), 3.00 (d, J = 6.3 Hz, 2H), 2.85 – 2.46 (m, 2H), 1.43 (d, J = 7.0 Hz, 3H).

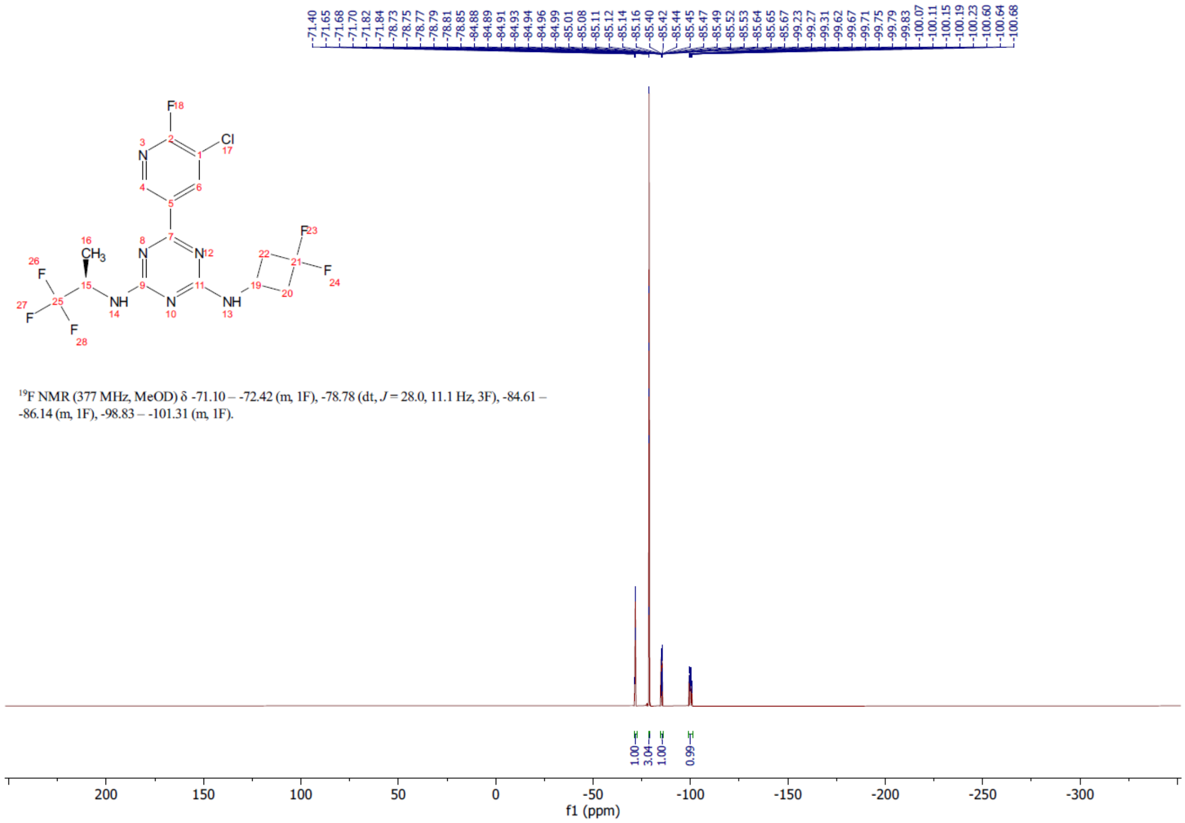

^{19}F NMR (377 MHz, MeOD) δ -71.10 – -72.42 (m, 1F), -78.78 (dt, J = 28.0, 11.1 Hz, 3F), -84.61 – -86.14 (m, 1F), -98.83 – -101.31 (m, 1F).

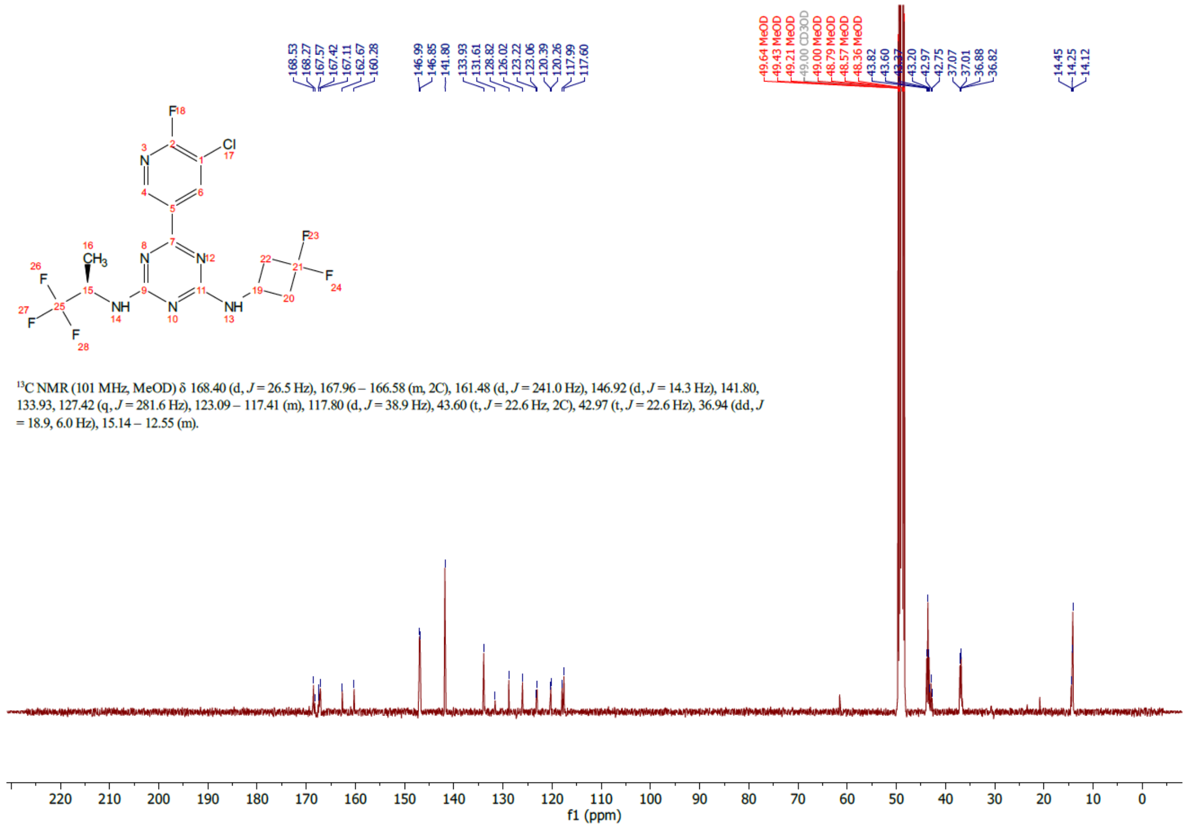

Radiochemistry

Table S1. Investigated reaction parameters for the radiolabeling of the precursor **AG-881** to produce [^{18}F]**6** (RCC determined by radio-TLC after 20 min; n = 1, [a] unless otherwise stated).

^{18}F-agent	Solvent	Temp. (°C)	AG-881 (mg)	RCC (%)[a]
[^{18}F]F^-/K_{222}/K_2CO_3	ACN	100	2	0
	DMSO	140	2	0
	DMSO	180	2	29
	DMSO	190	2	32
	DMF	140	2	0
	DMF	160	2	2
	DMI	200	2	20
[^{18}F]Et_4NF	DMSO	160	2	5 and 7
	DMSO	160	1	5 and 8
	DMSO	180	2	19
	DMSO	180	1	14
	DMSO	190	2	43

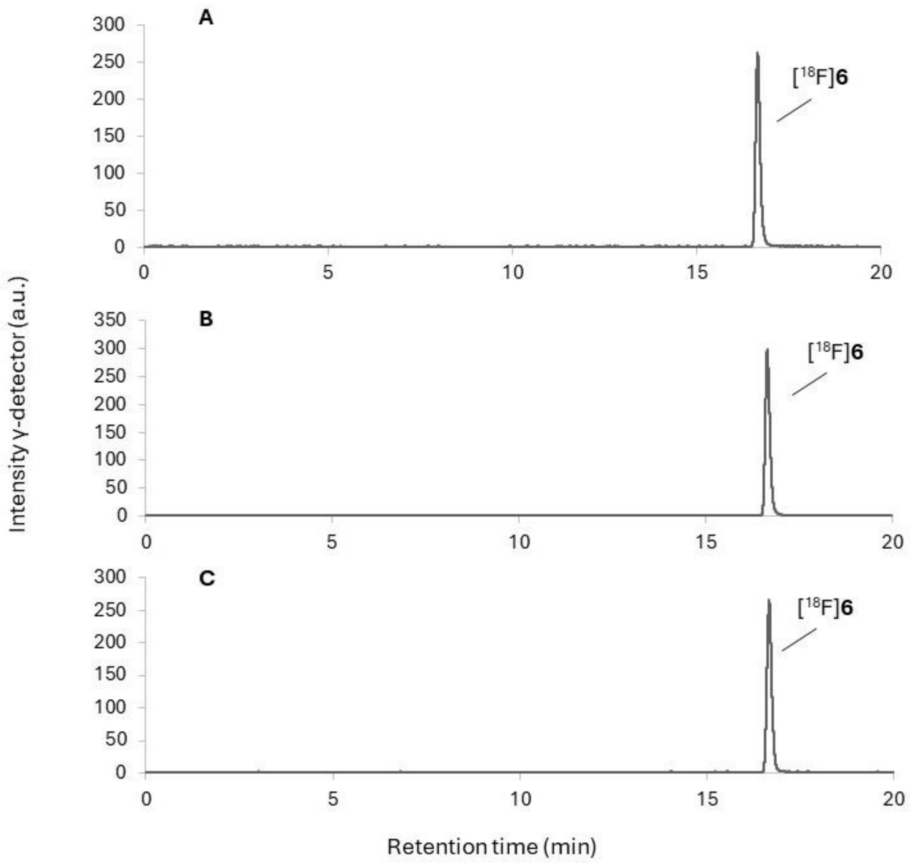

Figure S12. Radio-HPLC chromatograms of [^{18}F]**6** after 4h incubation in isotonic saline (A), PBS (B) and RPMI 1640 (C). Conditions: Reprosil-Pur C18-AQ, 150 x 3.0 mm, 3 µm, gradient with an eluent mixture of MeCN in 20 mM aq. NH_4OAc, 0.4 mL/min.
